# A taxonomic synopsis of *Virola* (Myristicaceae) in Mesoamerica, including six new species

**DOI:** 10.3897/phytokeys.134.37979

**Published:** 2019-10-23

**Authors:** Daniel Santamaría-Aguilar, Reinaldo Aguilar, Laura P. Lagomarsino

**Affiliations:** 1 Shirley C. Tucker Herbarium, Department of Biological Sciences, Louisiana State University, Baton Rouge, LA 70803-1705, USA Louisiana State University Baton Rouge United States of America; 2 Missouri Botanical Garden St. Louis, MO 63166, USA Missouri Botanical Garden St. Louis United States of America; 3 Los Charcos de Osa Centro de Diversidad de Plantas Regionales Osa, Puntarenas, Costa Rica Los Charcos de Osa Centro de Diversidad de Plantas Regionales Osa Puntarenas Costa Rica

**Keywords:** Costa Rica, Flora Mesoamericana, hallucinogenic, Magnoliales, nutmeg, Panama, taxonomy

## Abstract

A taxonomic synopsis of *Virola* (Myristicaceae) is presented for Mesoamerica. Fourteen species are recognised, amongst them six are described and published as new, based on morphology: *V. allenii* D.Santam. & Aguilar, **sp. nov.** from Costa Rica, *V. otobifolia* D.Santam., **sp. nov.** from Panama and *V. amistadensis* D.Santam., **sp. nov.**, *V. chrysocarpa* D.Santam. & Aguilar, **sp. nov.**, *V. fosteri* D.Santam., **sp. nov.** and *V. montana* D.Santam., **sp. nov.** from both Costa Rica and Panama. Additionally, a lectotype is designated for *V. koschnyi*, accompanied by an epitype in view of the fragmentary material. Finally, we recognise *V. laevigata* and *V. nobilis* as morphologically distinct species, though these are frequently considered synonymys of *V. guatemalensis* and *V. surinamensis*, respectively. Of the fourteen accepted species, twelve of them are endemic to Mesoamerica, while the remaining two species (*V. elongata* and *V. sebifera*) extend into South America. Illustrations, species diagnoses and distribution maps for each species are provided, as is an identification key to all species.

## Introduction

Myristicaceae, a member of the magnoliid order Magnoliales (APG IV 2016), comprises 21 genera and nearly 500 species of woody trees, shrubs and, occasionally, lianas (i.e. *Pycnanthus* spp.) By far the most economically important species of the family is *Myristica fragrans* Houtt., the source of the common spices nutmeg and mace. Myristicaceae has a broad pantropical distribution, though most species are found in lowland rainforest habitats. The family is represented in America by about 101 species (Ulloa Ulloa et. al. 2017) in six endemic genera: *Bicuiba* W. J. de Wilde (1 sp.), *Compsoneura* (A. DC.) Warb. (19 spp.), *Iryanthera* Warb. (~20 spp.), *Osteophloeum* Warb. (1 sp./ 2 subsp.; as *Osteophloem* in Smith and Wodehouse 1938), *Otoba* (A. DC.) Karsten (~10 spp.) and *Virola* Aubl. (~60 spp.). Species richness of American Myristicaceae is highest in the lowland forest of the Amazon, where it is considered a hyperdominant family (Smith and Wodehouse 1938; de Wilde 1991; Kühn and Kubitzki 1993; Janovec 2000; Jaramillo et al. 2004; ter Steege et al. 2013; Cardoso et al. 2017).

*Virola* is the largest genus of Myristicaceae in America and, with about 60 species, it is the fourth largest genus in the family. The plants have two particularly distinctive characteristics: their growth form and the mature fruit. The growth form, sometimes called myristicaceous branching (e.g. Gentry 1993), corresponds largely with Massart’s Model (Hallé et al. 1978), which is characterised by a monopodial, orthotropic principal shoot with plagiotropic lateral branches that are developed in regular tiers by rhythmic branching of the main axis (Kühn and Kubitzki 1993). Fruits of *Virola* are distinctive and striking: they dehisce by two valves when ripe, displaying their single seed, which is covered by a brightly coloured laciniate aril that is rich in proteins, lipid and sugars. Birds and monkeys are primary dispersers of these seeds (Howe and Vande Kerckhove 1980; Howe 1981, 1982, 1993; Sabatier 1997; Moreira et al. 2017). Not surprisingly, given the importance of their fruits as a food for wildlife and its abundance in many lowland forests, the ecological importance of *Virola* has been extensively documented (e.g. Frankie et al. 1974; Pitman et al. 2008; Steeves 2011; Riba Hernández 2017). Below, we provide a review of the morphology, biogeography, taxonomic history and ethnobotanical uses of *Virola*.

### Morphology

As with many magnoliids, *Virola* has aromatic tissues. Unlike other magnoliids, though, when the bark is cut or a twig is broken, it yields an exudate that is initially watery and clear and quickly changes to red or pinkish. The leaves, which are exstipulate and simple with entire margins and occasionally have pellucid punctuation, are distichous. Most surfaces of the plant, including leaves, are usually covered with stellate or dendritic trichomes. Staminate inflorescences are paniculate, with few to many branches. Flowers, sometimes reported as fragrant, are unisexual, ebracteolate and inserted on a receptacle; they are produced in lax to dense clusters. Their yellow or brown perianth is connate to varying degrees and uniseriate, usually with 3-lobes. The androecium is compound, with filaments fused into a column, with 3– (4–6) anthers fused to the column. The pistillate inflorescences are shorter than staminate inflorescences. Pistillate flowers have a perianth that is more carnose than those of the staminate flowers and their gynoecia are pubescent and sessile or short- stipitate, topped by a stigma that is usually bilobed (Smith and Wodehouse 1938; de Wilde 1991; Kühn and Kubitzki 1993; Jaramillo et al. 2004).

**Figure 1.**
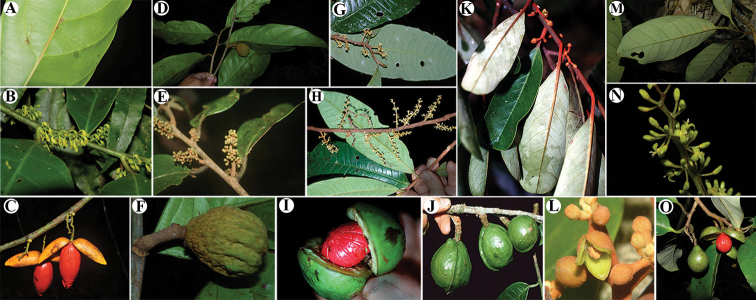
Genera of Myristicaceae present in Mesoamerica **A–C**Compsoneura sect. Compsoneura (*C. excelsa*) **D–F**Compsoneura sect. Hadrocarpa (*C. capitellata*) **G–J***Iryanthera* (**G–I***Iryanthera* spp; **J***I. megistocarpa*) **K–L***Osteophloeum platyspermum***M–O***Otoba novogranatensis*. Photos by Reinaldo Aguilar (**A–C, M–O**), Benjamin Chambi (**E**), Jason D. Wells (**D, F** from http://atrium.andesamazon.org/); Robin Foster (**G–I, K** from https://plantidtools.fieldmuseum.org/en/nlp); Alwyn H. Gentry (**J** from http://www.tropicos.org); and David A. Neill (**L**).

In Mesoamerica, where five of the six native genera of American Myristicaceae occur (Fig. 1), *Virola* can be identified using the following key:

**Table:** 

1	Leaf blades with tertiary veins conspicuous and more or less parallel and perpendicular to the lateral veins	***Compsoneura*** (Fig. 1A–F)
–	Leaf blades with tertiary veins inconspicuous or, when conspicuous, not as above	**2**
2	Leaf blades with malpighiaceous trichomes on abaxial surface	**3**
–	Leaf blades with stellate, dendritic, or irregularly stellate trichomes on abaxial surface	**4**
3	Vernation convolute; abaxial surface of leaf blades without hyaline crystals; flowers with bracteoles; fruits transversally ellipsoid, if globose, the pericarp 7–8 mm thick; seeds not gibbose at the apex	***Iryanthera*** (Fig. 1G–J)
–	Vernation conduplicate; abaxial surface of leaf blades with hyaline crystals; flowers without bracteoles (in Mesoamerica); fruits longitudinally globose to subglobose; seeds usually gibbose near the apex	***Otoba*** (Fig. 1M–O)
4	Trichomes on abaxial leaf surface stellate, concolorous; inflorescences sessile, with 1–2 (–3) main branches; flowers with bracteoles; stamens 10 (in Mesoamerica); fruits transversally ellipsoid	***Osteophloeum*** (Fig. 1K–L)
–	Trichomes on abaxial leaf surface dendritic, irregularly stellate or if stellate, the central part of the trichome reddish to reddish-clear, constrasting in colour with the hyaline branches; inflorescences distinctly pedunculate, with 1 main branch; flowers without bracteoles; stamens 3–7; fruits ellipsoid, globose, ovoid or subglobose	*** Virola ***

### Biogeography

*Virola* is found throughout tropical America. Species have been collected from Mexico to southern Brazil, though are notably absent in El Salvador. Occurrence in the Caribbean is limited: one species is found in the West Indies (*V. surinamensis* [Rol. ex Rottb.] Warb.), though a fossil *Virola* flower, recently described from Dominican Republic (Poinar and Steeves 2013), suggests that genus has persisted over geological time in the region. Species richness is highest in western Amazonia, with the greatest concentration of species in Brazil (~35 spp.), Colombia (~ 29 spp.) and Peru (~ 23 spp.), where they are found mainly in humid forests, though some species grow in dry formations (e.g. *V. sessilis* [A. DC.] Warb., *V. subsessilis* [Benth.] Warb.) (Rodrigues 1980, 2015; Gradstein 2016; Vásquez et al. 2018).

**Figure 2.**
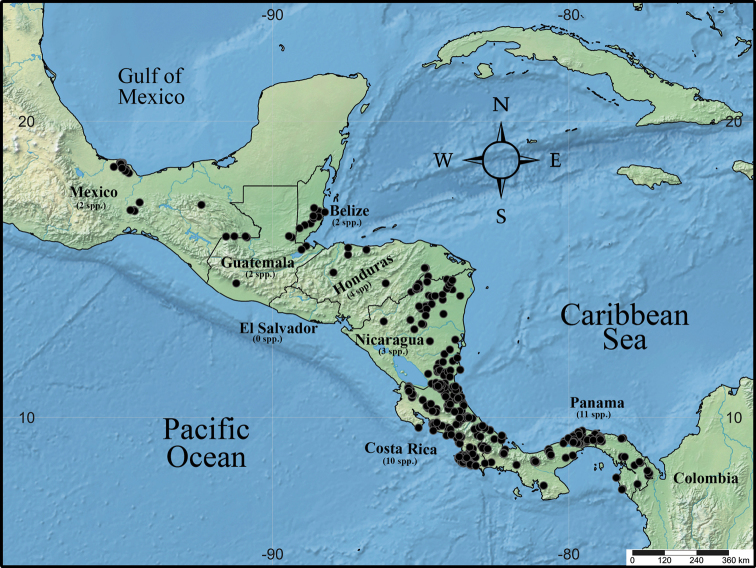
Geographic distribution of *Virola* in Mesoamerica, with the number of species native to each country indicated in parentheses below the country’s name.

Within Mesoamerica, the greatest concentration of *Virola* species occurs in Costa Rica and Panama. Together, these two countries contain 10 and 11, respectively, of the 14 Mesoamerican species (Fig. 2). Of the 14 species treated in this synopsis, 12 are endemic to Mesoamerica, while *V. elongata* (Spruce ex Benth.) Warb. occurs from Panama to Brazil and *V. sebifera* Aublet from Honduras to Brazil; both of these species are widespread in South America. The majority of *Virola* species occupy lowland wet forest below 800 m elevation, with a few species reaching montane forests up to 2000 m elevation (e.g. *V. montana*). A single montane species of *Virola* can occur on both slopes, though, within a country or region, there is a tendency to find them on only one, more often the Caribbean slope (e.g. *V. fosteri*, *V. amistadensis*, *V. multiflora*). A list of the current species of *Virola* in Mesoamerica with geographic distribution by country, elevations and slope is given in Table 1.

**Table 1.** List of the 14 accepted species of *Virola* in Mesoamerica with countries of occurrence, slope preference and elevation within Mesoamerica.

Species	Distribution	Slope	Elevations (m)
*V. allenii* D. Santam. & Aguilar.	Costa Rica	Pacific	0–350 (–1350)
*V. amistadensis* D. Santam.	Costa Rica, Panama	Caribbean	650–1200
*V. chrysocarpa* D. Santam. & Aguilar.	Costa Rica, Panama	Pacific	0–700
*V. elongata* (Benth.) Warb.	Panama	Caribbean	50–450
*V. fosteri* D. Santam.	Costa Rica, Panama	Caribbean	0–350 (–800)
*V. guatemalensis* (Hemsl.) Warb.	Mexico, Guatemala, Honduras	Both slopes	150–1250
*V. koschnyi* Warb.	Mexico, Guatemala, Belize, Honduras, Nicaragua, Costa Rica, Panama	Both slopes, mainly on the Caribbean side	10–1000 (–1700)
*V. laevigata* Standl.	Costa Rica, Panama	Pacific	0–500 (1600?)
*V. megacarpa* A. H. Gentry.	Panama	Caribbean	50–550
*V. montana* D. Santam.	Costa Rica, Panama	Both slopes	700–2000
*V. multiflora* (Standl.) A. C. Sm.	Belize, Honduras, Nicaragua, Costa Rica	Caribbean	0–650 (–1400)
*V. nobilis* A. C. Sm.	Costa Rica, Panama	Both slopes	0–400 (–1300)
*V. otobifolia* D. Santam.	Panama	Caribbean	50–850
*V. sebifera* Aubl.	Honduras, Nicaragua, Costa Rica, Panama	Both slopes	0–1000

### Ethnobotany

*Virola* has diverse ethnobotanical uses and there are many reports of local use of several species of *Virola* for many non-medicinal purposes. For example, the oils from the seeds of *Virola* (e.g. *V. guatemalensis*, *V. sebifera*) are used as lubricant in machinery and to make soap and candles that emit intense light, produce little smoke and smell pleasant; Pesce 2009). Instead of using the extracted oils, indigenous people of the Talamanca mountains of Costa Rica (Pittier 1978) and the Cuna tribe of Panama (Duke 1975) burn whole seeds as candles. Wood from many species, including *Virola fosteri*, *V. koschnyi*, *V. sebifera* and *V. surinamensis*, is used for construction and carpentry, to make matchsticks, as pulp for paper and to manufacture plywood (Rodrigues 1972; Flores-Vindas and Obando-Vargas 2014; Pérez and Condit 2018). The seeds and arils of *Virola guatemalensis*, a common shade tree in coffee plantations in Veracruz, Mexico (Sánchez Hernández et al. 2017), are used to flavour chocolate drinks and other beverages (Warburg 1897; Standley and Steyermark 1946; Pennington and Sarukhánn 2016).

*Virola* also produces many chemicals that are biologically active in humans. For example, *Virola* is perhaps best known for its hallucinogenic properties, which are often incorporated into indigenous cultural practices of South America. The hallucinogen is usually obtained from the exudate of the inner bark of several species, including: *Virola calophylla* Warb., *V. calophylloidea* Markgr., *V. duckei* A. C. Sm., *V. elongata*, *V. sebifera*, *V. surinamensis* and *V. theiodora* Warb.; it has also been documented in *Iryanthera* and *Osteophloeum platyspermum* (Spruce ex A. DC.) Warb. (Schultes 1954, 1969, 1979; Schultes and Holmstedt 1968; Prance 1970; Soares Maia and Rodrigues 1974; Bennett and Alarcón 1994). In addition to being psychoactive, the reddish exudate of various *Virola* species is thought to have medicinal properties. For example, the sap of *V. melinonii* (Benoist) A. C. Sm. and *V. surinamensis* is applied to treat skin conditions, alleviate tooth pain, soothe colic and as a styptic (i.e. a substance used to stop bleeding) to treat ulcerating sores and wounds, haemorrhoids and other sources of bleeding (Plotkin and Schultes 1990). The sap of an undetermined species of *Virola* has been reported to be used as a contraceptive by Indians on the Rio Negro of Brazil and is reputedly effective for a period of two or three years (Plotkin and Schultes 1990). Finally, the oil obtained from the seeds of *Virola officinalis* Warb, *V. sebifera* and *V. surinamensis* has many medical folk uses, including treatments for asthma, rheumatism, tumours of the joints, intestinal worms, skin diseases, erysipelas, haemorrhoids and bad breath (Rodrigues 1972, 1980; Plotkin and Schultes 1990).

### Taxonomic history

*Virola* was first described by Aublet in “Histoire des plantes de la Guiane Françoise”, based on *V. sebifera* Aublet (Aublet 1775). It is likely that the etymology of the generic name is derived from one of the common names mentioned in the protologue, which stated: “Nomen Caribaeum Oyapocenfibus *VOIROUCHI*; Sinemarienfibus *VIROLA*; Galliucum *JEAJEAMADOU*” (Aublet 1775). Subsequent authors have attributed this etymology to a Galibi or Sinemari vernacular name for *V. sebifera* in French Guiana (Rodrigues 1972, 1980; Cardona et al. 2010; Mari Mut 2018). However, others believe that the name is derived from the Latin *viriola* (bracelet), in reference to the aril that surrounds the seed (e.g. Flores 2010).

There is a long taxonomic history to the American species of Myristicaceae. This began with George Bentham (1853), who was the first to publish on these species in “Notes on the American Species of Myristica”. In this treatment, he recognises 12 species of *Myristica* (nine of them new) and places these in three different groups, characterised by differences in the androecium (i.e. number of anthers and shape, thickness and length of the column) and leaf venation (i.e. number of veins and their degree of separation). Alphonse de Candolle published the second and broadest treatment of Myristicaceae, including species from both the New and Old World (de Candolle 1856). He recognised ca. 84 species, which he divided into 13 sections in a single genus, *Myristica*, which included two sections that contain species today recognised as *Virola*: Virola (Sect. III/ 8 spp.) and Sychoneura (Sect. IV/10 spp.). In this work, he described 12 new taxa that now belong to *Virola*. The British botanist William Botting Hemsley was the earliest author to publish an account of the Mesoamerican species of Myristicaceae in “Biologia centrali-americana” (Hemsley 1882). In this work, three species are newly described: *Myristica guatemalensis* Hemsl., *M. mexicana* Hemsl. [=*Compsoneura mexicana* (Hemsl.) Janovec] and *M. panamensis* Hemsl. The most comprehensive treatment of Myristicaceae during this era was produced by the German botanist and agriculturalist Otto Warburg in his “Monographie der Myristicaceen” (Warburg 1897). Warburg recognised 240 species in 15 genera, including *Virola* (recognised for the first time as a genus). Warburg’s concept of *Virola* included 27 species in two sections: *Oxyanthera* Warb. (13 spp.; anthers longer than the column) and *Amblyanthera* Warb. (14 spp.; anthers equal to or shorter than the column). Amongst the *Virola* species that Warburg recognised were two previously recognised as *Myristica*: *V. guatemalensis* (Hemsl.) Warb. and *V. panamensis* (Hemsl.) Warb. Following the publication of Warburg’s treatment, generic recognition of *Virola* became broadly accepted and, subsequently, five new names were published for Mesoamerica: *V. koschnyi* Warb. (Warburg 1905), *V. merendonis* Pittier, *V. warburgii* Pittier (Pittier 1916, 1922), *V. laevigata* Standl. and *V. brachycarpa* Standl. (Standley 1929, 1932). These names and species identities are followed in regional taxonomic treatments (e.g. Standley 1928, 1930, 1931). “The American species of Myristicaceae” (Smith and Wodehouse 1938) by Albert C. Smith, with contributions of pollen analysis by Roger P. Wodehouse was the next contribution to the taxonomy of Mesoamerican Myristicaceae. These authors proposed the division of *Virola* into six groups: *Mollissimae* (I), *Sebiferae* (II), *Calophyllae* (III), *Rugulosae* (IV), *Surinamenses* (V), and *Subsessilis* (VI). The characters used to delimit these groups included trichome type, degree of perianth connation, spacing of secondary leaf veins, column shape and anther apex shape. Of the 38 species of *Virola* recognised in Smith and Wodehouse (1938), the following proposals pertain to Mesoamerican species: (1) *V. panamensis*, *V. warburgii*, *V. merendonis* and *V. laevigata* were sunk into synonymy with *V. sebifera*, *V. sebifera*, *V. koschnyi* and *V. guatemalensis*, respectively; (2) the description of *V. nobilis* A. C. Smith from Panama; and (3) the transfer of *Dialyanthera multiflora* Standl. to *Virola multiflora* (Standl.) A. C. Smith and the designation of *V. brachycarpa* Standl. as synonymous with this species.

Since then, 17 new species of *Virola* have been published from South America (Ducke 1945; Smith 1953, 1956; Williams 1964; Little 1970; Rodrigues 1977, 1989; Sabatier 1997; Jaramillo et al. 2000) and one from Mesoamerica: *V. megacarpa* A. H. Gentry (Gentry 1975). Several important local floristic accounts of *Virola* have also been published, some of which made important taxonomic changes for their region (South America: Cogollo 2011, Gradstein, 2018 [Colombia]; Achá and Liesner 2014 [Bolivia]; Rodrigues 1980, 2010, 2015 [Brazil]; Jaramillo et al. 2004 [Ecuador]; Mitchell 2002 [central French Guiana]; Aymard et al. 2007 [Guiana Shield]; Vásquez Martínez 1997, 2010, Vásquez et al. 2018 [Peru]; Rodrigues 2008, Rodrigues et al. 2001 [Venezuela]; Mesoamerica: Acevedo-Rodríguez and Strong 2012 [West Indies]); (Balick et al. 2000 [Belize]; Jiménez 2007 [Costa Rica]; Nelson 2008 [Honduras]; Villaseñor 2016 [Mexico]; Gentry 2001 [Nicaragua]; Duke 1962, Correa et al. 2004 [Panama]). Perhaps the most substantial of these is “Revisão taxonômica das espécies de *Virola* Aublet (Myristicaceae) do Brasil” (Rodrigues 1980), which provided a treatment for Brazil and refuted the infrageneric proposals by previous authors.

*Virola* has been the subject of limited phylogenetic analysis. The first molecular phylogenetic study of *Virola*– and the only to date– demonstrated that the genus is divided into two large subclades, informally called “Multinervae” and “Sebiferae” (Steeves 2011). Morphologically, Multinervae is composed of tall emergent canopy trees that typically have more numerous secondary leaf veins, thicker pericaps and arils that are lacinate throughout their length, compared to the sub-canopy to canopy species of Sebiferae that have fewer secondary veins, thin pericarps and arils that are only lacinate for half of their length. This phylogeny, or future improved phylogenetic hypotheses, has the potential to provide information for future infrageneric reclassification of *Virola*.

## Materials and methods

This work was undertaken as part of the “Flora Mesoamericana” project (http://www.tropicos.org/Project/FM) by the first author. However, our definition of “Mesoamerica” is different than that used in the *Flora*; here, it refers to the portion of Central America and southern Mexico from the Isthmus of Tehuantepec in Mexico in the north to the Panamanian-Colombian border in the south.

Approximately 500 physical herbarium specimens of Mesoamerican *Virola* were examined for this study from the following herbaria: CR (including ex INB), LSCR, LSU, MO, NO and USJ (acronyms follow Thiers [continuously updated]), though specimens from MO represent the majority of the material studied. All type specimens, as well as general collections, hosted by virtual herbaria, were consulted, including those maintained by: the Field Museum (**F**; http://emuweb.fieldmuseum.org/botany/taxonomic.php), Instituto Nacional de Pesquisas da Amazônia (**INPA**; http://inct.florabrasil.net/en/), JSTOR Global Plants (http://plants.jstor.org), Museum of Natural History, Paris (**P**; http://www.mnhn.fr), Oberösterreichischen Landesmuseums (**LI**; https://www.europeana.eu/portal/es), University of Maryland (**MARY**; http://intermountainbiota.org/portal/), Universidad Nacional Autónoma de México (**MEXU**; https://datosabiertos.unam.mx/biodiversidad/) and the National Herbarium of The Netherlands (**U**; http://herbarium.naturalis.nl/). Images of specimens not available online were provided by: EAP, F, HUH, NY, PMA, SCZ and US. When specimens were only studied digitally, they are denoted by an asterisk following the herbarium acronym (e.g. EAP!*). Label information of specimens studied and housed at Museo Nacional de Costa Rica (**CR**) and Missouri Botanical Garden (**MO**) are available online via the ECOBIOSIS (http://ecobiosis.museocostarica.go.cr/index.aspx), HERBARIO (http://www.specify7.museocostarica.go.cr:8080/specify-solr/Herbario/) and TROPICOS (http://www.tropicos.org/) databases.

Species descriptions are based primarily on herbarium specimens, though observations during fieldwork by the first and second authors, especially in the Osa Peninsula of Costa Rica, were also important. If necessary and material permitted, flowers from herbarium specimens were rehydrated before measurement. A ruler was used to measure leaves and inflorescences; a digital Neiko caliper was used to measure fruits and seeds, as well as the thickness of the twigs, petioles and peduncles; and, finally, flowers, trichomes and thickness of the pericarp were measured with a micrometer calibration tool (1div = 1mm) under a dissecting stereoscope (Bausch & Lomb).

The preliminary conservation status of each new species was assessed using quantitative criteria recommended by the IUCN Red List (IUCN Standards and Petitions Subcommittee 2014). Georeferenced specimen data were used to determine the area of occupancy (AOO) and the extent of occurrence (EOO), which in turn were used to determine threat status. All analyses were performed in the R package conR (Dauby et al. 2017). When the recommendation differed between AOO and EOO assessments for a given species, we opted to conservatively recommend the more vulnerable status, following Knapp (2013).

Specimens cited are listed first by country in a north to south sequence. Within country, specimens are listed alphabetically by major division and then alphabetically by province or department and, finally, in alphabetical order by the collector’s surname. When the coordinates and/or elevation were not included on the herbarium label, but were present in the TROPICOS database, the values from TROPICOS are included. Dot-distribution maps were compiled from studied specimens and generated with SimpleMappr (Shorthouse 2010).

In the nomenclatural section for each new species, we cite both accession numbers and barcodes when present. Barcodes are included in square brackets and follow the format of a series of numbers preceded by a herbarium acronym (e.g. [GH00039891]); all other numbers correspond to accession numbers.

Knoweldge of the group was improved by numerous field trips since 1991 throughout Costa Rica, especially in the Osa Peninsula and La Selva Biological Station, by the second author and, more recently, by the first author in the same region.

## Key to the species of *Virola* in Mesoamerica

**Table:** 

1	Abaxial leaf surface with pediculate trichomes (sometimes sessile in *V. sebifera* and with short pedicle in *V. elongata*) (Fig. 3C, G, I, J, N)	**2**
–	Abaxial leaf surface with sessile trichomes (Fig. 3 A, B, D, E, F, H, K–M, O)	**5**
2	Leaf blades with 9–17 lateral veins per side, free or slightly anastomosing near the margin and without forming a very marked intramarginal vein, indument on abaxial surface caducous; petiole slightly canaliculate; staminate flower with the filament column 0.1–0.4 mm long, anthers with apiculus 0.1–0.2 mm long; fruits 1.1–1.7 (–2.3) × 0.8–1.3 (–1.6) cm, globose to subglobose (Fig. 4K); pericarp 0.3–0.6 (–0.9) mm thick.	***V. sebifera***
–	Leaf blades with (16–) 20–50 lateral veins per side, anastomosing near the margin and forming a conspicuous intramarginal vein, indument on abaxial surface persistent; petiole markedly canaliculate; staminate flower with the filament column 0.7–1.5 mm long, anthers without apiculus or this very inconspicuous; fruits 1.9–5.7 × 1.5–2.9 cm, ellipsoid, ovoid-ellipsoid or sometimes subglobose (Fig. 4 E, F, P); pericarp 1.2–6 mm thick	**3**
3	Leaf blades with (32–) 40–50 lateral veins per side; fruits 4–5.7 × 2–2.9 cm, the apex acuminate to rostrate (Fig. 4P); pericarp 3–6 mm thick	***V. megacarpa***
–	Leaf blades with (16–) 20–35 lateral veins per side; fruits 1.9–3.1 × 1.5–1.9 cm, the apex acute to apiculate or obtuse; pericarp 1.2–3.1 mm thick	**4**
4	Mature leaf blades on adaxial surface pubescent and asperous to the touch in dry specimens, abaxial surface densely hirsute to hirsutulous, trichomes with 3–6 branches, the branches 0.2–0.6 mm long; staminate flowers with filament column 1.3–1.5 mm long	***V. chrysocarpa***
–	Mature leaf blades on adaxial surface glabrous to glabrescent and smooth to the touch in dry specimens, abaxial surface densely tomentose, trichomes with 4–10 branches, the branches 0.1–0.2 mm long; staminate flowers with filament column 0.7–0.9 (–1.4) mm long	***V. koschnyi***
5	Leaf blades with 9–20 lateral veins per side, (0.8–) 1–3 cm apart; staminate flowers with perianth infundibuliform, the filament column 0.2–0.9 (–1) mm long; fruits green when mature; aril laciniate in very narrow bands distally when dry, membranaceous	**6**
–	Leaf blades with 10–34 lateral veins per side, 0.2–1.1 (–1.5) cm apart; staminate flowers with perianth campanulate, globose or subglobose, the filament column 0.6–1.3 mm long; fruits yellow, orange or a combination of these colours when mature; aril laciniate in narrow bands distally when dry, coriaceous	**9**
6	Abaxial leaf surface sparsely pubescent, trichomes concolorous, the trichome branches 0.1–0.2 mm long; peduncle of staminate inflorescences 0.07–0.15 cm thick; staminate flower with the perianth 1.5–1.9 mm long; fruits 1.6–1.9 × 0.9–1.1 cm (Fig. 4L), the line of dehiscence canaliculate or smooth; pericarp 0.5–0.7 mm thick	***V. elongata***
–	Abaxial leaf surface densely pubescent, trichomes dicolorous (the central part usually reddish, contrasting in colour with the hyaline to pale reddish branches), the trichome branches ± 0.01–0.05 mm long; peduncle of staminate inflorescences 0.09–0.34 cm thick; staminate flower with the perianth (1.5–) 2–2.8 mm long; fruits 2.1–4.5 × 1.5–2.9 cm (Fig. 4A–C), the line of dehiscence (generally) carinate; pericarp 1–4.7 mm thick	**7**
7	Leaf blades elliptic to widely elliptic; staminate flowers with the filament column 0.2–0.4 mm long; fruits with pericarp 1–2 mm thick	***V. amistadensis***
–	Leaf blades oblong-elliptical or rarely elliptical; staminate flowers with the filament column 0.5–1 mm long; fruits with pericarp (2.7–) 3–4.7 mm thick	**8**
8	Leaf blades 3.2–7.3 cm wide; lateral veins 15–20 per side, with 4–5 per 5 cm, 1.2–1.8 cm apart; staminate flowers in dense terminal fascicles; staminate perianth lobes glabrous or sparsely pubescent on the adaxial margin; filament column 0.5–0.6 mm long, thin throughout its length (sometimes slightly thickened at the base), not constricted at the apex; mature fruits with trichomes that fall like dust	***V. allenii***
–	Leaf blades (4.1–) 7.3–14.2 cm wide; lateral veins 10–16 per side, with 2–4 per 5 cm, 1.7–3 cm apart; staminate flowers in lax terminal fascicles; staminate perianth lobes pubescent adaxially; filament column 0.9–1 mm long, conspicuously thickened throughout its length, except constricted at the apex; mature fruits with persistent trichomes	***V. otobifolia***
9	Abaxial leaf surface with caducous trichomes or practically glabrous or, if some persistent trichomes, then along the veins, never on the entire surface	**10**
–	Abaxial leaf surface with trichomes persistent throughout	**11**
10	Twigs and petioles glabrous; leaf blades with 12–20 lateral veins per side; staminate flowers with the filament column 0.8–1.3 mm long; fruits 1.8–2.9 × 1.5–1.8 cm (Fig. 4M), glabrous to glabrescent or very sparsely pubescent, the line of dehiscence canaliculate or smooth; pericarp 1.8–2.8 mm thick; 0–500 (1600?) m elevation	***V. laevigata***
–	Twigs and petioles pubescent; leaf blades with (15–) 18–30 lateral veins per side; staminate flowers with the filament column 0.6–0.9 mm long; fruits (2.8–) 3–3.6 × 2–2.5 cm (Fig. 4H), densely tomentose to tomentulose, the line of dehiscence carinate; pericarp 3.2–5 mm thick; 700–2000 m elevation	***V. montana***
11	Leaf blades 1.4–3.6 (–4.8) cm wide	**12**
–	Leaf blades (2.4–) 3.8–7.1 (–8.9) cm wide	**13**
12	Leaf blades with the base revolute; lateral veins 16–24 per side, with 10–15 veins per 5 cm, 0.2–0.5 (–0.7) cm apart; staminate flowers with the filament column 0.9–1.3 mm long, anthers 0.6–0.9 mm long; fruits 1.5–2.3 × 1.2–1.8 cm (Fig. 4I), pericarp 1.5–2.5 mm thick	***V. fosteri***
–	Leaf blades with the base not revolute; lateral veins 10–18 per side, with (6–) 8–10 veins per 5 cm, 0.4–0.8 (–1.2) cm apart; staminate flowers with the filament column 0.7–1 mm long, anthers 0.3–0.6 mm long; fruits 1.3–1.9 × 0.9–1.2 (–1.4) cm (Fig. 4J), pericarp 0.7–1 mm thick	***V. multiflora***
13	Lateral veins 13–21 per side, slightly elevated or flat on the abaxial leaf surface, tertiary veins almost indistinct or very slightly visible on both surfaces (Fig. 3F) ; fruits commonly glabrous or glabrous distally and pubescent at base or scattered and inconspicuously pubescent; pericarp 0.4–1 (–2.5) mm thick	***V. guatemalensis***
–	Lateral veins 20–30 (–32) per side, markedly elevated on the abaxial leaf surface, tertiary veins usually distinct on both surfaces (especially abaxially) (Fig. 3L); fruits densely tomentose to tomentulose; pericarp 2.3–3.5 (–4.2) mm thick	***V. nobilis***

**Figure 3.**
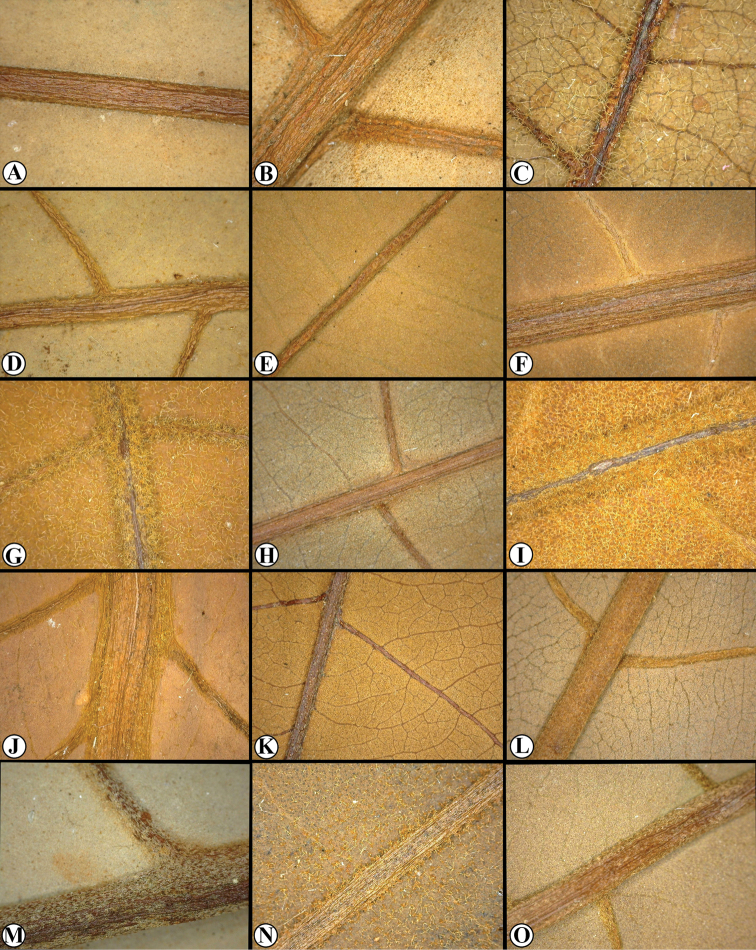
Trichomes in Mesoamerican *Virola***A***Virola allenii* (*R. Aguilar 2224*, MO) **B***V. amistadensis* (*G. McPherson 8703*, MO) **C***V. chrysocarpa* (*O. Valverde 970*, MO) **D***V. elongata* (*G. McPherson 13674*, MO) **E***V. fosteri* (*G. McPherson 20913*, MO) **F***V. guatemalensis* (*R. Cedillo 3349*, MO) **G***V. koschnyi* (*R. E. Gereau et al. 3455*, MO) **H***V. laevigata* (*R. Aguilar 2004*, MO) **I***V. megacarpa* (*J. A. Duke 15261*, MO) **J***V. montana* (*E. Bello 855*, MO) **K***V. multiflora* (*R. Rueda et al. 8196*, MO; inset, *J. C. Sandino 3302*, MO) **L***V. nobilis* from Barro Colorado (*R. J. Schmalzel 320*, MO) **M***V. otobifolia* (*G. de Nevers & H. Herrera 7056*, MO) **N***V. sebifera* (*F. Almeda et al. 5553*, MO) **O***V. nobilis* from Osa Peninsula (*R. Aguilar 11186*, MO).

**Figure 4.**
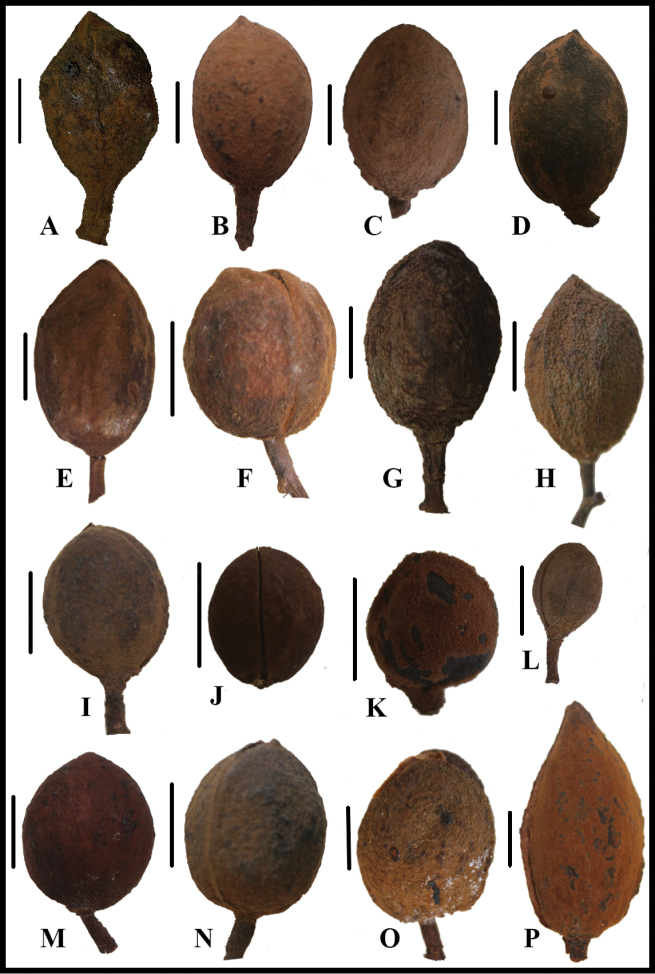
Fruits of Mesoameican *Virola*, organised in groups of morphologically similar species **A***V. allenii* (*P. H. Allen 6727*, GH) **B***V. amistadensis* (*G. McPherson 9715*, MO) **C***V. otobifolia* (*A. Gentry & S. Mori 14199*, MO) **D***V. macrocarpa* (*A. E. Lawrance 675*, MO) **E***V. chrysocarpa* (*B. Hammel et al.16864*, MO) **F***V. koschnyi* (*R. Robles 1587*, MO) **G***V. guatemalensis* (*S. Sinaca 1542*, MO) **H***V. montana* (*J. Utley & K. Utley 5054*, MO) **I***V. fosteri* (*S. Mori & J. Kallunki 4891*, MO) **J***V. multiflora* (*R. Rueda et a.l 9960*, MO) **K***V. sebifera* (*T. B. Croat 5959*, MO) **L***V. elongata* (*J. D. Dwyer & M. Correa 8420*, MO) **M***V. laevigata* (*R. Aguilar 1585*, MO) **N***V. nobilis* from Barro Colorado (*T. B. Croat 8090*, MO) **O***V. nobilis* from the Osa Peninsula (*G. Herrera 4222*, MO) **P***V. megacarpa* (*G. de Nevers 5184*, MO). Scale bars: 1 cm.

## Taxonomy

### 1. Virola allenii

Taxon classificationPlantaeMagnolialesMyristicaceae

D.Santam. & Aguilar sp. nov.

8BF8C77C-5973-5206-B8A3-A8C72DCE898F

urn:lsid:ipni.org:names:77202542-1

Figs 5 , 6 , 7A

#### Diagnosis.

Species resembling *Virola macrocarpa* in its leaf blades that are whitish on the abaxial side and covered with stellate, sessile trichomes with the centre reddish and contrasting with the hyaline branches to reddish-clear in colour and lateral veins that are not densely arranged, as well as large fruits that are covered with ferruginous trichomes. It differs in its narrow leaf blades (3.2–7.3 cm vs. 7–11 cm wide) with acute or obtuse to rounded bases (vs. broadly obtuse), fruits with thick pericarp (3.2–3.8 mm vs. 1.8–3 mm thick) and preference for humid lowland forests at 0–350 (–1350) m elevation (vs. montane forests in Andes of Colombia at around 1100 m elevation).

#### Type.

Costa Rica. Puntarenas: Esquinas forest preserve, 0 m, 10 Jan 1951 (♂ fl), *P. H. Allen 5763* (holotype: F-2 sheets* [1394346!, 1679106!]; isotype: USJ [9016]).

**Figure 5.**
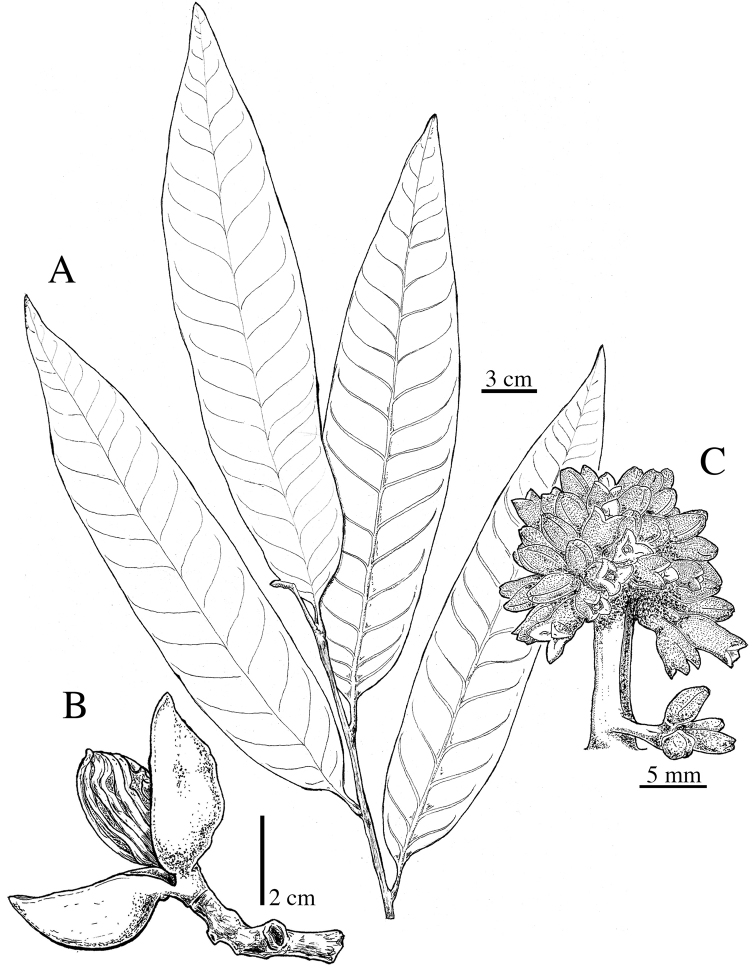
*Virola allenii***A** leaves **B** fruit **C** partial inflorescences. Drawn by Pedro Juarez based on *P. H. Allen 6727* (**A**), *R. Aguilar 2224* (**B**) and *R. Aguilar* (**C**) from a photo, physical specimen not seen.

**Figure 6.**
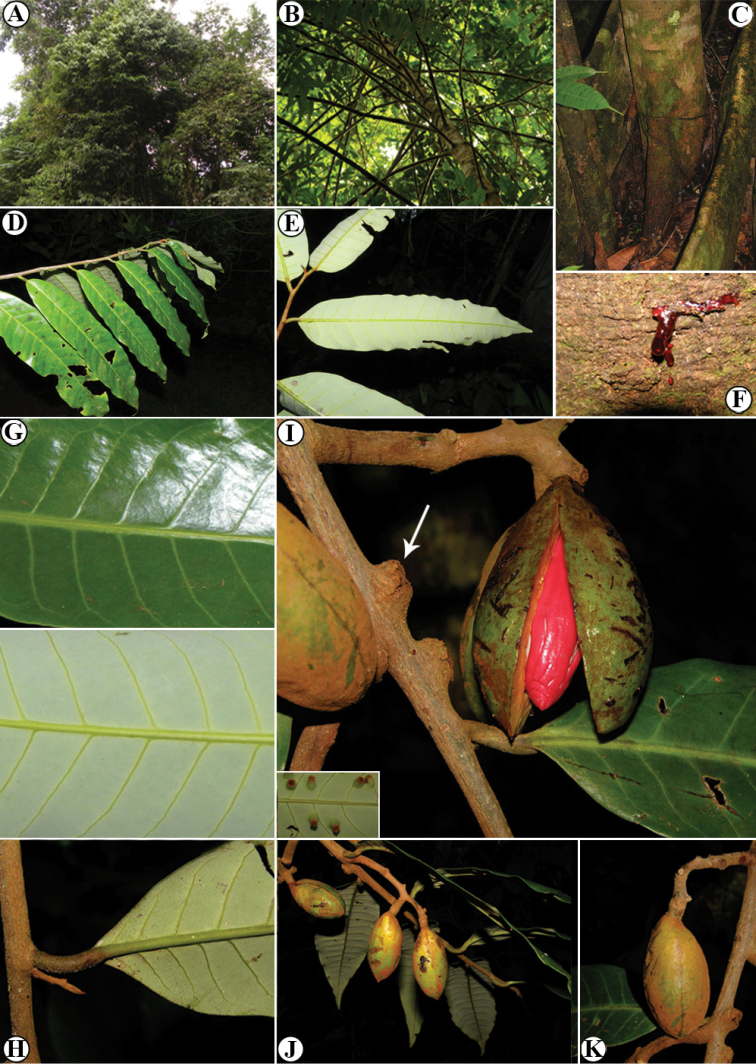
*Virola allenii***A** treetop **B** branching **C** lower trunk and buttress **D** branch with leaves, showing the adaxial surface **E** leaf blades on abaxial surface **F** exudate of the trunk **G** leaf blade surfaces, adaxial (above) and abaxial (below) **H** petiole and leaf base **I** mature fruit, inset left and arrow showing the galls on leaves and branches, respectively **J** fruits **K** fruit close-up. Photos by Reinaldo Aguilar.

**Figure 7.**
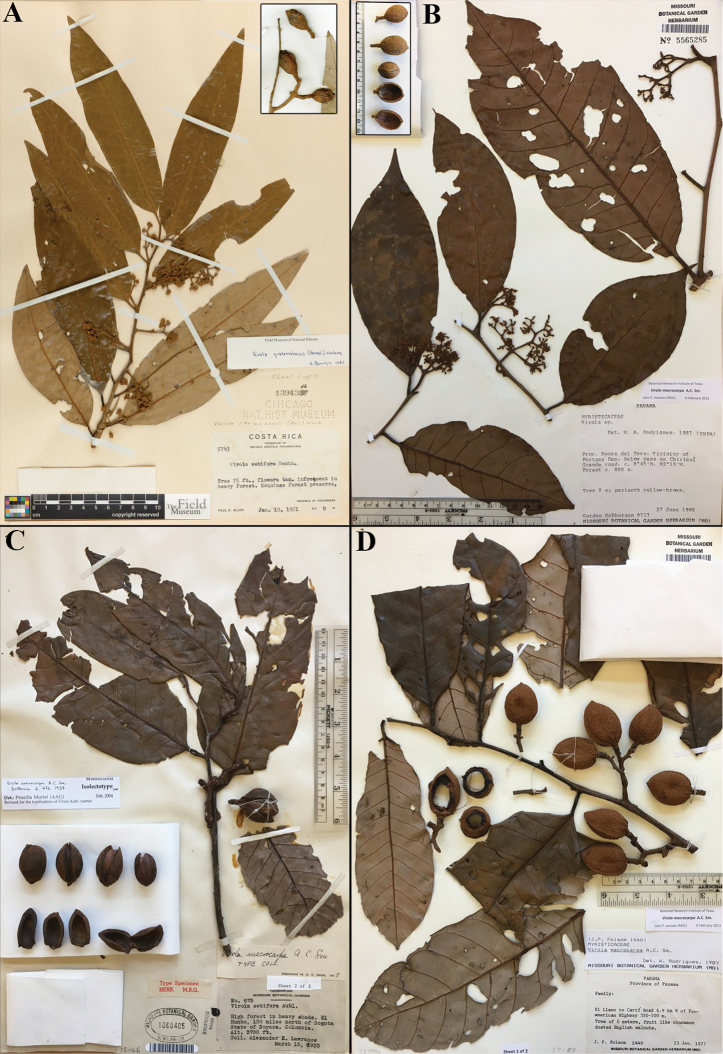
Comparisons of *Virola macrocarpa* with similar species in Mesoamerica **A***V. allenii* (*P. H. Allen 5763*, F; inset fruits, from *P. H. Allen 6727*, GH) **B***V. amistadensis* (*G. McPherson 9717*, MO; inset fruits, from *G. McPherson 9715*, MO) **C***V. macrocarpa* (*A. E. Lawrance 675*, MO) **D***V. otobifolia* (*J. P. Folsom 1440*, MO). Image courtesy of Field Museum (**A**).

#### Description.

***Tree*** 13–30 m × 10.4–50 cm DBH; bark sometimes described as smooth and reddish or dark brown. ***Exudate*** sometimes described as abundant and reddish or watery, but without specifying from where or red in the trunk. ***Twigs*** 0.16–0.22 cm thick, terete to slightly flattened laterally, puberulent, trichomes stellate to irregularly stellate, ferruginous. ***Leaves***: petiole 0.5–1.4 × 0.13–0.24 cm, slightly canaliculate, tomentose, the trichomes stellate to irregularly stellate; ***leaf blades*** 16.2–29.2 × 3.2–7.3 cm, oblong-elliptic or rarely elliptic; adaxial surface of mature leaves olive or light brown (sometimes shining) when dry, glabrous or with scattered stellate trichomes, the surface smooth; abaxial surface pale brown to whitish when dry, densely but inconspicuously pubescent, trichomes stellate, sessile, the central part of the trichome reddish and contrasting in colour with the hyaline branches to reddish-clear, with 4–10 branches, the branches ± 0.01–0.05 mm long, persistent; lateral veins 15–20 per side, 4–5 veins per 5 cm, 1.2–1.8 cm apart, the same colour as the adaxial surface or slightly transparent, on adaxial side flat to sunken, on abaxial side slightly elevated, arcuate-ascending, slightly anastomosing near the margin and without forming a very marked intramarginal vein; tertiary veins barely visible on both sides; midvein adaxially slightly elevated (sometimes flat, distally), abaxially raised, rounded to somewhat triangular, tomentose to glabrate; base acute or obtuse to rounded, not revolute, flat; margin flat; apex acuminate or rarely rounded. ***Staminate inflorescences*** 3.5–5.5 cm long, axillary, axes flattened, tomentose, with trichomes irregularly stellate, ferruginous; peduncle 1.2–1.9 × ca. 0.1 cm long; bracts not seen; terminal fascicles dense, with 15—40+ flowers. ***Staminate flowers*** with the pedicel 0.3–1.2 mm long; receptacle 1.2—2 mm wide; perianth 2–2.8 mm long, infundibuliform, yellow when fresh, connate by 1.1–1.7 (–2.3) mm long, external surface pubescent, with brown trichomes, internal surface glabrous or with few trichomes close to the margin of the lobes; lobes 3 (4), 0.8–1.5 × 0.6–0.9 (–1.2) mm; stamens 3, the filament column 0.5–0.6 mm long, glabrous, straight, thin, sometimes slightly thickened at the base, not constricted at the apex; anthers 0.6–0.9 mm long; apiculus 0.06–0.1 mm long, acute to apiculate, connate. ***Pistillate inflorescences*** and ***pistillate flowers*** not seen. ***Infructescence*** 2.5–7.5 cm long, with 2–13 fruits, peduncle 2–3.5 × ca. 0.47 cm. ***Fruits*** 2.7–3.5 × 1.5–2.5 cm, usually ellipsoid or rarely ovoid, stipitate, densely tomentose, the trichomes dendritic, ferruginous and falling very easily to the touch (as dust), the surface rugose or smooth when dry, the line of dehiscence usually carinate, but not very conspicuous, the base obtuse, the apex acute to obtuse, green or golden and ferruginous by the pubescence when fresh; pericarp 3.2–3.8 mm thick; pedicel 0.4–0.7 cm long; seed ca. 2.5 × 1.3 cm, the testa when dry whitish-greyish, markedly grooved; aril usually described as red when fresh, pale brown when dry, membranaceous, the texture dry and thin, laciniate in narrow bands distally. ***Germination*** epigeal, seedling cryptocotylar, the first pair of leaves (sub)opposite (Ley López and Chacón Madrigal 2017; as *V. macrocarpa*).

#### Distinctive characters.

**Figure 8.**
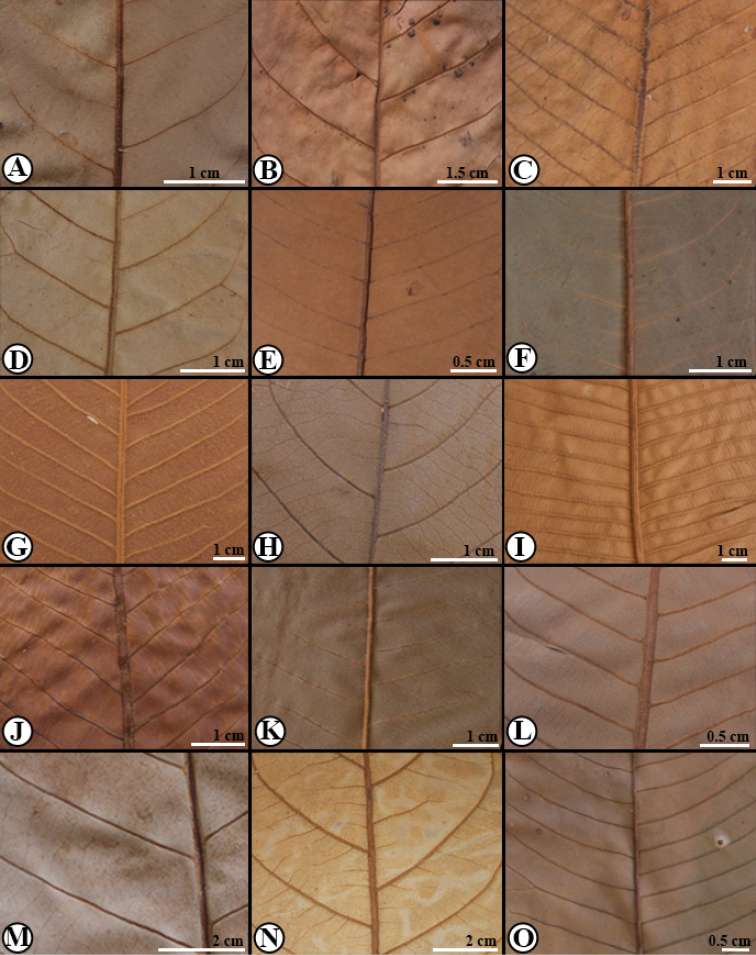
Pattern of the lateral veins in Mesoamerican *Virola***A***Virola allenii* (*K. Thomsen 1284*, NO) **B***V. amistadensis* (*G. McPherson 8703*, MO) **C***V. chrysocarpa* (*B. Hammel et al.16864*, MO) **D***V. elongata* (*M. Correa & R. L. Dressler 1078*, MO) **E***V. fosteri* (*G. de Nevers 7226*, MO) **F***V. guatemalensis* (*G. Ibarra 957*, MO) **G***V. koschnyi* (*J. Miller & J. C. Sandino 1110*, MO) **H***V. laevigata* (*N. Zamora & T. D. Pennington 1583*, MO) **I***V. megacarpa* (*G. de Nevers 5184*, MO) **J***V. montana* (*E. Lépiz & J. F. Morales 284*, MO) **K***V. multiflora* (*J. Manzanares 3561*, MO) **L***V. nobilis* from Barro Colorado (*R. J. Schmalzel 320*, MO) **M***V. otobifolia* (*G. de Nevers et al. 7530*, MO) **N***V. sebifera* (*T. B. Croat 5959*, MO) **O***V. nobilis* from Osa Peninsula (*R. Aguilar 11186*, MO).

*Virola allenii* is recognised by its narrow leaf blades with lateral veins that are well separated (Fig. 8A) with a whitish abaxial surface and covered with stellate, sessile trichomes with the central portion of the trichome reddish and contrasting in colour with the hyaline branches to reddish-clear (Figs 3A, 6F); the staminate flowers with the lobes of the perianth almost glabrous on the inner surface, the column of filaments straight and not constricted at the apex, anthers that are usually longer (0.6–0.9 mm long) than the column of the filaments (0.5–0.6 mm long) and an apiculus that is 0.06–0.1 mm long. It is also distinctive for its large, usually ellipsoid fruits (Figs 4A, 6I–K) with thick pericarp that are green when ripe and covered by ferruginous trichomes that fall very easily to the touch (Fig. 6K inset); and seeds with the testa markedly ribbed.

#### Etymology.

The specific epithet honours the collector of the type specimen, Paul H. Allen (1911–1963), who was probably the first person to collect this species 67 years ago (*P. H. Allen 5763*; 10 Jan 1951). During his five-year residency in Palmar Norte, Puntarenas, Costa Rica (Grayum et al. 2004), Allen made important collections and publications in this region (e.g. The Rain Forests of Golfo Dulce, Allen 1956).

#### Distribution.

**Figure 9.**
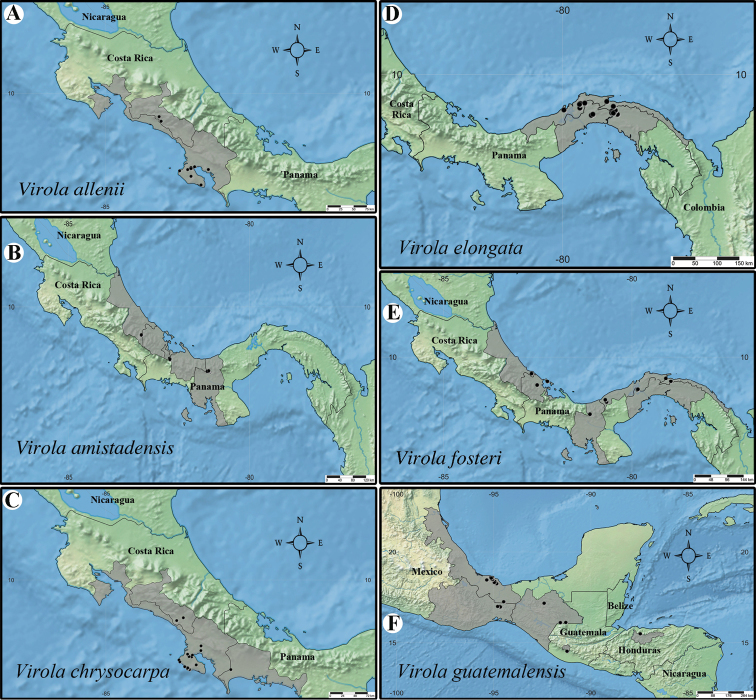
Geographic distribution of *Virola allenii* (**A**), *V. amistadensis* (**B**), *V. chrysocarpa* (**C**), *V. elongata* (**D**), *V. fosteri* (**E**) and *V. guatemalensis* (**F**).

*Virola allenii* is known only from Costa Rica (Puntarenas and San José) (Fig. 9A). It is found on the Pacific slope, at 0–350 (–1350) m elevation.

#### Preliminary conservation status.

*Virola allenii* is Vulnerable following IUCN critera B1a and B2a. Justifying its status, it is known from seven localities and has an EOO of 3,424 km^2^ and an AOO of 40 km^2^. Specimens have been collected regularly since the 1990s during botanical expeditions in the Osa Peninsula of Costa Rica, though only 22 specimens have been verified.

#### Common names.

None recorded.

#### Phenology.

Flowering of *Virola allenii* has been recorded in January, March, April and December. Fruits have been observed in January, August to October. Pistillate flowers were not seen in the studied material.

#### Field characters.

The bark is described as brown and smooth or as peeling in small pieces, sometimes with a strong, spicy scent. Twigs and leaves often have galls (e.g. Fig. 6I). Leaf blades are adaxially lustrous and abaxially whitish. Staminate flowers are yellow and fragrant. Fruits, which are ca. 5 × 3.2 cm when fresh, are green at maturity and covered with brown trichomes that fall very easily when touched and have a pericarp that is ca. 6 mm thick. The aril of mature fruits is red and white in immature fruits. Seeds have a white testa.

#### Discussion.

*Virola allenii* is most similar to *V. macrocarpa*, a species from montane forests at 1100 m elevation in the Andes of Colombia (Boyacá) and this name has been previously applied to the species described here (e.g. Jiménez 2007; Cornejo et al. 2012; Aguilar et al. 2017 onward). It is differentiated from *V. macrocarpa* by the characteristics presented in the diagnosis and in Table 2.

The comparison presented hereafter for *Virola macrocarpa* (Fig. 7C) is strictly based on the protologue (except for the pericarp thickness), from which we have been able to study two physical duplicates deposited at MO (*A. Lawrance 675*, MO-2 sheets!, fr [Fig. 4D]) and the images at A! (st), F! (2-sheets, fr), G! (2-sheets, st.), K! (st), S (fr) and US! (2-sheets, fruits likely in the packet, but not seen). We confirmed all measurements from the protologue on *Lawrance 675* (MO) and found them consistent with the exception of pericarp thickness; while 2–4 mm was stated in the protologue, our measurements ranged from 1.8–3 mm, which we present in Table 2 below. In our estimation, all other observed South American specimens, annotated with this name, represent an amalgamation of different identities (D. Santamaría-Aguilar, in prep.).

**Figure 10.**
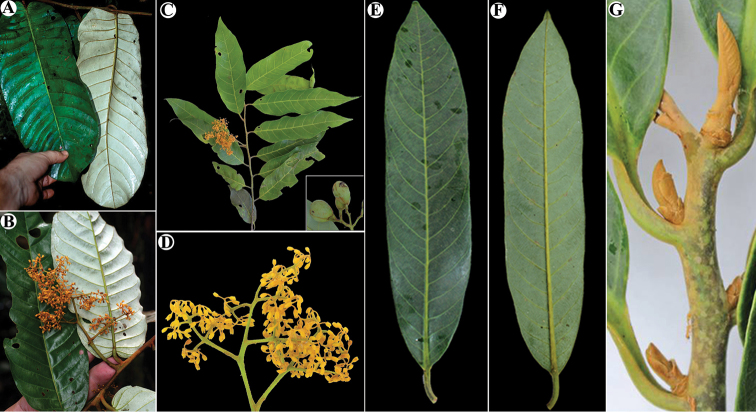
*Virola calophylla***A** branch with leaves, showing both sides **B** branch with inflorescences. *Virola elongata***C** leafy branch with inflorescences, inset fruit **D** inflorescences. *Virola guatemalensis***E, F** leaf blades showing adaxial (**E**) and abaxial (**F**) surface. **G** young twigs and petioles. Photos by Robin Foster (**A, B)**, from https://plantidtools.fieldmuseum.org/en/nlp; Steven Paton (**C, D)**, Rolando Pérez (**C** inset), from https://stricollections.org/portal/index.php and Angela Rojas (**E–G**).

Based on a number of features listed in the diagnosis of *V. alleni*, including colour of the abaxial leaf blade, sessile trichomes, degree of separation of lateral veins and the length of the anther apiculus, *V. allenii* is similar to *V. calophylla* (Fig. 10A, B) and *V. calophylloidea* from South America; the latter was recently included as a synonym of *V. calophylla* (see notes below). Furthermore, *Virola allenii* also shares narrow oblong-elliptic leaf blades (3.2–8 cm broad) and short inflorescences with *V. calophylloidea*. However, it is distinguished from both species by the filament column that is constricted at or towards the apex (vs. not constricted in *V. allenii*) and the tendency towards short anthers (0.4–0.7 mm vs. 0.6–0.9 mm long). Additional differences amongst these three species are presented in the Table 2.

In Mesoamerica, *Virola allenii* can be confused with *V. amistadensis* and *V. otobifolia* (which are formally described as new below). All these species have lateral veins that are well spaced (Fig. 8A, B, M) and an abaxial leaf surface that is usually whitish with sessile stellate trichomes; for differences between these species see Table 3.

Some of the first specimens of this new species were confused with other taxa, though they differ based on their trichomes: *Otoba* (e.g. *L. J. Poveda 887*, CR!) with malpighiaceous trichomes (vs. stellate trichomes), *Virola sebifera* (e.g. *N. Zamora et al. 1440*, CR!) with dendritic to dendritic-stellate and generally pediculate trichomes (vs. stellate and sessile) (Fig. 3A, N); or *V. guatemalensis* (e.g. *P. H*. *Allen 6727*, F, GH) with the central part of the trichome colourless (vs. reddish) and with more compressed lateral veins (0.6–1.1 cm vs. 1.2–1.8 cm of separation between veins) (Figs 3A, 8A, F).

**Table 2.** Comparison of *Virola allenii* with the morphologically most similar species.

Character	*V. allenii*	*V. calophylla*	*V. calophylloidea* ^*^	*V. macrocarpa*
Leaf blade	16.2–29.2 × 3.2–7.3 cm; base acute or obtuse to rounded	(15–) 20–60 × 10–16 cm; base (usually) deeply cordate to truncate (obtuse)	16–33 × 4.5–8 cm; base cordate to rounded or broadly obtuse	20–40 × 7–11 cm; base broadly obtuse^†^
Length of staminate inflorescences	3.5–5.5 cm	6–30 cm	1–4 cm	Unknown
Length of perianth of staminate flowers	2–2.8 mm	1–2.1 mm	1.7–2 mm	Unknown
Filament column	0.5–0.6 mm long; not constricted at apex	0.2–0.6 mm long; constricted at apex	0.7–0.8 mm long; abruptly narrowed at apex	Unknown
Anther length	0.6–0.9 mm	0.4–0.5 mm	0.5–0.6 mm	Unknown
Fruit	2.7–3.5 × 1.5–2.5 cm; base obtuse	2.5–3 × 1.2–2.5 cm; base usually truncate	1.8–2.1 ×0.8–1.1 cm; base obtuse	2.7–3.3 × 2–2.3 cm; base obtuse^†^
Pericarp thickness	3.2–3.8 mm	0.5–5 mm	0.5–0.8 mm	1.8–3 mm

^*^From Smith 1938.

**Table 3.** Comparison of *Virola allenii* with the two other morphologically most similar species in Mesoamerica.

Characters	*V. allenii*	*V. amistadensis*	*V. otobifolia*
Leaf blades	16.2–29.2 × 3.2–7.3 cm	12.3–22.8 (–27) × 4.4–9.5 (–12.5) cm	(14–) 18.2–42.5 × (4.1–) 7.3–14.2 cm
Lateral veins	15–20 per side	9–15 per side	10–16 per side
Length of staminate inflorescence	3.5–5.5 cm	3–7.5 cm	3.5–9.5 cm
Length of perianth of staminate flowers	2–2.8 mm	1.5–2.2 mm	2.5–2.8 mm
Filament column	0.5–0.6 mm; not constricted at apex	0.2–0.4 mm; constricted at the apex	0.9–1 mm; constricted at the apex
Anther length	0.6–0.9 mm	0.6–0.7 mm	0.6–1 mm
Fruit	2.7–3.5 × 1.5–2.5 cm (Fig. 4A)	2.1–3.8 × 1.7–2 cm (Fig. 4B)	(2.7–) 3.5–4.5 × (1.9–) 2.3–2.9 cm (Fig. 4C)
Pericarp thickness	3.2–3.8 mm	1–2 mm	(2.7–) 3–4.7 mm
Seed	ca. 2.5 × 1.3 cm	1.6–2.2 × 1.4–1.6 cm	2.5–2.8 × 1.5–1.7 cm

#### Notes.

The holotype, deposited at Field Museum (F), represents two sheets with hand written annotation (“Sheet 1 of 2,” “Sheet 2 of 2”), which suggests that they represent a multi-sheet specimen of the same plant (ICN Art. 8.3) (Turland et al. 2018).

The specimen *B. Hammel et al. 24041* (CR!; fr) from 1370 m elevation in the Tarrazú region of San José province (Costa Rica) differs from other members of this new species by its more rounded and pubescent fruits and its occurrence at a higher elevation than other specimens. It is included here with some reservation. This specimen is most similar to *E. Alfaro 492* (CR!, LSU!, MO!; ♂ fl), which was also collected in montane forests on the Pacific slope of the Cordillera de Talamanca (1240 m elevation). *E. Alfaro 492* differs from the rest of *V. alleni* in its larger staminate perianth (ca. 3.8–4 mm vs. 2–2.8 mm long) that is fleshy with dense pubescence on the entire adaxial surface (vs. glabrous or with sparse trichomes close to the margin of the lobes in *V. allenii*) and the column of the filaments (ca. 0.5–0.7 mm vs. 0.5–0.6 mm long) that is shorter than the anthers (ca. 1.2 mm vs. 0.6–0.9 mm long). This specimen was also discussed by Jiménez (2007) as *V. macrocarpa*.

The specimens [*P. H*.] *Allen 5763* (type; F, USJ) and [*P. H*.] *Allen 6727* (F, GH), cited as *V. guatemalensis* in The Rain Forests of Golfo Dulce (Allen, 1956), correspond with this new species.

*Virola calophylloidea* has recently been considered synonymous with *V. calophylla* (e.g. Rodrigues 1980; Jaramillo et al. 2004; ter Steege et al. 2019). However, here, it is treated as a morphologically distinct species. This is due to its smaller leaves, more compact staminate and pistillate inflorescences and infructescences, staminate flowers with the filament column longer than the anthers and smaller fruits (see Table 2). Some representative collections of *V. calophylloidea* include:

**Brazil. Acre**: Rio Jurua & Rio Moa, Serra da Moa, 30 Apr 1971 (♂ fl), *P. J. M. Maas et al. P12659* (MO). **Amazonas**: Rio Urubú, 04 Aug 1979 (♂ fl), *C. E. Calderon et al. 2922* (MO); Km 500 on Manaus-Humaitá road, 17 Sep 1980 (fr), *S. R. Lowrie et al. 54* (MO); Km. 133, Manaus-Itacoatiara Road, 11 Sep 1974 (♂ fl), *T. D. Pennington & O. P. Monteiro P22638* (MO); Rio Cuieras, 12 Sep 1973 (♂ fl), *G. T. Prance et al. 17790* (MO); Reserva Experimental Station of INPA, 30 Aug 1974 (♂ fl), *G. T. Prance et al. 21689* (MO); Rio Javari, Rio Curaçá, 8 miles above mouth, 26 Oct 1976 (♀ fl), *G. T. Prance et al. 24133* (MO). **Pará**: Km 133, Madeira-Mamore Railway, 15 Sep 1963 (♂ fl), *B. Maguire et al. 56666* (MO); Itaituba, Km 60 da estrada Itaituba, 16 Nov 1978 (fr), *M. G. da Silva & C. S. Rosário 3775* (MO). **Rondônia**: Basin of Rio Madeira, 23 Nov 1968 (fr), *G. T. Prance et al. 8775* (MO).

#### Specimens examined.

**Costa Rica. Puntarenas**: Golfito, alrededores de la estación Agujas, 300 m elev., 22 May 2000 (st), *L. Acosta et al. 1389* (CR!); Golfito, Piro, 100 m elev., 14 Oct 1991 (st), *R. Aguilar 511* (CR!); Golfito, estación Los Patos, 200 m elev., 02 Sep 1993 (imm fr), *R. Aguilar 2224* (CR-2 sheets!, LSU!, MO!); Osa, Bahía Chal, La Parcela, 150 m elev., 12 Dic 1996 (st), *R. Aguilar 4735* (CR!, MO!); Península de Osa, Rancho Quemado, sendero a Cerro Brujo, 343 m elev., 30 Jul 2013 (st), *R. Aguilar 14519* (CR!); area between Rio Esquinas & Palmar, 60 m elev., 18 Feb 1963 (fr, dupl. in GH), *P. H. Allen 6727* (F!*, GH!*); Osa, Sierpe, 1 km antes de la Villa de Banegas, 55 m elev., 14 Oct 2007 (fr), *E. Chacón et al. 885* (USJ!); Osa, fila Casa Loma, Aguabuena Sur, 07 Oct 1992 (fr), 50–150 m elev., *A. Fernández 410* (CR!, MO!); Osa, Playa Campanario o San Josecito, 1–10 m elev., 29 Dec 1991 (fl bud), *P. Harmon 291* (CR-2 sheets!); Golfito, bosque de los Austriacos, La Gamba, 300 m elev., 09 Jan 1994 (fr), *W. Huber & A. Weissenhofer 136* (CR!, LI*); Golfito, near the Tropenstation La Gamba on the fila, 200 m elev., 27 Jan 2002 (fl), *H. Huber 3012* (LI-2 sheets!*); Golfito, Jiménez, Piro, no elev., 20 Jan 2012 (fr), *J. M. Ley-López 69* (USJ!); Golfito, montaña aledaña al campo de aterrizaje, 07 Jun 1994 (st), *L. J. Poveda 887* (CR!); Osa, 3 km después de la quebrada Banegas, camino a Rancho Quemado, 200 m elev., 10 Jan 2018 (fr), *D. Santamaría & R. Aguilar 9865* (CR!); Península de Osa, Aguabuena, 3.5 km W of Rincón, 350 m elev., 14 Jun 1993 (st), *K. Thomsen 746* (CR!); Península de Osa, Aguabuena, 3 km W of Rincón, 130 m elev., 15 Apr 1993 (♂ fl), *K. Thomsen 857* (CR!, MO!, NY!*); Península de Osa, Aguabuena, 3 km W of Rincón, 130 m elev., 05 May 1993 (st), *K. Thomsen 915* (CR!); Península de Osa, Aguabuena, 3.5 km W of Rincón, 150 m elev., 06 Oct 1994 (fr), *K. Thomsen 1049* (CR!); Península de Osa, Aguabuena, 2.5 km W of Rincón, 150 m elev., 10 Mar 1995 (fl), *K. Thomsen 1283* (NY!*); Península de Osa, Aguabuena, 2.5 km W of Rincón, 150 m elev., 10 Mar 1995 (♂ fl), *K. Thomsen 1284* (NO!, NY!*). **San José**: Tarrazú, del puente de San Marcos 18 km camino hacia Quepos, 1370 m elev., 13 Jan 2006 (fr), *B. Hammel et al. 24041* (CR!); Esquipulas, base del Cerro San Isidro, alrededores del río Naranjo, 400 m elev., 28 Aug 1987 (fr), *N. Zamora et al. 1440* (CR!, MO!).

### 2. Virola amistadensis

Taxon classificationPlantaeMagnolialesMyristicaceae

D.Santam. sp. nov.

71D7988B-A64F-5D00-9A97-2802DD6B6058

urn:lsid:ipni.org:names:77202543-1

Figs 7B , 11

#### Diagnosis.

Similar to *Virola calophylla* in its abaxial leaf surface that is whitish and covered with stellate, sessile trichomes whose centre is reddish-clear to reddish and contrasting in colour with the hyaline branches and the lateral veins that are well-spaced. *Virola amistadensis* can be distinguished from *V. calophylla* by its shorter leaf blades [12.3–22.8 (–27) cm vs. (15–) 20–60 cm long] with a usually acute or sometimes attenuate base (vs. usually deeply cordate to truncate), shorter staminate inflorescence (3–7.5 cm vs. 12–30 cm long) and fruits with an obtuse base (vs. usually truncate).

#### Type.

Panama. Bocas del Toro: Vicinity of Fortuna Dam, below pass on Chiriquí Grande road, ca. 800 m elev., 27 Jun 1986 (♀ fl), *G. McPherson 9717* (holotype: MO! [5565285, MO281258]; isotypes: INPA!* [144165], PMA!* [046113, PMA36179]).

#### Description.

***Tree*** 4–13 m tall, no recorded DBH; bark and exudate not described in herbarium specimens. ***Twigs*** 0.18–0.42 cm thick, inconspicuous but densely strigulose, glabrescent, trichomes irregularly stellate to dendritic, ferruginous to whitish. ***Leaves***: petiole 1.4–2.3 × 0.18–0.23 cm, slightly canaliculate, pubescent, trichomes stellate; ***leaf blades*** 12.3–22.8 (–27) × 4.4–9.5 (–12.5) cm, elliptic to widely elliptic; adaxial surface leaves pale to dark brown (sometimes shining) when dry, glabrous or occasionally with scattered stellate trichomes; abaxial surface usually whitish-greyish when dry, but can be very light to dark brown, densely but inconspicuously pubescent, trichomes stellate, sessile, ferruginous or sometimes with the central part of the trichome reddish, contrasting in colour with the hyaline branches to reddish-clear, with 4–8 branches, the branches ca. 0.02 mm, persistent (the surface with a dense layer of squamiform hyaline structures, especially obvious at high resolution); lateral veins 9–15 per side, 3–5 veins per 5 cm, (1.2–) 1.4–2.5 cm apart, the same colour as the adaxial surface, on adaxial surface flat to slightly elevated or slightly sunken, on abaxial surface raised, free or slightly anastomosing near the margin and without forming a very marked intramarginal vein; tertiary veins barely visible or indistinct on both surfaces; midvein adaxially raised, slightly sunken in a channel, abaxially raised, rounded, tomentose, adpressed pubescent to glabrate; base usually acute or sometimes attenuate, not revolute, flat; margin not revolute; apex acuminate. ***Staminate inflorescences*** 3–7.5 cm long, axillary, axes flattened, pubescent, with trichomes stellate, ferruginous; peduncle 1–2 × 0.09–0.16 cm; bracts not seen; terminal fascicles lax, with 2–11 flowers. ***Staminate flowers*** with the pedicel 0.7–1 mm long; receptacle sometimes ca. 1 mm wide; perianth 1.5–2.2 mm long, infundibuliform, brown when fresh (possibly due to indumentum), connate for ca. 0.8 mm of length, abaxial surface pubescent with brown trichomes, adaxial surface pubescent, especially on the lobes and margins; lobes 3, ca. 1.2 × 0.7 mm; stamens 3 (–6), the filament column 0.2–0.4 mm long, glabrous, straight, conspicuously thickened throughout its length, constricted at the apex; anthers 0.6–0.7 mm long; apiculus 0.1–0.15 mm long, apiculate, connate or slightly separated at the apex. ***Pistillate inflorescences*** 4.3–5 cm, similar to staminate inflorescences; peduncle 1.5–1.8 × 0.18–0.25 cm; bracts not seen; terminal fascicles of 4—10 flowers. ***Pistillate flowers*** in terminal fascicles of 4–10 flowers; with the pedicel 1.5–2 mm long; perianth 3.4–3.8 mm long, infundibuliform, brown-yellow when fresh, connate for 2.2–2.5 mm of length, abaxial surface pubescent, with brown trichomes, adaxial surface pubescent, especially on the lobes and sparsely pubescent basally; lobes 3, 1–1.4 × 1–1.2 mm; gynoecium 1.8–2 × 0.8–1.1 mm, densely pubescent, ovate, sessile; stigmatic lobes 0.4–0.5 mm, erect. ***Infructescence*** 2.7–6.3 cm long, with 1–5 fruits, peduncle 1–2.5 × 0.25–0.5 cm. ***Fruits*** 2.1–3.8 × 1.7–2 cm, ovoid to globose, shortly stipitate, densely tomentose, the trichomes irregularly stellate, ferruginous, persistent, the surface smooth to rugulate when dry, the line of dehiscence slightly carinate, the base obtuse, the apex acute to obtuse, brown to brown-yellow when fresh; pericarp 1–2 mm thick; pedicel 0.4–0.9 cm long; seed 1.6–2.2 × 1.4–1.6 cm, the testa when dry pale brown; aril described as red, pale brown or blackish when dry, membranaceous, dry in texture, thin, laciniate in narrow bands.

#### Distinctive characters.

*Virola amistadensis* is recognised by its elliptical to widely elliptical leaf blades with lateral veins that are well separated (Fig. 8B) and abaxially covered with stellate, sessile trichomes (Fig. 3B) and a dense layer of squamiform structures that are hyaline in colour; the small staminate flowers with a thickened filament column that is markedly constricted at the apex and shorter (0.2–0.4 mm long) than the anthers (0.6–0.7 mm), which are apiculate at the apex; and the ovoid to globose fruits (Fig. 4B) with relatively thin pericarp (1–2 mm thick) (Figs 7B inset, 11D) that are covered by a dense, but inconspicuous layer of ferruginous trichomes. It is also distinctive for being a small tree (4–13 m tall) and its preference for premontane forests.

**Figure 11.**
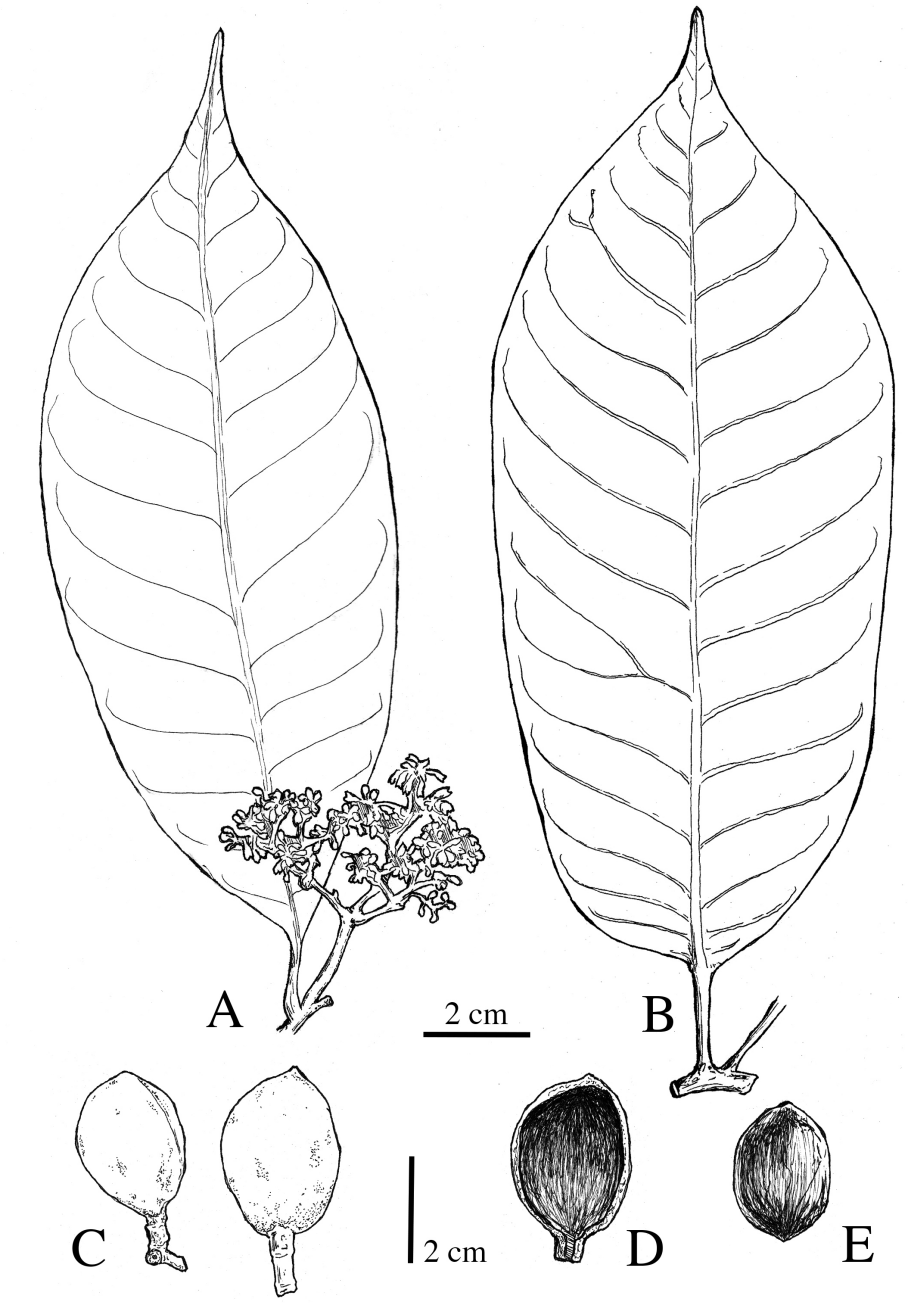
*Virola amistadensis***A** leaf with staminate inflorescences **B** leaf **C** fruits **D** pericarp **E** seed. Drawn by Pedro Juarez, based on *G. McPherson 9717* (**A**) and *G. McPherson 9715* (**B–E**).

#### Etymology.

The specific epithet, *amistadensis*, refers to Parque Internacional La Amistad, a UNESCO World Heritage Site, shared between Costa Rica and Panama where the holotype and some of the paratypes of this species were collected.

#### Distribution.

*Virola amistadensis* is known from Costa Rica (Limón) and Panama (Bocas del Toro and Veraguas; Fig. 9B). It is found on the Caribbean slope at 650–1200 m elevation.

#### Preliminary conservation status.

*Virola amistadensis* is Endangered following IUCN criteria B1a and B2a. This species is known from 4 localities and has an EOO of 2,573 km^2^ and an AOO of only 28 km^2^. Only nine specimens were verified in this study. While it occurs in protected areas, its montane habitat is particularly prone to habitat disturbance.

#### Common names.

None recorded.

#### Phenology.

Flowering of *V. amistadensis* has been recorded in April, June and July and fruiting in January to March, May, June and December.

#### Field characters.

Plants are trees that are 4–13 m tall. Flowers have a yellow-brown perianth and brown fruits.

#### Discussion.

Herbarium specimens of this new species usually have been identified as *Virola calophylla* (Figs 10A, B) or *V. macrocarpa* (Fig. 7C), probably because its leaves are abaxially whitish and covered with stellate, sessile trichomes with a reddish-clear to reddish centre that contrasts in colour with the hyaline branches and lateral veins that are well-spaced. Differences between *V. amistadensis* and *V. calophylla* are enumerated in the diagnosis, while those separating it from *V. macrocarpa* are listed in Table 4.

In Mesoamerica, *Virola amistadensis* is similar to *V. allenii* (Figs 6, 7A) and *V. otobifolia* (Figs 7D, 23A–E), two species from the lowland wet forest of Costa Rica and Panama. Their similarities include the characteristics of the leaf blades mentioned above. Their differences are summarised in Table 3.

**Table 4.** Comparison of *Virola amistadensis* with the morphologically most similar species.

Characters	*V. amistadensis*	*V. macrocarpa*
Leaf blades	12.3–22.8 (–27) × 4.4–9.5 (–12.5) cm; on abaxial side, densely but inconspicuously pubescent	20–40 × 7–11 cm; on abaxial side, inconspicuously puberulent^*^
Lateral veins	9–15 per side, 3–5 veins per 5 cm, (1.2–) 1.4–2.5 cm apart	14–16 per side^*^, 4–5 veins per 5 cm, 0.8–1.5 cm apart
Petiole	0.18–0.23 cm thick	0.3–0.5 cm thick
Fruit size	2.1–3.8 × 1.7–2 cm (Fig. 4B)	2.7–3.3 × 2–2.3 cm^*^ (Fig. 4D)
Infructescence peduncle length	1–2.5 cm	2.5–3 cm
Pedicel length in fruit	0.4–0.9 cm	0.3–0.5 cm^*^
Pericarp	1–2 mm thick, with persistent trichomes	1.8–3 mm thick, with caducous trichomes
Seed size	1.6–2.2 × 1.4–1.6 cm	2.2–2.5 × 1.5–1.7 cm^*^
Tree size	4–13 m	20–30 m^†^

^*^ From Smith 1938.

#### Notes.

Specimens from Veraguas Province (Panama), have smaller leaf blades and lateral veins that are more deeply sunken on the adaxial surface than the specimens from Limón and Bocas del Toro provinces.

#### Specimens examined.

**Costa Rica. Limón**: Parque Internacional La Amistad, subiendo por la fila entre la margen derecha del Río Uren y la Quebrada Crori, Croriña, 650 m elev., 17 Jul 1989 (♂ fl), *A. Chacón 194* (CR, MO!). **Panama. Bocas del Toro**: Along pipeline road in area of Fortuna Dam, 900–950 m elev., 08 Mar 1986 (fr), *G. McPherson 8703* (INPA!*, MO!, PMA!*); vicinity of Fortuna Dam, below pass on Chiriquí Grande road, 800 m elev., 27 Jun 1986 (fr), *G. McPherson 9715* (INPA!*, MO!); along old pipeline road from continental divide, 900 m elev., 27 Dec 1986 (imm fr), *G. McPherson & J. Aranda 10169* (INPA!*, MO!, PMA!*). **Veraguas**: Vicinity of Escuela de Agricultura Alto Piedra, near Santa Fé, 2800–3200 ft [850–975 m] elev., 03 Apr 1980 (♂ fl), *T. Antonio 4011* (INPA!*, MO!); vicinity of Cerro Tute, 850–1000 m elev., 19 Mar 1987 (fr), *G. McPherson 10687* (MO!); near Cerro Tute-Arizona, above Santa Fe and Alto de Piedra, 850–1100 m elev., 05 Feb 1988 (fr), *G. McPherson 12047* (MO!); vicinity of Santa Fe on slopes of Cerro Tute-Arizona above school at Alto Piedra, 900–1100 m elev., 29 Jan 1989 (fr), *G. McPherson 13669* (INPA!*, MO!); Cerro Tute, 1 km beyond Escuela Agrícola Alto Piedra above Santa Fe, 900–1200 m elev., 14 May 1981 (fr), *K. Sytsma & L. Andersson 4653* (MO!).

### 3. Virola chrysocarpa

Taxon classificationPlantaeMagnolialesMyristicaceae

D.Santam. & Aguilar sp. nov.

A0D0CB89-9ED9-55D1-ABE6-3768352D7FDA

urn:lsid:ipni.org:names:77202545-1

Figs 12 , 13 , 14

#### Diagnosis.

Species similar to *Virola koschnyi* due to many characteristics of the leaf, including overall shape, number of lateral veins and stalked trichomes. It differs in leaf blades with pubescent adaxial surfaces that are rough to the touch in herbarium specimens (vs. adaxial surface glabrous to glabrescent and smooth) and abaxial surfaces that are hirsute to hirsutulous (vs. tomentose) with trichomes that have few (3–6 vs. 4–10), but long branches (0.2–0.6 mm vs. 0.1–0.2 mm long), staminate flowers with a longer filament column (1.3–1.5 mm vs. 0.7–0.9 [–1.4)] mm long) and fruits with an acute to apiculate apex (vs. typically obtuse).

#### Type.

Costa Rica. Puntarenas: Golfito, Parque Nacional Corcovado, Estación Sirena, 10 m elev., 06 Feb 1994 (♂ fl), *R. Aguilar 3082* (holotype: CR! [9864]; isotypes: CR! [201389], LSU! [0193694, LSU00199098], MO! [5551151, MO280080], USJ! [60813]).

#### Description.

***Tree*** 15–45 m × 25–50 cm DBH; bark sometimes described as reddish to reddish-brown. ***Exudate*** described as light red but without specifying from which part or red from the trunk. ***Twigs*** 0.18–0.28 cm thick, terete, flattened laterally to slightly angulate, hirsute tomentose, trichomes dendritic, yellowish or very pale brown. ***Leaves***: petiole 1–1.6 (–2) × 0.15–0.28 cm, canaliculated, pubescent, the trichomes dendritic; ***leaf blades*** (17.5–) 24.2–28.8 × 7.6–10 cm, obovate to oblong; adaxial surface of mature leaves olivaceous, brown to greyish when dry, hirsute to hirsutulous, asperous (in new leaves hirsute, the trichomes dendritic-stellate, pediculate, asperous to the touch); abaxial surface similar in colour to the adaxial surface when dry, densely hirsute to hirsutulous, trichomes dendritic to dendritic-stellate, yellowish to pale brown, pediculate, with 3–6 branches, the branches 0.2–0.6 mm long, persistent; lateral veins 28–32 per side, with 5–7 (–11) veins per 5 cm, (0.5–) 0.7–1.3 cm apart, the same colour as the adaxial surface or sometimes contrasting in colour, on adaxial surface flat to slightly sunken, on abaxial surface conspicuous and raised, straight to slightly arcuate, anastomosing near the margin, forming an intramarginal vein; tertiary veins usually inconspicuous adaxially, conspicuous abaxially; midvein adaxially flat, pubescent, abaxially raised, rounded, pubescent; base usually markedly cordate, not revolute, flat; margin flat, sometimes ciliolate; apex acuminate. ***Staminate inflorescences*** 4–8.5 cm long, usually at nodes lacking leaves or, on few occasions, in the axis of leaves, axes slightly flattened, densely pubescent, the trichomes dendritic, yellowish to pale brown; peduncle 1.5–4 × 0.13–0.19 (–0.3) cm; bracts 0.5–0.8 × 0.3–0.5 cm, pubescent on both sides, caducous; terminal fascicles dense, with 15–30 + flowers. ***Staminate flowers*** with the pedicel 2.8–3.5 mm long; receptacle 2–4 mm wide; perianth 2–3 mm long, subglobose to rhomboid, yellow when fresh, connate for 0.6–1.5 mm of length, abaxial surface pubescent, with pale brown, yellowish or golden trichomes, adaxial surface with few scattered trichomes, especially on the lobes; lobes 3, 1.5–2.3 × 0.6–1.5 mm; stamens 3, the filament column 1.3–1.5 mm long, thin, not constricted at the apex; anthers 0.6 mm long; apiculus apparently absent, the apex obtuse; pollen 28 µm, with bilateral symmetry, boat shaped to elliptic grain, exine reticulate, exine structure tectate-perforate (based on Lambright 1981; *Skutch 4260*, US). ***Pistillate inflorescences*** and flowers not seen. ***Infructescence*** 3.2–7.5 cm long, 1–2 fruits (sometimes 4 in an immature infrutescence), peduncle 1.2–5 × 0.18–0.27 cm. ***Fruits*** 2.4–2.9 × 1.7–1.8 cm, ellipsoid, sessile, densely tomentose to glabrate, the trichomes dendritic, brown to brown-reddish, the surface smooth to rugulose, the line of dehiscence smooth, canaliculate, to slightly carinate, the base rounded, the apex acute to apiculate, yellow, orange or ferruginous (possibly by the indumentum) when fresh; pericarp 1.8–2.5 mm thick; pedicel 0.5–0.8 cm long; seed ca. 1.7–2.1 × 1.3–1.4 cm, the testa pale brown to blackish when dry, slightly grooved to almost smooth; aril usually described as red or pink when fresh, brown or yellowish when dry, oily, thick, laciniate in narrow bands or wide distally. Germination epigeal, seedling cryptocotylar (Ley López and Chacón Madrigal 2017; as *V. koschnyi*).

**Figure 12.**
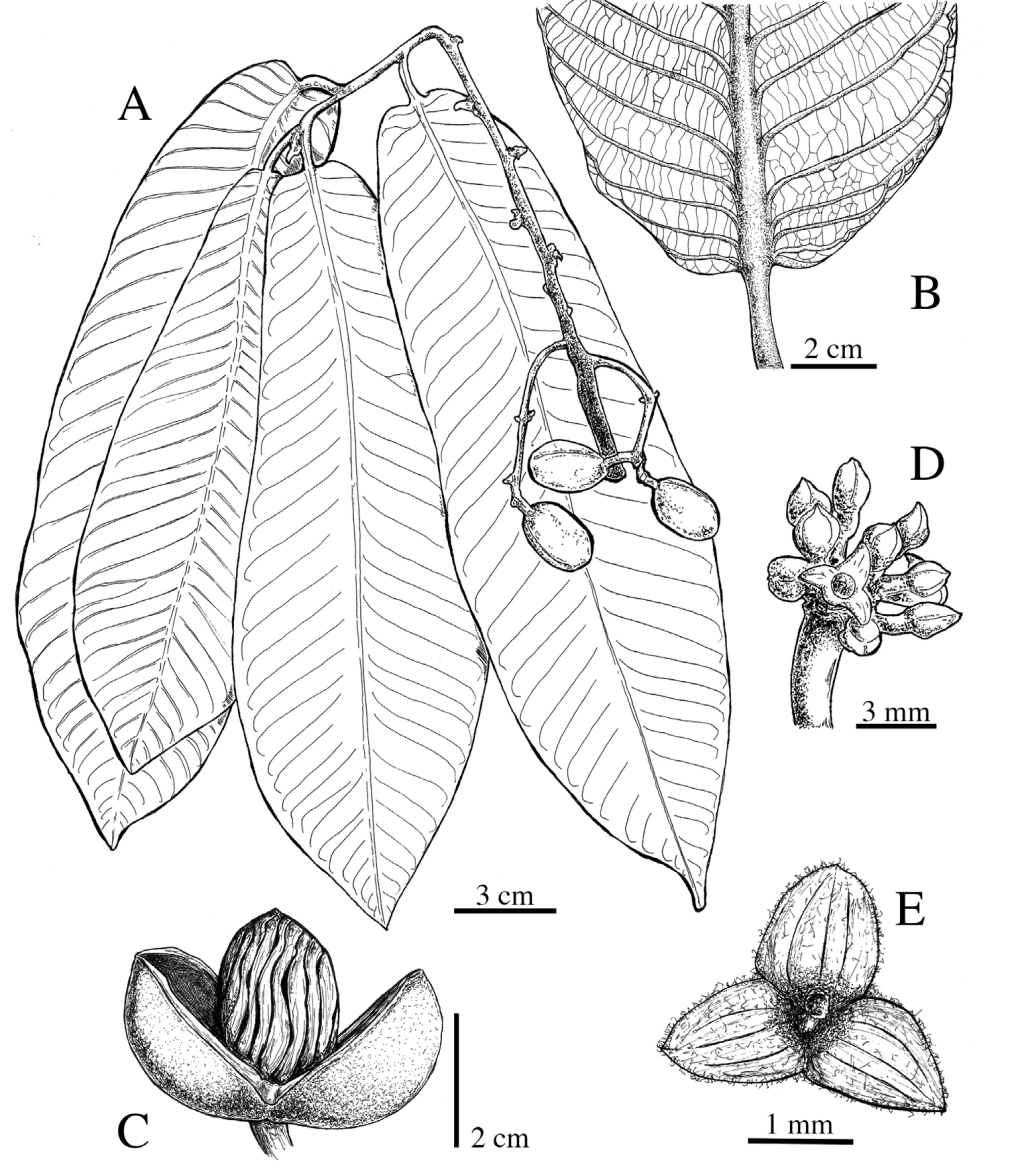
*Virola chrysocarpa***A** branch with leaves and fruits **B** base of young leaf **C** fruit **D** partial staminate inflorescences **E** staminate flower. Drawn by Pedro Juarez, based on *R. Aguilar 11190* (**A–C**) and *R. Aguilar 11569* (**D, E**).

#### Distinctive characters.

**Figure 13.**
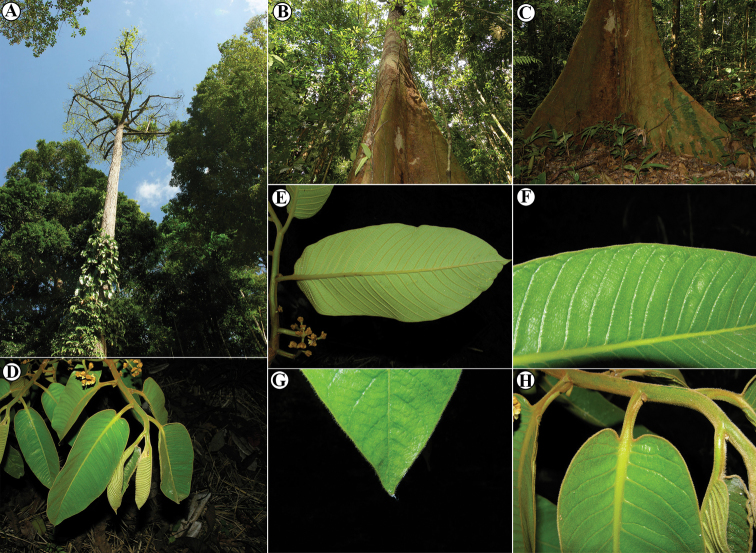
Vegetative characteristics of *Virola chrysocarpa***A** tree showing deciduous nature of the species **B** trunk **C** buttress **D** branch showing new leaves **E** leaf blades on abaxial surface **F** leaf blades on abaxial surface, showing margin detail and venation **G** leaf apex **H** new branch and leaf base. Photos by Reinaldo Aguilar.

**Figure 14.**
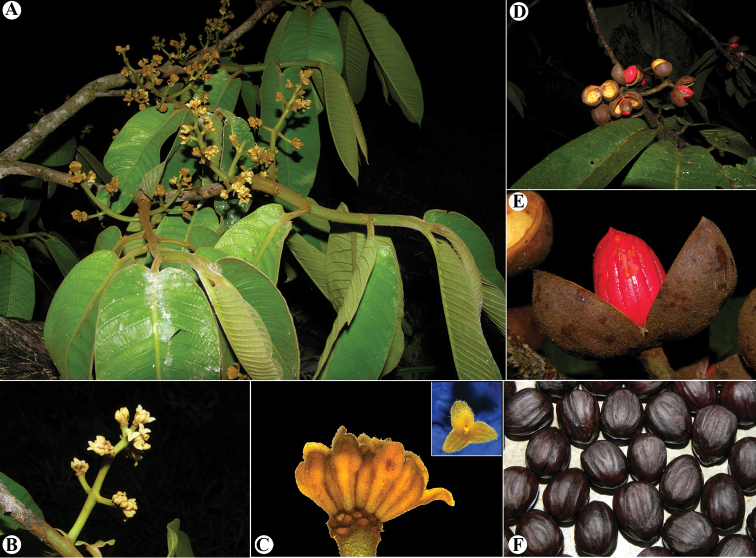
Fertile characteristics of *Virola chrysocarpa***A** branch with staminate inflorescences **B** staminate inflorescences **C** staminate flower buds and open flower **D** Infructescence **E** open fruit **F** seeds. Photos by Reinaldo Aguilar.

*Virola chrysocarpa* is distinguishable for its leaf blades with pubescent adaxial surfaces that are rough to the touch in mature leaves (at least in herbarium specimens) and abaxial surfaces that are hirsute to hirsutulous with trichomes with long branches (0.2–0.6 mm long) (Fig. 3C), numerous lateral veins (28–32 per side), tertiary veins that are usually conspicuous on both surfaces (Figs 3C, 8C) and with a base that is usually markedly cordate; staminate inflorescences that are little-branched (Fig. 14D) with flowers with filament columns that are much longer (1.3–1.5 mm) than the anthers (0.6 mm); and fruits that are acute to apiculate at the apex (Fig. 14E, F, H). Additionally, as far as we are aware, this is the only Mesoamerica species that is completely deciduous (i.e. all leaves fall off the tree) (Fig. 13A).

#### Etymology.

The specific epithet, *chrysocarpa*, is derived from the Greek *chryso* (gold) and *carpo* (fruit). This is in reference to its common name, “fruta dorada” (golden fruit), which is used by locals of the Osa Peninsula, Costa Rica, where this species is frequent.

#### Distribution.

*Virola chrysocarpa* is known from Costa Rica (Puntarenas and San José) and Panama (Chiriquí) (Fig. 9C). It is found in the Pacific slope at 0–700 m in elevation.

#### Preliminary conservation status.

Possible Near Threatened: This species has a small estimated AOO (60 km^2^), though a relatively large estimated EOO of 5,334 km^2^. Its eighteen known specimens represent eleven localities. This limited number of specimens warrants a Possible NT status, though additional collection efforts may demonstrate the lack of conservation threat for this poorly known species.

#### Common names.

Costa Rica: fruta dorada. Panama: bogamani.

#### Phenology.

Herbarium specimens of flowering *Virola chrysocarpa* have been collected in December to March and fruiting specimens from March to June. Herbarium specimens with pistillate flowers were not observed. In the Osa Peninsula, leaves fall completely during the dry season, which occurs in November to February (Allen 1956; Quesada Quesada et al. 1997; and R. Aguilar pers. obs., 2015, 2017, 2018, 2019).

A study of vegetative, flowering and fruiting phenology has been published by Lobo et al. (2008; as *V. koschnyi)* in the Osa Peninsula and Golfo Dulce, Costa Rica. In this study, flowering was documented in January and February, when the canopy was deciduous. Fruiting occurred in the rainy season from June to August.

#### Field characters.

Plants are large trees with boles that are straight and do not begin to branch until they reach a great height, with buttresses, up to 1.6–2.5 m tall. Bark is sometimes described as finely fissured. The new leaves are lime green in colour. Twigs, petioles and leaf blades on both surfaces (especially the youngest ones) are covered with golden, brown-reddish to rusty-red trichomes. Flowers have yellow or yellow-cream perianth and anthers. Mature fruits are yellow, orange or ferruginous (possibly due to their indumentum). Seeds are brown or blackish and covered with a red to scarlet aril.

#### Discussion.

*Virola chrysocarpa* resembles a morphological group of species from South America that includes *V. caducifolia* W. A. Rodrigues, *V. decorticans* Ducke, *V. guggenheimii* W. A. Rodrigues, *V. multicostata* Ducke, *V. multinervia* Ducke, *V. polyneura* W. A. Rodrigues and *V. rugulosa* (Spruce) Warb. These species are characterised by having leaves that are evidently pubescent, some with dendritic to irregularly dendritic pediculate trichomes on the abaxial surface and leaf blades with numerous, conspicuous and comparatively dense lateral veins; staminate flowers with anthers that are subequal to or shorter than the filament column; and fruits with thick pericarp. Additionally, these species tend to be large, sometimes deciduous trees with cordate leaf bases and staminate flowers with the anthers that are obtuse at the apex. Table 5 presents the differences between these species and *V. chrysocarpa*.

**Table 5.** Comparison of *Virola chrysocarpa* with the most morphologically similar species.

Character	*V. chrysocarpa*	*V. caducifolia* ^‡^	*V. decorticans* ^‡^	*V. koschnyi*	*V. guggenheimii* ^‡^	*V. multicostata* ^‡^	*V. multinervia* ^‡^	*V. polyneura* ^‡^	*V. rugulosa* ^‡^
Petiole length	1–1.6 (–2) cm	0.5–0.35 cm	0.7–2 cm	0.5–1.5 cm	0.5–1 (–2) cm	0.2–0.4 cm	0.4–1.5 cm	1–2.5 cm	0.8–1.1 cm^†^
Leaf size	(17.5–) 24.2–28.8 × 7.6–10 cm	10–42 × 3.5–12.5 cm	25–60 × 11–21 cm	14.1–29.9 × 4.2–8.7 cm	5–22 (–25.5) × 2–6.5 (–10) cm	20–28 × 4–10 cm	25–45 × 8–16 cm	5.5–11 × 4–8.5 cm	20–27 × 7–9.5 cm
Adaxial pubescence	Pubescent	Glabrous	Pubescent (pilose)	Glabrous	Pubescent (sparsely strigulose)	Glabrous or pubescent on the veins^†^	Glabrous	Glabrous (pubescent on the midvein)	Glabrous (pubescent on the midvein)
Trichomes on abaxial side	Pediculate	Sessile	Pediculate	Pediculate	Pediculate	Pediculate^†^	Pediculate	Pediculate	Pediculate
No. of lateral veins	28–32	48–60 (–69)	45–60	(16–) 20–35	24–58	50–60	40–60	30–50	23–27
Staminate infls. length	4–8.5 cm	18 cm	22 cm	5–11 cm	14 cm	ca. 15 cm	15–20 cm	5 cm	25 cm
Staminate perianth length	2–3 mm	1–1.4 mm	1.5–1.8 mm	2–2.5 mm	1–1.5 mm	ca. 1 mm	1.2–1.5 mm	None recorded	1.3–1.5 mm^†^
Filament column length	1.3–1.5 mm	0.7–0.9 mm	0.3–0.4 mm	0.7–0.9 (–1.4) mm	0.3–0.4 mm	None recorded	ca. 0.6 mm^§^	None recorded	Not described
Anther length	0.6 mm	0.4 mm	0.5–0.6 mm	0.5–0.7 (–1) mm	0.4–0.5 mm	None recorded	ca. 0.5 mm^§^	None recorded	Not described
Fruit size	2.4–2.9 × 1.7–1.8 cm	2.5–3 × 1.3–2.5 cm	2.7–3.5 × 1.7–2.2 cm	1.9–3.1 × 1.5–1.9 cm	2–2.8 × 1.5–2 cm	2–3.5 × 1.8–2.5 cm	2–3 × 1.5–2.5 cm	2–2.3 × 1.5–1.8 cm	1.5–2 × 1.5–1.7 cm
Pericarp thickness	1.8–2.5 mm	2–3 mm	Not described	1.2–3.1 mm	2–4 mm	2–5 mm	1.5–4 mm	1–2 mm	3 mm
Leaves phenology	Deciduous	Deciduous	Evergreen	Evergreen	Evergreen	Deciduous	Deciduous	Evergreen	Evergreen

^‡^From Rodrigues 1980, except where it is specified otherwise that it comes from ^†^Smith 1938 and ^§^Jaramillo et al. 2004.

In Mesoamerica, *Virola chrysocarpa* resembles and has been confused with, *V. koschnyi* (e.g. Allen 1956; Quesada Quesada et al. 1997; Jiménez 2007; Aguilar et al. 2017 onward) (Figs 3G, 4F, 17), from which it differs by the characteristics included in the diagnosis. Additionally, *V. chrysocarpa* is a deciduous (vs. evergreen) species of the Pacific slope (vs. Caribbean slope). In the region, *Virola chrysocarpa* can also be confused with *V. megacarpa* from Panama for its leaf blades with dense lateral veins and a prominent marginal vein, as well as pediculate trichomes on the abaxial leaf surface; however, *V. megacarpa* has more lateral veins [(32–) 40–50 vs. 28–32 per side], the fruits are larger (4–5.7 × 2–2.9 cm vs. 2.4–2.9 × 1.7–1.8 cm) and with an acuminate to rostrate apex (vs. apex acute to apiculate) (Fig. 4E, P) and thick pericarp (3–6 mm vs. 1.8–2.5 mm).

#### Notes.

The illustration presented in *Manual de Plantas de Costa Rica* (Jiménez 2007) as *V. koschnyi* is a mix of these two species. The branch with leaves and inflorescences (and, most likely, the trichomes) are based on material that represents *V. chrysocarpa* (*R. Aguilar 3082, 3125*), while the other parts of this illustration (staminate flowers and fruits) represent *V. koschnyi* and are based on *F. Araya 197* (fl), and *U. Chavarría 1918* (fr) (B. Hammel pers. comm., Feb 2019).

#### Specimens examined.

**Costa Rica. Puntarenas**: Osa, Parque Nacional Corcovado, Estación San Pedrillo, 10–100 m elev., 19 Feb 1994 (♂ fl), *R. Aguilar 3125* (CR-2 sheets!, MO!); Osa, Reserva Forestal Golfo Dulce, Mogos, a 20 km. de Chacarita, 17 Apr 2008 (fr), *R. Aguilar 11190* (NY!*, USJ!); Rincón, Banegas centro del pueblo, 49 m elev., 15 Dec 2008 (♂ fl), *R. Aguilar 11569* (MO!, NY n.v., PMA!*); forest below Esquinas Experiment Station Residence, area between Río Esquinas and Palmar Sur de Osa, 100 ft [30 m] elev., 30 May 1950 (fr), *P. H. Allen 5554* (CR-2 sheets!, MEXU!*, MO-2 sheets! [photo & dried specimen], PMA!*); Golfito, Estación Agujas, 300 m elev., 18 Feb 1998 (♂ fl), *A. Azofeifa 683* (CR-2 sheets!, MO!); Osa Península, Rancho Quemado, ca. 15 km W of Rincón, 200–400 m elev., 28 May 1988 (fr), *B. Hammel et al. 16864* (CR!, INPA!*, MO!, PMA!*); Rancho Quemado, a lo largo de Río Riyito en la pura entrada al valle, 200 m elev., 31 Mar 1991 (imm fr), *B. Hammel et al. 18186* (CR!, MEXU!*, MO!, USJ!); Parque Nacional Corcovado, Pavo Forest, 0–150 m elev., 16 Jun 1988 (fr), *C. Kernan & P. Phillips 582* (CR!, INPA!*, MO!); Parque Nacional Corcovado, Ollas trail, 0–100 m elev., 09 Jan 1989 (fl bud), *C. Kernan et al. 876* (CR!, MEXU!*, MO!, USJ!); Parque Nacional Corcovado, Llorona Forest, 0 m elev., 16 Jan 1989 (♂ fl), *C. Kernan & P. Phillips 913* (CR!, INPA!*, MO!, USJ!); Parque Nacional Corcovado, Sirena, 1–20 m elev., 15 Jun 1990 (fr), *G. Maass 34* (CR!, MO!); Golfito, Parque Nacional Corcovado, sendero Las Ollas, 100–150 m elev., 21 Mar 1995 (fr), *J. F. Morales 3690* (CR!, LSU!, MO); Aguabuena, 3 km W of Rincón, 120 m elev., 06 May 1993 (fr), *K. Thomsen 371* (CR-2 sheets!). **San José**: [Pérez Zeledón], Vicinity of El General, 700 m elev., n.d., Feb 1939 (♂ fl), *A. F. Skutch 4241* (MO!); [Pérez Zeledón], Vicinity of El General, 740 m elev., n.d. Mar 1939 (♂ fl), *A. F. Skutch 4260* (MO!, US n.v.); Tarrazú, San Lorenzo, camino entre cerro Pito y cerro Toro, 600–700 m elev., 26 May 1998 (fr), *O. Valverde 970* (CR!, MO!, USJ!). **PANAMA. Chiriquí**: Progreso, no elev., Jul–Aug 1927 (fr, at GH, NY), *G. P. Cooper & G. M. Slater 175* (F!*, GH!*, NY!*).

### 4. Virola elongata

Taxon classificationPlantaeMagnolialesMyristicaceae

(Benth.) Warb.

051616A2-0EFF-5C82-80C7-375A645056AA

Fig. 10C–D

Virola elongata (Benth.) Warb. Nova Acta Acad. Caes. Leop.-Carol. German. Nat. Cur. 68: 178. 1897.
Myristica elongata Benth. Hooker’s J. Bot. Kew Gard. Misc. 5: 5. 1853. Type. Brazil. “Brasilia prope Borba”, Aug. 1828, [L.] *Riedel 116* or *s.n.* (holotype: K; isotypes: B destroyed, BM, C, G, LE, P, S; fide Jaramillo et al. 2004).

#### Distinctive characters.

*Virola elongata* it is recognized by its relatively small leaf blades [10.8–18.5 × 2.7–3.7 (–5.1) cm] with a sparsely pubescent abaxial surface with stellate to dendritic-stellate trichomes that are usually sessile (Fig. 3D) and few lateral veins (9–14 per side) that are well separated [(0.8–) 1–1.7 cm apart] (Fig. 8D); staminate inflorescences with thin axes and flowers with short filament columns (0.3–0.4 mm long), anthers that are apiculate at the apex (apicula 0.1–0.2 mm long) and more than twice the length of the column (0.6–0.9 mm long); and its small fruits (1.6–1.9 × 0.9–1.1 cm) (Fig. 4L) that are green when ripe (drying light brown in herbarium specimens with the surface blistering to rough), inconspicuously to sparsely pubescent (densely pubescent when young) with trichomes that are dendritic and brown to ferruginous in colour (when present), pericarp that is 0.5–0.7 mm thick and an aril that is thin and laciniate in narrow bands.

#### Distribution.

In Mesoamerica, *Virola elongata* is only known in Panama (Colón, Panamá and San Blas) (Fig. 9D). It has been recorded from between 50–450 m elevation.

#### Common names.

None recorded in Mesoamerica.

#### Phenology.

Specimens of *Virola elongata* with flowers were collected in July, October and September and with fruits in January, February, April, July, August and October. Collections with pistillate flowers were not seen.

#### Field characters.

Plants are trees between (3–) 7–15 m tall and 0.7–12 cm DBH. Bark cuts are sometimes aromatic. Leaf blades are light green or whitish abaxially. Flowers have a yellow or yellow-orange perianth.

#### Selected specimens seen.

**Panama. Colón**: Camino a la zona maderera de Santa Rita, no elev., 03 Oct 1968 (♂ fl), *M. D. Correa & R. L. Dressler 1078* (MO!); Santa Rita, East ridge, no elev., 23 Jan 1968 (fr), *J. D. Dwyer & Correa 8420* (MO!); near Rio Boqueron, no elev., 11 Oct 1974 (♂ fl), *S. Mori & J. Kallunki 2423* (MO!); Santa Rita Ridge, 1000–1200 ft [305–365 m] elev., 25 Sep 1985 (♂ fl), *K. J. Sytsma 1346* (MO!). **Panamá**: Una milla después del Lago Goofy, no elev., 04 Jan 1968 (imm fr), *M. D. Correa & R. L. Dressler 585* (MO!); El Llano-Carti road, 350 m elev., 16 Jul 1987 (fl bud), *G. McPherson 11275* (MO!); Cerro Azul, [600 m elev.], 06 Aug 1961 (fl bud, imm fr), *J. D. Dwyer 1383* (MO!); Canal Zone, between Chilibre and Madden Dam, no elev. and date (fr), *J. D. Dwyer 8420A* (MO!); vicinity of El Llano, no elev., 07–08 Sep 1962 (♂ fl), *J. A. Duke 5508* (MO!); along Rio Terable, [50 m elev.], 14–15 Sep 1962 (♂ fl), *J. A. Duke 5667* (MO!); West of El Llano, [20–50 m elev.], 03 Oct 1972 (imm fr, ♂ fl), *E. L. Tyson 6866, 6867* (MO!). **San Blas**: San Blas–Panama border, 300 m elev., 01 Feb 1989 (fr), *G. McPherson 13674* (MO!); Río Cangandi, 100 m elev., 17 Feb 1985 (fr), *G. de Nevers 4891* (MO!).

### 5. Virola fosteri

Taxon classificationPlantaeMagnolialesMyristicaceae

D.Santam. sp. nov.

5D1C8010-D8B4-501F-81CA-C3DE1AF0229A

urn:lsid:ipni.org:names:77202547-1

Figs 15 , 16

#### Diagnosis.

Species resembling *Virola multiflora* due its small leaf blades and fruits, similar leaf shape and inconspicuous stellate, sessile trichomes on the abaxial leaf surface. Both species also occur on the Caribbean slope of Mesoamerica. They differ in the shape of the leaf base (revolute in *V. fosteri* vs. not revolute in *V. multiflora*), the length of the filament column (0.9–1.3 mm vs. 0.7–1 mm long) and anthers (0.6–0.9 mm vs. 0.3–0.6 mm long) and thickness of the pericarp (1.5–2.5 mm vs. 0.7–1 mm thick).

#### Type.

Panama. Bocas del Toro: Isla Colón, Aprox. a 8 km al NE de los laboratorios del Instituto Smithsonian de Investigaciones Tropicales, Big Creek, 5 m elev., 23 Apr 2009 (♂ fl), *C. Galdames, M. Stapf, K. Toribio & Arsenio 6422* (holotype: PMA!* [094201, PMA92162]; isotypes: MO! [6421737, MO-2504180], SCZ!* [17752, SCZ17684]).

#### Description.

***Tree*** (15–) 20–35 m × 35–60 cm DBH; bark brown or reddish. ***Exudate*** described as watery-reddish possibly from the bark, damage to any part of the plant causes the flow of a watery exudate that turns reddish moments later. ***Twigs*** 0.12–0.24 cm thick, terete to slightly angulate, puberulent, trichomes stellate, yellowish to pale brown. ***Leaves***: petiole 0.4–0.7 (–1) × 0.07–0.12 cm, canaliculate, densely tomentose to sparsely pubescent, the trichomes stellate; leaf blades 7.8–12 × 1.4–2.7 cm, narrowly elliptical or oblong to oblanceolate; adaxial surface dark brown, light brown or blackish when dry, glabrous, the surface smooth; abaxial surface pale brown to reddish-brown when dry, puberulent, trichomes stellate, sessile, yellowish to pale brown, with 4–8 branches, each branch ± 0.03–0.05 mm long, persistent; lateral veins 16–24 per side, 10–15 veins per 5 cm, 0.2–0.5 (–0.7) cm apart, the same colour as the adaxial surface, on adaxial surface sunken, on abaxial surface flat to slightly elevated, arcuate-ascending, slightly anastomosing near the margin and not forming a marked intramarginal vein; tertiary veins adaxially almost indistinct to slightly sunken, abaxially almost indistinct; midvein adaxially canaliculate, glabrous, abaxially raised, laterally compressed and sometimes resembling a cutting edge, tomentose to sparsely pubescent; base attenuate, revolute; margin revolute (especially near the base) or flat; apex acute to acuminate. ***Staminate inflorescences*** 2.5–5.3 cm long, axillary, usually in the axil of terminal leaves, axes flattened to irregularly angled, tomentose, with trichomes stellate, yellowish to pale brown; peduncle 0.9–17 × 0.13–0.25 cm; bracts 2–5 × ca. 2.5 mm, tomentose on both surfaces, the indumentum more clustered on the external side, caducous; terminal fascicles dense, with 5–15+ flowers. ***Staminate flower*** with the pedicel 1–2 mm long; receptacle 1.5–2.3 mm wide; perianth 2–2.5 (–3) mm long, subglobose, yellow, orange or yellow-orange when fresh, connate for 0.5–0.8 mm of length, abaxial pubescent, with golden to yellowish trichomes, adaxial surface glabrous somewhat pubescent near the lobes; lobes 3, 1.5–2.6 × (0.6–) 1.3–1.8 mm; stamens 3 (–6), the filament column 0.9–1.3 mm long, glabrous, straight or rarely thickened near the base in some flowers (*McPherson 20148*), thin, not constricted at the apex; anthers 0.6–0.9 mm long; apiculus small enough to as appear absent, acute to obtuse. ***Pistillate inflorescences*** 1.3–3.9 cm long, axillary, with trichomes on the axes similar to those of the staminate inflorescences; peduncle 0.7–2.2 × 0.08–0.14 cm; bracts not seen; terminal fascicles of 4–7 flowers. ***Pistillate flowers*** with the pedicel 1.5–2.5 mm long; perianth 2–3 mm long, subglobose, yellow when fresh, connate by 0.6–0.8 mm long, abaxial surface pubescent with golden to yellowish trichomes, adaxial surface sparsely pubescent, the indumentum on the lobes; lobes 3, 1.2–1.5 (–2.5) × 0.7–2.1 mm; gynoecium 1.6–2.4 × 1.1–1.4 mm, densely pubescent, globose to subglobose, stipitate; stigmatic lobes ca. 0.6 mm, erect. ***Infructescence*** 2.5–3 cm long, with 1–3 fruits, peduncle 1.5–1.7 × 0.15–0.38 cm. ***Fruits*** 1.5–2.3 × 1.2–1.8 cm, ovoid, sessile or very shortly stipitate, tomentose, the trichomes stellate, reddish-brown, the surface rugose when dry, the line of dehiscence canaliculate or smooth, the base obtuse to rounded, the apex obtuse, yellow, orange or golden brown when fresh; pericarp 1.5–2.5 mm thick; pedicel 0.4–0.5 cm long; seed ca. 1.6 × 0.9 cm, the testa pale brown when dry, very slightly grooved; aril usually described as red when fresh, reddish-brown when dry, coriaceous, oily, somewhat thick, laciniate in narrow bands. Germination epigeal, seedling cryptocotylar, epicotyl hairy, moderately dense, stellate and sessile (Garwood 2009; as *V. multiflora*).

**Figure 15.**
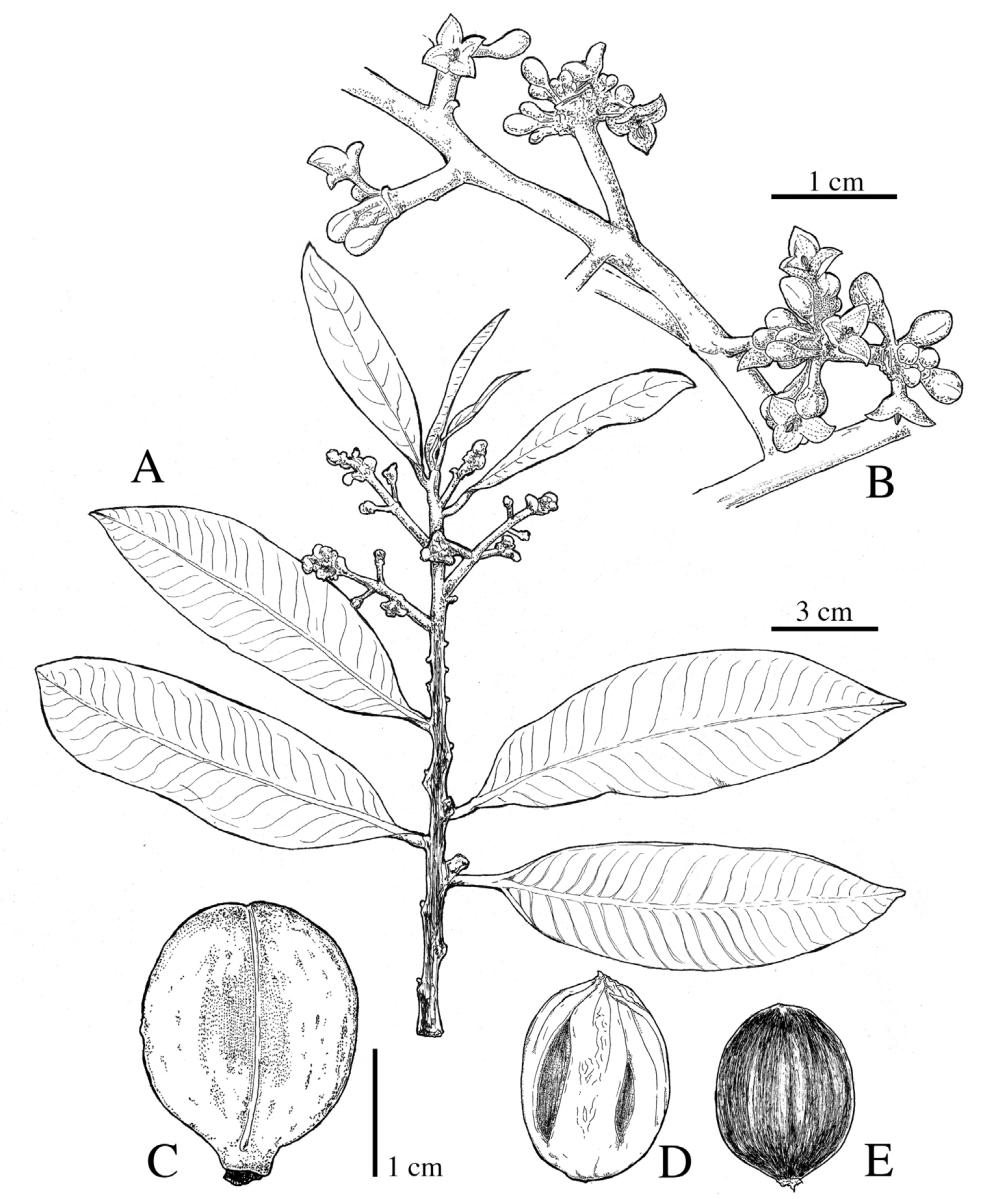
*Virola fosteri***A** branch with leaves and inflorescences **B** partial inflorescences **C** fruit **D** seed with aril **E** seed. Drawn by Pedro Juarez, based on *C. Galdames et al. 6422* (**A–E**).

#### Distinctive characters.

**Figure 16.**
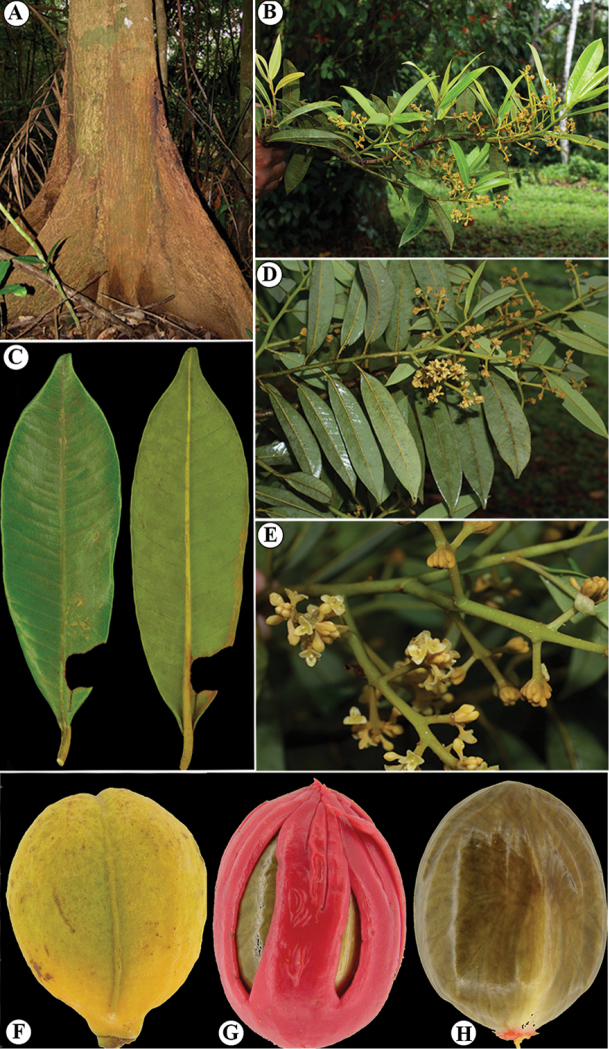
*Virola fosteri***A** lower trunk and buttresses **B** branch with staminate inflorescences **C** leaf blades showing adaxial (left) and abaxial (right) surface; also demonstrating the revolute base **D** branch with staminate inflorescences and leaf blades on abaxial surface **E** close up of staminate inflorescences **F** fruit **G** aril covering the seed **H** seed Photos by Rolando Pérez (**A**), Carmen Galdames (**B, D, E**), Steven Paton (**C, F, G, H**); all photos from https://stricollections.org/portal/index.php.

*Virola fosteri* is recognised by its small leaf blades (7.8–12 × 1.4–2.7 cm) and fruits (1.5–2.3 × 1.2–1.8 cm) (Figs 4I and 16F–H), as well the stellate, sessile trichomes on the abaxial surface of the leaf (Fig. 3E). It is also distinguished by its leaf blades that have numerous lateral veins (16–24 per side) that are prominent on adaxial surface, the revolute leaf margin and the base (Fig. 16C), the midvein that is laterally compressed adaxially and sometimes resembling a cutting edge; staminate flowers with a filament column (0.9–1.3 mm long) that is longer than the anthers (0.6–0.9 mm long); and its thick pericarp (1.5–2.5 mm).

#### Etymology.

The specific epithet honours one of its collectors, Robin B. Foster (1945–), ecologist and botanist at Field Museum in Chicago (F) who pioneered the cataloguing of the flora of Barro Colorado Island (BCI) in Panama, where *V. fosteri* occurs. Robin noted on one of his collections (*R. B. Foster 2931*) that it could represent a new species. In addition, on the same herbarium sheet, he observed one of the taxonomic characters that we here use to distinguish this as a new species: “*Leaves are consistently small throughout the tree and on juvenile plants*.”

#### Distribution.

*Virola fosteri* is known from Costa Rica (Limón) and Panama (Bocas del Toro, Colón, Panamá, San Blas and Veraguas) (Fig. 9E). It is found on the Caribbean slope from 0–350 (–800) m elevation.

#### Preliminary conservation status.

*Virola fosteri* is Vulnerable following IUCN criterion B2a. While the EOO for this species is large (25,645 km^2^), the small AOO (40 km^2^) with only eight known localities warrants its conservative status.

#### Common names.

Panama: bogamani, fruta dorada.

#### Phenology.

Flowering of *Virola fosteri* has been recorded in January to April, June and October and production of fruits in January to April.

#### Field characters.

Plants are large trees with tall buttresses. Bark exudes reddish watery exudate when damaged. Their small leaves are white or grey below. Flowers have pale orange or yellow perianth. The mature fruit is yellowish or golden brown with a red aril and brown seed.

#### Discussion.

In addition to the characteristics presented in diagnosis, *Virola fosteri* tends to have a higher number of and sunken (vs. plane) lateral veins per side than *V. multiflora* (16–24 vs. 10–18 per side), denser trichomes with longer branches on the abaxial leaf surface (Fig. 3E, K) and larger fruits [1.5–2.3 × 1.2–1.8 cm vs. 1.3–1.9 × 0.9–1.2 (–1.4) cm] (Fig. 4I, J).

It is also comparable to *V. micrantha* A. C. Sm. from Colombia due to the similar size of the leaf blades, which also have sessile stellate trichomes on the abaxial surface and short staminate inflorescences. *Virola micrantha* is a name apparently ignored in recent publications (ter Steege et al. 2016, 2019; Ulloa Ulloa et al. 2017; Gradstein 2016). Until recently, *V. micrantha* was only known from the type specimen (*R. E. Schultes & G. A. Black 46-377*, US*); however, via a herbarium study at MO, we identified additional Colombian material with staminate flowers (*R. Jaramillo et al. 7846*, MO-2 sheets!) and can also extend its distribution to Venezuela (*E. Marín 571* [MO!], *R. L. Liesner 6778* [MO!] and *J. Velazco 851* [MO!]). *Virola fosteri* differs from this species by its attenuate leaf base and acute to acuminate apex (vs. obtuse on both sides, also mucronulate at the apex), longer perianth of staminate flowers (2–2.5 [–3] mm vs. ca. 1 mm long) and longer anthers (0.6–0.9 mm vs. ca. 0.3 mm long) (Smith 1953).

*Virola coelhoi* W. A. Rodrigues (Colombia; *S. Defler 411*, MO!. Peru; *C. Grández & N. Jaramillo 2787*, MO!) from Brazil, and *V. parvifolia* Ducke (Brazil) are other species with similarly-sized leaves with revolute margins, traits shared with *V. fosteri*. Additionally, with *V. coelhoi*, which it is more likely to be confused, *V. fosteri* shares overall leaf shape and the type of trichomes on the abaxial surface (i.e. stellate, sessile and yellowish). The new species is distinguished from *V. coelhoi* by the abaxial surface that is pale brown to reddish-brown when dry and puberulent (vs. abaxial surface yellowish, very densely pubescent), staminate inflorescences with small bracts (2–5 × ca. 2.5 mm vs. 2.5–9 × 4–6 mm), and staminate flowers with longer filament columns [0.9–1.3 mm vs. (0.3–) 0.6–0.7 long] and anthers (0.6–0.9 mm vs. 0.4–0.5 mm long). It can be differentiated from *V. parvifolia* by its leaf blades and inflorescences that are glabrous or nearly so (vs. pubescent in *V. fosteri*) (Ducke 1936); additionally, Ducke (1936) mentions that *V. parvifolia* has numerous small granules or tubercles on the branches, leaf blades and peduncles that are lacking in *V. fosteri*.

Jiménez (2007; as Virola sp. B) mentions that the new species is similar to *Virola pavonis* (A. DC.) A. C. Sm. from South America. This is probably because both species have sessile and stellate trichomes on the abaxial surface of the leaf, sometimes a similar leaf size, a similar number of lateral veins (though there is a tendency towards higher numbers of veins in *V. pavonis*) and the length of the filament column and anthers. However, the new species differs in its shorter staminate inflorescences [2.5–5.3 cm vs. (3–) 7–15 cm long], smaller fruits (1.5–2.3 × 1.2–1.8 cm vs. 2.5–5 × 1.5–2.5 cm) and in the canaliculate or smooth line of dehiscence (vs. carinate).

In Mesoamerica, other species with leaf blades that are covered with stellate and sessile trichomes on the abaxial surface (Fig. 3E, F, L) and a filament column that is longer than the anthers are *V. guatemalensis* and *V. nobilis*. However, these two species have larger leaf blades (12.3–17.2 [–27.5] cm vs. 7.8–12 cm long) and fruits ([2.1–] 2.3–2.7 [–3.1] cm vs. 1.5–2.3 cm long). Similarities with *V. nobilis*, specifically, include their distribution pattern (at least in Panama), leaf blades with more lateral veins per side (20–30 [25–32] vs. 16–24) that are markedly elevated abaxially (vs. flat to slightly elevated), the leaf margin and base that are usually not revolute (vs. revolute) and fruits that generally have thick pericarp (2.5–3.5 mm vs. 1.5–2.5 mm).

#### Notes.

The species referred to as *Virola* sp. B in the *Manual de Plantas de Costa Rica* (Jiménez 2007) and as *V. multiflora* (*G. de Nevers 7608*, MO!) in the *Catálogo de las plantas vasculares de Panamá* (Correa et al. 2004) correspond to *V. fosteri*.

#### Specimens examined.

**Costa Rica. Limón**: Talamanca. San Miguel, Asacode, sendero a San Miguel, 30–100 m elev., 18 Jan 1997 (♂ fl), *J. González et al. 1632* (CR!, MEXU!*, MO!); Lomas Mreduk (La Pera), antiguo campo de exploración petrolera, 300–350 m elev., 06 Oct 2002 (fl), *J. Gómez-Laurito et al. 13903* (USJ!); cerros al sur del camino entre Puerto Viejo y Manzanillo por un camino nuevo hacia Bribri, 100 m elev., 18 Jan 1992 (fr), *B. Hammel 18392* (CR-2 sheets!, MEXU!*, MO!). **Panama. Bocas del Toro**: Parcela ubicada a 10 km de la desembocadura de la quebrada Boca Chica en la margen izquierda del río Changuinola, 550 m elev., 23 Oct 2007 (fl), *R. Aizprúa et al. 3398-RA* (PMA!*, US!*); ibid, 23 Oct 2007 (fl), *N. Daguerre et al. 660-ND* (PMA!*); along road to Chiriquí Grande, ca. 1.5 miles along side road east of highway, 250–300 m elev., 24 Jun 1986 (♂ fl), *G. McPherson & B Allen 9646* (MO!). **Colón**: San Lorenzo, no elev., 15 Jun 2009 (fr), *J. Lezcano & E. Spear 593* (PMA!*); Donoso, Teck Cominco Petaquilla mining concession, 300 m elev., 22 Feb 2008 (♂ fl), *G. McPherson & M. Merello 20148* (MO!, PMA!*); Donoso, westernmost part of province, site of proposed copper mine (INMET), 150 m elev., 12 Apr 2009 (imm fr), *G. McPherson 20913* (MO!, PMA!*); camino viejo de Piñas-Sherman, no elev., 22 Sep 2013 (fl), *R. Pérez et al. 1130* (MO n.v., PMA!*, SCZ!*). **Panamá**: Zona del Canal, Barro Colorado Island, slope between AVA 7 and FD 5, [10–100 m elev.], 30 Mar 1979 (fr), *R. B. Foster 2931* (MO!, PMA!*); Barro Colorado Island, Wetmore trail, [10–100 m elev.], n.d. 1980 (♂ fl), *R. B. Foster 2946* (CR!, F!*, MO!, PMA!*, U!*, US!*); Barro Colorado Island, Drayton, Drayton 18–19, [10–100 m elev.], 31 Mar 1988 (fr), *N. Garwood 2301A* (PMA!*). **San Blas**: El Llano Cartí road, km 26.5, no elev. 10 Apr 1985 (fr), *G. de Nevers et al. 5285* (INPA!*, MEXU!*, MO!, PMA!*); El Llano-Cartí road, km 32.3, 200 m elev., 02 Mar 1986 (♀ fl, imm fr), *G. de Nevers 7226* (INPA!*, MO!, PMA!*); Cangandi, 30 m elev., 27 Mar 1986 (♀ fl, imm fr), *G. de Nevers et al. 7608* (MO!). **Veraguas**: Santa Fe, near the entrance to the agriculture school, Alto de Piedra, [800 m elev.], 26 Feb 1975 (fr), *S. Mori & J. Kallunki 4891* (MO!).

### 6. Virola guatemalensis

Taxon classificationPlantaeMagnolialesMyristicaceae

(Hemsl.) Warb.

55D4AA71-9DD1-5A3E-BD31-1FDFAEFE9F58

Fig. 10E–G

Virola guatemalensis (Hemsl.) Warb. Nova Acta Acad. Caes. Leop.-Carol. German. Nat. Cur. 68: 220. 1897.
Myristica guatemalensis Hemsl. Biol. Cent.-Amer., Bot. 3. 66–67. 1882. Type. Guatemala. [no specific data in location], [no date], [seeds], [*G. U.*] *Skinner s.n.* (holotype: K!*).

#### Distinctive characters.

*Virola guatemalensis* is distinguished by many characters of its leaf, including overall size [12.3–17.5 (–24.1) × (2.4–) 3.8–5.5 (–8.9) cm], inconspicuous pubescence of tiny stellate and sessile trichomes on abaxial surfaces (Fig. 3F), 13–21 lateral veins on each side of the leaf that are barely distinctive abaxially (Figs 8F, 10F) and slightly revolute margins. Additionally, the staminate flowers have a filament column that is longer (1–1.2 mm long) than the anthers (0.5–0.8 mm long) and fruits that are 2.7–3.4 × 1.7–2.3 cm, ellipsoid (Fig. 4G), commonly glabrous or glabrous distally and with pubescence at the base with the line of dehiscence slightly carinate or smooth and a thin pericarp [0.4–1 (–2.5) mm thick].

#### Distribution.

*Virola guatemalensis* is known from Mexico (Chiapas, Oaxaca and Veracruz), Guatemala (Sololá) and Honduras (Yoro) (Fig. 9F). It has been recorded between 150–1250 m elevation. Standley and Steyermark (1946) mention that it also occurs in Alto Verapaz, Suchitepéquez, San Marcos and Huehuetenango in Guatemala, while Standley (1931) indicates that it occurs in Lancentilla (Honduras), where it is noted to be one of the most common tree species. While Gentry (2001) postulated that *V. guatemalensis* is an expected species for Nicaragua, no Nicaraguan collections of this species are known.

#### Common names.

Mexico: cacao, cacao volador (Chiapas), cacaotillo, cedrillo (Veracruz), k’ik’ che’ [Lacandon name]. Guatemala: chucul, palo de sebo, cacao volador, cacao cimarrón. Honduras: sangre.

#### Phenology.

Flowering of *Virola guatemalensis* has been recorded in April and May and fruit production in January, March, August, October and December.

#### Field characters.

Plants are trees between 12–35 m high and 45–130 cm DBH with a straight trunk, sometimes with moderately sized buttresses. The bark is variously described as smooth or fissured and scaly and is brown to greyish-brown in colour and exudes watery reddish transparent sap when damaged. Flowers have yellow, green-yellowish or brown perianth. The mature fruit is yellow with a red aril.

#### Discussion.

While the name *Virola guatemalensis* has been applied to herbarium specimens from Costa Rica and Panama in the past, those are here interpreted as a distinct species, *V. montana*. Based on our interpretation, *V. guatemalensis* is restricted to Mexico, Guatemala and Honduras. It is distinguished from *V. montana* by a series of characters, described below. While *V. laevigata* was typified with material from the Pacific slope in Chiriquí, Panama and is frequently considered a synonym of *V. guatemalensis* (e.g. Smith and Wodehouse 1938; Standley and Steyermark 1946; Duke 1962), we treat these as morphologically distinct; differences between these two species are discussed under *V. laevigata*.

#### Notes.

The specimens, identified in Gómez-Laurito and Ortiz (2004), Haber (2014) and Monro et al. (2017) as *V. guatemalensis* or *V. surinamensis*, correspond to *V. montana* (*R. Aguilar 1131*, [*E.*] *Bello 470, M. Chinchilla 3, J. Gómez-Laurito 11846, 11970*, *J. González 823*) or *V. amistadensis* (*A. Chacón 194*). Additionally, a specimen cited as *V. guatemalensis* in *Flora of Panama* (Duke 1962) corresponds with the type of *V. laevigata* (*G. P. Cooper & G. M. Slater 308*).

The specimen *J. A. Steyermark 47624* (MO-3 sheets!) seems to represent two different individuals – two of the sheets have staminate flowers, while the third has immature fruits. The duplicate of this collection at the Field Museum (digital image) has two sheets, both from a pistillate individual: one sheet carries flowers and the other immature fruits.

*Virola guatemalensis*, along with another Mesoamerican species, *V. koschnyi* (see below), produce the largest grains of pollen in *Virola* (Walker and Walker 1979).

#### Selected specimens seen.

**Mexico. Chiapas**: Tila, Chewupaj, 1000 m elev., 10 Dec 1982 (fr), *A. Méndez 5223* (MEXU!*, MO!); Peltalcingo, slope of Ahk’ulbal Nab above Peltalcingo, 1700 m elev., 27 Feb 1981 (fr), *D. Breedlove 49868* (MEXU!*); La Trinitaria, 10 km east northeast of Dos Lagos above Santa Elena, 1170 m elev., 19 Jan 1982 (fr), *D. Breedlove & F. Almeda 57553* (MO!); La Independencia, ridge, 45–50 km E of Lagos de Montebello National Park on road to Ixcán from Santa Elena, 760 m elev., 22 Jan 1982 (fr), *D. Breedlove & F. Almeda 57735* (MO!). **Oaxaca**: Matías Romero Avendaño, ± 11 Km. al S de Aserradero La Floresta, ± 24 Km. al S de Esmeralda, lomas al S del Río Verde, 300 m elev., 24 Apr 1981 (fl), *T. Wendt et al. 3235* (MEXU!*). **Veracruz**: Cima del cerro Vigia, 450 m elev., 19 Apr 2005 (♂ fl), *E. Velasco-Sinaca 678* (MEXU!*, MO!); La Escondida, 3 km NO de Estación de Biología Tropical Los Tuxtlas, 200 m elev., 05 Apr 1983 (♂ fl), *G. Ibarra 604* (MEXU!*, MO!); Estación de Biología Tropical Los Tuxtlas, 200 m elev., 29 Oct 1983 (fr), *G. Ibarra 957* (MO!); camino Laguna Escondida, 2 km NW de la Estación de Biológia Tropical Los Tuxtlas, 200 m elev., 08 May 1984 (♂ fl), *G. Ibarra & G. Gómez 1607* (MO!); Ocotal Grande, 5 km N de Mecayapan, 1000 m elev., 14 Mar 1985 (fr), *G. Ibarra et al. 2357* (MEXU!*, MO!, NO!); 2 km al NW del rancho Rubén Sánchez, 250–350 m elev., 28 May 1985 (♂ fl), *G. Ibarra 2450* (MO!); Santiago Tuxtla, Alta Luz, 540 m elev., 11 May 1968 (♂ fl), *M. Sousa 3680* (MO!); Catemaco, Tebanca, no elev., 20 Oct 1971 (fr), *J. I. Calzada 615* (MO!); Cerro El Vigia de Santiago Tuxtla, 800 m elev., 18 Feb 1967 (fr), *R. Cedillo 9* (MO!); Catemaco, El Chinchero, 14.2 km al SE de Tebanca camino al río Huacinapan, no elev., 19 Dec 1984 (fr), *R. Cedillo & G. Pérez 2958* (MEXU!*, MO!, NO!); Hidalgotitlán, lomitas al SE de Poblado 6, 150 m elev., 27 Apr 1982 (♂ fl), *T. Wendt et al. 3898* (LSU!, MO!). **Guatemala. Sololá**: Bordering barranco on Finca Olas de Mocá, just west of Finca Mocá, south-facing slopes of Volcán Atitlán, 1000–1100 m elev., 15 Jun 1942 (♂ fl, imm fr), *J. A. Steyermark 47624* (F!*, MO-3 sheets!). **Honduras. Yoro**: Cascada de Río Guán Guán, 400–440 m elev., 20 Apr 1995 (♂ fl), *T. Hawkins & M. Merello 768B* (MO!).

### 7. Virola koschnyi

Taxon classificationPlantaeMagnolialesMyristicaceae

Warb.

A5F614C7-BCBE-54FE-9642-232C771993C1

Fig. 17

Virola koschnyi Warb. Repert. Spec. Nov. Regni Veg. 1: 71. 1905. Type. Costarica [Costa Rica]. [Alajuela] San Carlos, *Th. Koschny s.n.* (lectotype, here designated: F!*, fragment with B photo [649050, F0360191F]); Costa Rica. Heredia. [Sarapiqui] Parque Nacional Braulio Carrillo, bosque primario frente al Puesto La Ceiba, 450 m elev., 23 Dec 1988 (♂ fl), *M. Ballestero 72* (epitype, designated here: CR! [16354]; isoepitypes: CR! [156734], MO! [5550256, MO-299037].
Virola merendonis Pittier., Contr. U.S. Natl. Herb. 20: 453. 1922. Type. [Honduras]. [Copán:] Collected in the forests of Cuchillitas, between Arranca Barba Hills and Mohanes, in the Cordillera de Merendon, borders of Guatemala and Honduras, in fruit, [10–] 18 May 1919 [fr], *H. Pittier 8530* (holotype: US!*; isotype: NY!*).
Virola costaricensis nomen nudum

#### Distinctive characters.

*Virola koschnyi* can be recognised by the densely tomentose leaf undersides with pediculate, dendritic trichomes (Fig. 3G) that are soft to the touch, the numerous lateral veins [(16–) 20–35] that form a clear submarginal vein and tertiary veins that are usually inconspicuous abaxially (Figs 3G, 8G). Adaxially, the new leaves are densely covered with dendritic sessile or subsessile trichomes that are deciduous at maturity, resulting in glabrous to glabrescent and smooth adxial surface on mature leaves. The staminate flowers have filament columns that are 0.7–0.9 (–1.4) mm long with anthers 0.5–0.7 (–1) mm long. Its fruits are 1.9–3.1 × 1.5–1.9 cm and ellipsoid or subglobose (Fig. 4F), with a pericarp 1.2–3.1 mm thick.

**Figure 17.**
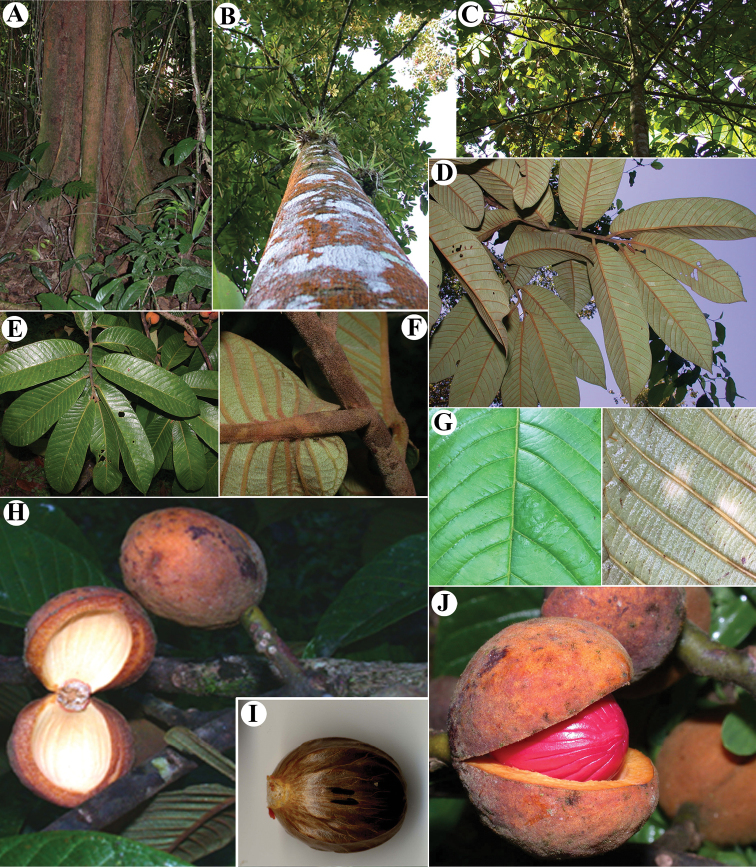
*Virola koschnyi***A** lower trunk and buttresses **B** trunk **C** branching **D** leaf blades on abaxial surface **E** leaf blades on adaxial surface **F** twig, petiole and leaf base **G** leaf venation on adaxial surface (left) and abaxial surface (right) **H** branch with fruits, showing the interior part of the fruit **I** seed **J** mature fruit. All photos by Reinaldo Aguilar from https://sura.ots.ac.cr/florula4/index.php, except **G** by D. Santamaría-Aguilar.

#### Distribution.

**Figure 18.**
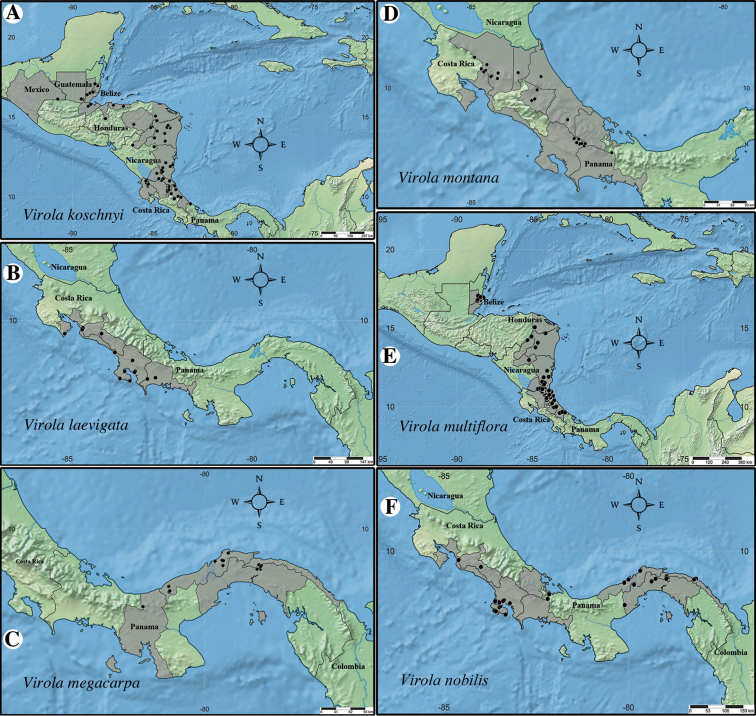
Geographic distribution of *Virola koschnyi* (**A**), *V. laevigata* (**B**), *V. megacarpa* (**C**), *V. montana* (**D**), *V. multiflora* (**E**) and *V. nobilis* (**F**).

*Virola koschnyi* is known from Mexico (Chiapas), Guatemala (Izabal and Petén), Belize (Cayo, Toledo and Stann Creek), Honduras (Atlántida, Cortés, Gracias a Dios and Olancho), Nicaragua (Atlántico Norte, Atlántico Sur, Jinotega and Río San Juan), Costa Rica (Alajuela, Guanacaste, Heredia, San José and Limón), and Panamá (Bocas del Toro) (Fig. 18A). It is found mainly on the Caribbean slope, where it has been recorded between 10–1000 (–1700) m elevation.

We consider this species restricted to Mesoamerica. The collections identified as *V. koschnyi* from Ecuador in *Catalogue of the Vascular Plants of Ecuador* (i.e. *C. H. Dodson et al. 6465* [RPSC, SEL]; Jørgensen and León-Yánez 1999) correspond to *V. multinervia* Ducke (Jaramillo et al. 2004). Determinations of *V. koschyni* cited for Colombia by Cogollo et al. (2007) are doubtful and do not cite voucher specimens, while those of Gradstein (2016) could not be confirmed.

#### Common names.

Guatemala: Cedrillo, drago, sangre. Belize: banak, black banak. Honduras: bának, sebo, sangre de montaña, sangre real. Nicaragua: banak blanco, banak colorado, cebo, fruta dorada, sebo. Costa Rica: achiotillo, fruta dorada.

#### Phenology.

Flowering of *Virola koschnyi* has been recorded in January, March and May, July, September, October and December. Only three herbarium specimens with pistillate flowers were seen, two from Costa Rica and one from Nicaragua. Fruits are produced throughout the year, though most often collected in March, May and June.

#### Field characters.

Plants are trees (4–) 10–70 m tall and 12–80 cm DBH, with a trunk that is straight with well-developed buttresses. The bark is described as scaly and falling off in small plates or as smooth, red-brown or whitish in colour; clear, red or reddish exudate is released when damaged. Flowers usually have yellow perianth, though it can sometimes be white, brown, or orange. The mature fruit is yellow, orange or brown, with a red or pink-red aril. The testa of the seed is black, brown or white.

#### Typification.

*Virola koschnyi* was described by Otto Warburg based on a collection made by Theodor Koschny (Warburg 1905). Warburg indicated in the protologue “Costarica: San Carlos, leg. Th. Koschny,” but did not mention where the type was housed. The type is presumed to have been housed at Berlin (B) and subsequently destroyed during World War II.

The only known original material of *Virola koschnyi* is a fragmentary specimen accompanied with a photograph at the Field Museum. The photo shows the original specimen was composed of three fertile branches with leaves (two with inflorescences and one with fruit), with a handwritten annotation of “*V. costaricensis* Warb”, presumably by Warburg, though this name was never validly published. The fragment material consists of pieces of leaves, two inflorescence branches with a few immature flowers and a broken fruit comprising pericarp, testa and seed. Since they are mounted with the photo of the Berlin specimen, these fragments presumably originated from the holotype. Although it is not ideal type material due to its fragmentary nature, the specimen at F appears to be the only extant original material and is sufficient to confirm that it coincides with the protologue and concept of the species used and so is here designated as lectotype. In order to ensure the precise application of the name, given the fragmentary type material, an epitype was selected from the studied specimens.

#### Notes.

Most of the specimens from the Pacific slope of Costa Rica and Panama previously identified as *V. koschnyi* are here interpreted as *V. chrysocarpa*. The similarities and differences between *V. koschnyi* and V. *chrysocarpa* are discussed under the latter species. While most fruiting specimens of *V. koschnyi* have obtuse to rounded apices, *G. Herrera 1092* (CR!, MO!) from Costa Rica and *R. Rueda & H. Mendoza 17069* (MO!) from Nicaragua have apiculate apices. Aside from the fruit apex, these specimens conform to the species concept adopted here in all other characters (e.g. number of lateral veins, submarginal vein and tertiary veins that are usually inconspicuous abaxially and pediculate, dendritic trichomes).

#### Selected specimens seen.

**Mexico. Chiapas**: Ocosingo, En ejido Chaju, 150 m elev., 17 Mar 1993 (fl), *E. Martínez & C. H. Ramos 26336* (MEXU-4 sheets!*); Boca de Chajul, 800 m al SE del poblado, 350 m elev., 21 May 1992 (fr), *G. Domínguez 438* (MEXU!*). **Belize. Cayo**: At mile 28.5 m on Hummingbird highway, 200–300 ft [60–90 m] elev., 14–21 Jun 1973 (fr), *T. B. Croat 24562* (MO!). **Stann Creek**: Mountain Cow, [200 m elev.], 25 Mar 1940 (imm fr), *P. H. Gentle 3277* (MO!); Big Eddy Ridge, [50–200 m elev.], 21 May 1940 (fr), *P. H. Gentle 3347* (MO!); 22 miles Stann Creek, 200 ft [60 m] elev., 27 Jun 1927 (fr), *W. A. Schipp 949* (MO-2 sheets!). **Toledo**: Bladen Nature Reserve, 20 m from the Bladen River, 45 m elev., 27 Feb 1997 (♂ fl), *S. W. Brewer 178* (MO!); Southern Maya mountains, Bladen Nature Reserve, 250 m elev., 23 May 1996 (fr), *G. Davidse 36211* (MO!). **Guatemala. Izabal**: Between Seja and Fronteras, no elev. 08 May 1971 (fl bud), *E. Contreras 10751* (MO!); Livingston, Creek Jute, Biotopo Chocón Machacas, [0–5 m elev.], 28 Jul 1988 (fr), *P. Tenorio et al. 14953* (MEXU!*, MO!). **Petén**: Chinchila, Sebol road, [20 m elev.], 16 May 1967 (fr), *E. Contreras 6924* (MO!); La Cumbre, [300 m elev.], 30 Jul 1969 (fr), *E. Contreras 8790* (MEXU!*, MO!). **Honduras. Atlántida**: Lancetilla S of Tela, 20–50 m elev., 25 Jun 1970 (fr), *G. Davidse & R. Pohl 2178* (MO!). **Cortés**: [Santa Cruz de Yojoa], 660 m elev., 13 Feb 1952 (fl), *P. H. Allen 6456* (GH!*, EAP-2 sheets!*). **Gracias a Dios**: La Mosquitia, Ahuas Bila, 100 m elev., 5–13 May 1985 (fr), *C. Nelson & G. Cruz 9484* (MO!). **Olancho**: San Esteban, montaña El Carbón, 460 m elev., 02 Feb 1994 (fl bud), *A. Meras 02* (MO); a 3 km comunidad El Carbón, 430 m elev., 12 Mar 1997 (fl), *I. Rivas 08-C1* (EAP!*). **Nicaragua. Atlántico Norte**: Bonanza, Musawas, 50–150 m elev., 17 Oct 2002 (♂ fl), *C. Aker et al. 624* (MO!); Siuna, Waspado, 100–120 m elev., 06 Oct 1982 (imm fr), *F. Ortíz 252* (MO!); sur de río Wawa, 40 m elev., 16 Mar 1971 (imm fr), *E. L. Little Jr. 25164* (MO!). **Atlántico Sur**: Kurinwac[s]ito, 80–100 m elev., 18–22 Mar 1984 (♀ fl, fr), *P. P. Moreno 23682* (MO), *P. P. Moreno 23701* (MO!); El Zapote, 40 km NE de Nueva Guinea, 130–150 m elev., 29 Feb 1984 (♂ fl), *J. C. Sandino 4808* (MO!). **Estelí**: El Zacatón, entre Mesas Plan Helado y la laguna de Miraflor, 1300–1400 m elev., 29 Mar 1983 (imm fr), *P. P. Moreno 23788* (MO!). **Jinotega**: Cua Bocay, Reserva de Bosawas, comunidad de San Andrés, 180 m elev., 30 Jun 2005 (fr), *I. Coronado et al. 1951* (MO!). **Río San Juan**: El Castillo, Reserva Indio Maíz, 100–200 m elev., 15 Mar 1999 (♂ fl), *R. Rueda el al. 10319* (MO!); San Juan del Norte, Reserva Indio Maíz, 30 m elev., 18 May 2002 (fr), *R. Rueda & H. Mendoza 17069* (MO!); río Sábalos, 2 km de Santa Eduviges, 80 m elev., 18 Feb 1984 (♂ fl), *P. P. Moreno 23042* (MO!). **Costa Rica. Alajuela**: Parque Nacional Rincón de La Vieja, 900–1000 m elev., 07 Mar 1988 (♀ fl), *G. Herrera 1607* (CR!, MO!); Upala, Dos Ríos, 500 m elev., 02 Nov 1987 (fr), *G. Herrera 1092* (CR!, MO!); Upala, Estación San Ramón, 550 m elev., 27 Jan 1995 (fr), *F. Quesada 243* (CR!, LSU!, MO!); Upala, Finca San Gerardo, 600 m elev., 20 Feb 1998 (fl bud), *F. Quesada et al. 577* (CR-2 sheets!, MO!, USJ!). **Cartago**: Turrialba, CATIE, 650 m elev., 08 Feb 1975 (fr), *C. Vaughan 93* (USJ!); Bosque de Florencia de Turrialba, 650 m elev., 31 Jul 1969 (fr), *R. Ramalho 7990* (USJ!). **Guanacaste**: Santa Cruz, road from Santa Cecilia to La Esperanza, 340 m elev., 29 Dec 1989 (fr), *R. E. Gereau et al. 3455* (CR-2!, MO!); Parque Nacional Guanacaste, Estación Pitilla, 700 m elev., 02 Mar 1991 (♂ fl), *P. Ríos 309* (CR-2 sheets!, MO!). **Heredia**: Parque Nacional Braulio Carrilo, Estación El Ceibo, 500–600 m elev., 01 Oct 1989 (fl bud), *R. Aguilar 9* (MO!, LSU!, USJ!); Los Arbolito, al N de Puerto Viejo, 20 m elev., 09 Mar 1993 (♂ fl), *F. Araya 197* (CR-2 sheets!, LSU!, MO!); between La Selva entrance and bridge before Puerto Viejo, [100 m elev.], 18 Apr 1981 (fr), *J. Folsom 9791* (MO!). **Limón**: Refugio de Vida Silvestre Gandoca Manzanillo, sendero Cerillo, 1 m elev., 03 Mar 1999 (fr), *U. Chavarría 1918* (CR-2 sheets!, MO!); Cordillera de Talamanca, ridge separating Río Madre de Dios from Quebrada Cañabral, 440 m elev., 02 Sep 1988 (♀ fl), *M. Grayum et al. 8692* (CR-2 sheets!, MO-2 sheets!, USJ!); Sixaola, Quebrada Mata de Limón, 20–40 m elev., 27 Jan 1987 (fr), *M. Grayum et al. 8004* (CR!, MO!); Río Peje, no elev., 14 Jun 1979 (fr), *R. Ocampo 2582* (CR!); Parque Nacional Tortuguero, Estación Agua Fría, 40 m elev., 02 Feb 1988 (fr), *R. Robles 1587* (CR!, MO!); Cerro Coronel, 20–170 m elev., 16–23 Jan 1986 (♂ fl), *W. D. Stevens 23768* (CR, MO). **San José**: Z. P. [Zona Protectora] La Cangreja, Santa Rosa de Puriscal, 350 m elev., 01 Oct 1992 (fr), *J. F. Morales 764* (CR). **Panama. Bocas del Toro**: Isla Colon, camino central, unos 4 km de Boca de Drago, 2–25 m elev., 16 Mar 1993 (fr), *R. Foster et al. 14549* (F!*).

### 8. Virola laevigata

Taxon classificationPlantaeMagnolialesMyristicaceae

Standl.

CA381C53-D596-526F-9741-79D43FC36DB2

Fig. 19

Virola laevigata Standl. Publ. Field Mus. Nat. Hist., Bot. Ser. 4(8): 209. 1929. Type. Panama. Province of Chiriquí, Progreso, [July–Aug.] 1927 [♂ fl], *G. P. Cooper & G. M. Slater 308* (holotype: F!*; isotypes: NY!*, WIS!*, US!*).

#### Distinctive characters.

*Virola laevigata* is distinguished by its glabrous or nearly glabrous vegetative parts (i.e. twigs, mature leaf blades on both surfaces [Fig. 3H], petioles); when trichomes are present, they are primarily on the leaf buds or very new leaves. Additionally, leaves have 12–20 lateral veins and margins and bases that are slightly revolute, staminate flowers have a straight filament column that is longer (0.8–1.3 mm) than the anthers (0.5–0.7 mm) and relatively small fruits (1.8–2.9 × 1.5–1.8 cm) (Fig. 4M) with pericarp that is 1.8–2.8 mm thick.

#### Distribution.

*Virola laevigata* is known from Costa Rica (Puntarenas and San José) and Panama (Chiriquí) (Fig. 18B). It is found on the Pacific slope, where it has been recorded between 0–500 (1600?) m elevation. Jiménez (2007) suggested that the maximum elevation for this species in Costa Rica is 1600 m, potentially based on *L. González 3089* from Cerro Turrubares (San José province) (B. Hammel pers. comm., Aug. 2019); however, we have not found any specimen that occurs this high.

#### Common names.

Costa Rica: fruta dorada. Panama: bogamani.

#### Phenology.

Flowering of *Virola laevigata* has been recorded in January, May, July and November. Fruits are produced from December to February. Pistillate flowers were not present on herbarium sheets studied.

**Figure 19.**
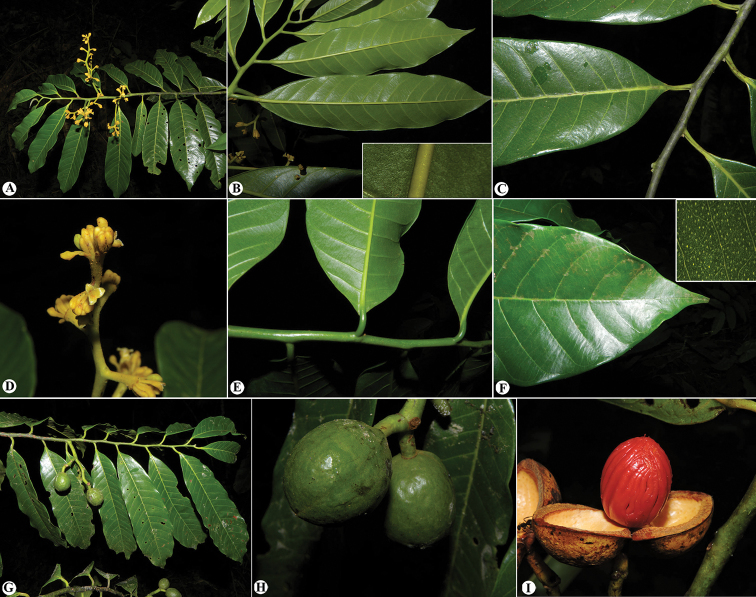
*Virola laevigata***A** branch with staminate inflorescences **B** leaf blades on abaxial surface, inset showing the glabrous midvein **C** twig, petiole and leaf on adaxial surface **D** close up of staminate inflorescences **E** leaf base **F** leaf apex, inset showing leaf punctations **G** branch with immature fruits **H** close-up of immature fruit **I** open fruit, showing the aril. Photos by Reinaldo Aguilar.

#### Field characters.

Plants are trees 9–40 m tall and 35–60 cm DBH with a straight trunk and small (ca. 20 cm tall), triangular buttresses. The bark is described as finally grooved, smooth, flaking in vertical strips or scaly and is grey, blackish or reddish in colour, with exudate that is reddish or colourless and oxidising to reddish-cream. The leaves are bright green on both sides and have numerous pellucid dots that are most visible against the light. Flowers have yellow, yellow-brown or yellowish perianth, sometimes with a slight aroma in staminate flowers (*N.* Z*amora & T. D. Pennington 1583*, but the specimen label states pistillate flower). The mature fruit is yellow with a red aril (when immature, it is white). In the Osa Peninsula, where this species is frequent, it prefers riparian habitats.

#### Discussion.

*Virola laevigata* has traditionally been considered a synonym of *V. guatemalensis* (e.g. Smith and Wodehouse 1938; Standley and Steyermark 1946; Duke 1962), likely due to limited material. However, with new herbarium specimens, both species can be clearly distinguished vegetatively and with fruit characters. Vegetative material of *V. laevigata* can be distinguished by petioles and mature leaf blades that are abaxially glabrous (vs. diminutively pubescent with tiny stellate, sessile trichomes in *V. guatemalensis*). Their fruits also differ in size, shape and thickness of the pericarp; in *V. laevigata*, they are smaller (1.8–2.9 × 1.5–1.8 cm), ovoid or subglobose and with thick pericarp (1.8–2.8 mm); while in *V. guatemalensis*, they are large (vs. 2.7–3.4 × 1.7–2.3 cm), ellipsoid and with a thin pericarp [0.4–1 (–2.5) mm].

In addition to *Virola guatemalensis*, herbarium specimens of *V. laevigata* have been determined as *V. surinamensis* (interpreted here as *V. nobilis*). However, *V. laevigata* is distinguished by its glabrous or almost glabrous abaxial leaf surface (Fig. 3H, O) and mature fruits (vs. pubescent) and its tendency towards thinner pericarp [1.8–2.8 mm vs. 2.5–3.5 (–4.2) mm thick]. In the Osa Peninsula, where these two species grow together, they can be easily distinguished in the field: *V. laevigata* prefers riparian habitats, does not usually develop tall buttresses and the external bark has a greenish tone, while *V. nobilis* grows far from bodies of water, has tall buttresses and the external bark is reddish to brown. For a description and comparison of the bark of these two species, see Moya Roque et al. (2014), as *Virola* sp. A and *V. surinamensis*.

#### Notes.

The seedlings of *V. laevigata* are described by Ley López and Chacón Madrigal (2017) (though as “Virola sp. A”). Additionally, the species presented as “Virola sp. A” in Jiménez (2007) and as *V. surinamensis* in Jiménez Madrigal and Grayum (2002; RZ [=*R. Zuñiga*] 459) corresponds with *V. laevigata*. The illustration in Quesada Quesada et al. (1997) as *V. guatemalensis* is potentially also *V. laevigata*.

#### Selected specimens seen.

**Costa Rica. Puntarenas**: Golfito, 1 km antes de llegar a La Palma, 8 m elev., 16 Jan 1993 (fr), *R. Aguilar 1585* (CR-2 sheets!, LSU!, MO!); fila Carbonera, cabo Matapalo, 300 m elev., 16 Jul 1993 (♂ fl), *R. Aguilar 2004* (CR!, MO!); estación Sirena, 10 m elev., 12 Oct 1993 (fl bud, fr), *R. Aguilar 2490* (CR-2 sheets!, LSU!, MO!); Golfito, orillas del camino a las torres de comunicación, 450–500 m elev., 12 Jan 1999 (fr), *J. Gómez-Laurito & V. Mora 13191* (CR!); Playa Cacao, Fila entre quebrada Nazanero y el mar en Punta Voladora, 200 m elev., 26 May 1994 (♂ fl), *G. Herrera & G. Rivera 7076* (CR!); Parque Nacional Corcovado, Sirena, 10 m elev., 28 Feb 1989 (fr), *C. Kernan & P. Phillips 962* (CR-2 sheets!, MO!); Parque Nacional Corcovado, Sirena, Ollas Trail, 1–20 m elev., 19 Oct 1989 (fr), *C. Kernan & G. Fonseca 1288* (CR-2 sheets!, INPA!*, MO!); Jiménez, Piro, camino a Laguna Silvestre, [0–100 m elev.], 01 Feb 2012 (fr), *J. M. Ley-López 74* (USJ); Osa, fila Esquinas, 200 m elev., 26 May 1993 (♂ fl), *M. Segura & F. Quesada 69* (CR!, LSU!, MO!); Rincón de Osa, in vicinity of airstrip, 40 m elev., 25 Jul 1974 (fl bud), *J. Utley & K. Utley 1236* (CR!); Garabito, por Carara, cerca de la toma de agua, 97 m elev., 22 Nov 2006 (fl bud), *L. D. Vargas & D. Castillo 1870* (CR!); Osa, Uvita, cerca de Dominical, 200 m elev., 28 Jan 1991 (♂ fl), *N. Zamora & T. D. Pennington 1583* (CR-2 sheets!, INPA!*, MEXU!*, MO!); Puntarenas, Montezuma, camino a Cóbano por el río Montezuma, ca. 1.5 km oeste de la intersección con camino a Cabuya, 101 m elev., 11 Dec 2005 (fr), *B. Hammel & I. Pérez 23944* (CR!); Montezuma, por el Canopy, 100 m elev., 08 Jul 2006 (fl bud), *B. Hammel & I. Pérez 24149* (CR!). **San José**: Carara, sector Agrominas, sitio Carretera Costanera, 100 m elev., 20 Sep 1991 (fr), *R. Zuñiga 459* (CR-sheets!, LSU!). **Panama. Chiriquí**: Río Platanal-Bugaba, 10 Dec 1975 (fr), *M. M. Gutiérrez 21* (MO!).

### 9. Virola megacarpa

Taxon classificationPlantaeMagnolialesMyristicaceae

A. H. Gentry.

5FE769BF-14B0-5418-B4D5-ED9739AE500C

Fig. 20A, B

Virola megacarpa A. H. Gentry. Ann. Missouri Bot. Gard. 62(2): 474. 1975. Type. Panama. Colón: Santa Rita Ridge, 23 Mar 1972 [fr], [*A. H*.] *Gentry &* [J. D.] *Dwyer 4804* (holotype: MO!; isotypes: BM!*, MEXU!*, WIS!*).

#### Distinctive characters.

*Virola megacarpa* can be recognised by its large and oblong leaf blades (20.3–37 × 7–13 cm) with numerous [(32–) 40–50 per side], dense lateral veins and a densely pubescent abaxial surface with dark brown to ferruginous dendritic trichomes (Fig. 3I). It is also the species with the largest fruits (4–5.7 × 2–2.9 cm) in the region and likely the genus; these are also densely pubescent with an acuminate to rostrate apex (Fig. 4P) and thick pericarp (3–6 mm).

#### Distribution.

*Virola megacarpa* is only known from Panama (Colón, Panamá, San Blas and Veraguas) (Fig. 18C) from 50–550 m elevation.

This species is attributed to Colombia in Cogollo (2011), based on the specimen *J. Brand 1252* (JAUM!*; fr). It is also mentioned as occurring in Colombia in Gradstein (2016) and Ulloa Ulloa et al. (2017), though these references do not mention a voucher specimen (likely they refer back to the same specimen cited by Cogollo [2011]). The first author has seen a digital image of this specimen (*J. Brand 1252*, JAUM) and it appears that the leaf undersurface and fruits are scarcely pubescent, as well as smaller. This specimen clearly corresponds to a species of the group Surinamenses sensu Smith and Wodehouse (1938) and not *V. megacarpa*. For that reason, *V. megacarpa* is considered restricted to Mesoamerica.

**Figure 20.**
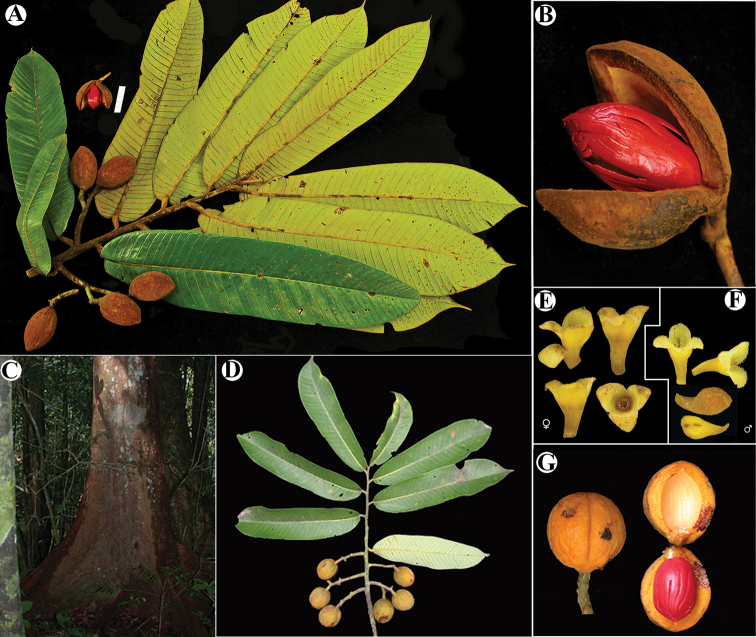
A. *Virola megacarpa***A** branch with fruits, both leaf blade surfaces shown **B** fruit. *Virola nobilis* from Barro Colorado **C** lower trunk and buttress **D** branch with fruits, leaf blades on both surfaces **E** pistillate flowers **F** staminate flower and bud **G** fruits. Photos by Carmen Galdames (**A, B**), Rolando Pérez (**C, D, G**), Steven Paton (**E, F**); all photos from https://stricollections.org/portal/index.php.

#### Common names.

None recorded.

#### Phenology.

The only observed herbarium specimen with flowers (these staminate) was collected in August. Fruits were collected in February and March and August to November.

#### Field characters.

Plants are trees 12–30 m tall and 21.5–53 cm DBH. Damaged bark releases exudate that is red or that oxidises reddish-brown. Flowers have pale yellow perianth. Fruits are densely pubescent with brown trichomes and a red aril.

#### Discussion.

Vegetatively, *Virola megacarpa* can be confused with *V. koschnyi* and some specimens have been identified as the latter (e.g. *G. de Nevers & H. Herrera 7917*, MO). Both species share leaf blades with numerous and conspicuous lateral veins and pediculate trichomes on the abaxial surface. However, *V. megacarpa* has more lateral veins per side [(32–) 40–50 vs. (16–) 20–35] and these are more closely spaced (Figs 8G and I) and fruits are larger (4–5.7 × 2–2.9 cm vs. 1.9–3.1 × 1.5–1.9 cm) (Fig. 4F, P).

#### Specimens examined.

**Panama. Colón**: East of Portobelo, 50–100 m elev., 12 Oct 1992 (fr), *G. McPherson & M. Richardson 15873* (MO!); Teck Cominco Petaquilla mining concession, 220 m elev., 20 Feb 2008 (fr), *G. McPherson & M. Merello 20081* (MO!); East ridge, no elev., 23 Feb 1968 (fr), *J. A. Duke 15261* (MEXU!*, MO!). **Panamá**: [Chepo] El Llano-Cartí road, 5 km N of Pan-American Highway at El Llano, 300 m elev., 10–11 Nov 1973 (imm fr), *M. Nee 7920* (MEXU!*, MO!); El Llano-Cartí road, 16–18.5 km by road N of PanAmerican Hwy, at El Llano, 400–450 m elev., 28 Mar 1974 (fr), *M. Nee & E. Tyson 10983* (CR!, F!*, INPA!*, MO-2 sheets!). **San Blas**: El Llano-Cartí Road, Km 19.1, 350 m elev., 19 Mar 1985 (fr), *G. de Nevers 5184* (INPA!*, MEXU!*, MO!). **Veraguas**: Santa Fe, Valley of Río Dos Bocas along road between Escuela Agricola Alto Piedra and Calovebora, 450–550 m elev., 31 Aug 1974 (♂ fl), *T. B. Croat 27785* (INPA!*, MO!).

### 10. Virola montana

Taxon classificationPlantaeMagnolialesMyristicaceae

D.Santam. sp. nov.

A1E94AB7-4FA5-5164-BE92-C204B2AF880E

urn:lsid:ipni.org:names:77202550-1

Figs 21 , 22A–D

#### Diagnosis.

Species most similar to *Virola guatemalensis*, from which it differs by the mostly caducous (vs. persistent) trichomes on the abaxial leaf surface that have 3–10 branches and are 0.2–0.6 mm long (vs. 3–6 branches 0.05–0.1 mm long), staminate flowers with a shorter filament column (0.6–0.9 mm vs. 1–1.2 mm long) and fruits with a thicker pericarp (3.2–5 mm vs. 0.4–1 [–2.5] mm thick).

#### Type.

Costa Rica. Cartago: Jiménez, Taus de Pejibaye, 900 m elev., 06 Apr 1994 (♂ fl), *E. Lépiz & J. F. Morales 284* (holotype: CR! [201663]; isotypes: CR! [CR1578073], LSU! [0193696, LSU00199100], MO! [5551138, MO280083], USJ! [75968]).

#### Description.

***Tree*** 6–35 m × (4–) 10–50 cm DBH; bark not described. ***Exudate*** described on one occasion as orange in bark, branches and fruits. ***Twigs*** 0.13–0.4 cm thick, angulate to lightly compressed, densely tomentose to puberulent, trichomes dendritic, yellowish, brownish to ferruginous. ***Leaves***: petiole 0.5–1.2 × 0.16–0.3 cm, canaliculate, densely to sparsely pubescent, the trichomes dendritic to irregularly stellate; leaf blades (11.2–) 15–30.5 × (3.9–) 4.5–7.4 cm, oblong-elliptic; adaxial surface on mature leaf blades dark brown when dry, reddish-brown or greyish-blackish, glabrous or with trichomes very sparse and scattered, the surface smooth; abaxial surface pale brown, dark brown or whitish-greyish when dry, sparsely pubescent, but usually densely to sparsely pubescent along the lateral veins and midvein (in new leaves, blades with a dense layer of trichomes that fall readily when touched, covering the entire surface), trichomes dendritic or rarely irregularly stellate, sessile, yellowish to pale brown, with 3–10 branches 0.2–0.6 mm long, caducous; lateral veins (15–) 18–30 per side, (4–) 6–9 veins per 5 cm, 0.5–0.9 (–1.5) cm apart, the same colour as the adaxial surface or sometimes lighter, flat or very slightly sunken on adaxial surface, raised on abaxial surface, straight to slightly arcuate (especially towards the distal part), slightly anastomosing near the margin and without forming a very marked intramarginal vein; tertiary veins usually visible on both surfaces; midvein adaxially flat to slightly canaliculate, glabrous or with scattered trichomes, sometimes densely pubescent at the base, abaxially raised, rounded, densely tomentose (with trichomes that fall very easily to the touch) to glabrescent; base acute to rounded, not revolute, flat; margin flat; apex acute to acuminate. ***Staminate inflorescences*** 3–9 cm long, axillary either at the junction with a leaf or at leafless nodes, axes slightly flattened to irregularly angled, densely tomentose, with trichomes dendritic, brown to yellowish-brown; peduncle 1.7–3.5 × 0.05–0.12 cm; bracts 3–7 × 1.9–4 cm, pubescent on both sides, caducous; terminal fascicles dense, with 7–20 + flowers. ***Staminate flowers*** with the pedicel 1.7–3.4 mm long; receptacle 1.5–3 mm wide; ; perianth (1.6–) 2–2.7 mm, subglobose, yellow, greenish-white or brown, possibly by the indumentum), connate by (0.2–) 0.5–0.8 mm long, abaxial surface pubescent, with brown to ferruginous trichomes, adaxial surface glabrous at the base, sparsely pubescent on the lobes; lobes 3, 1.5–2 × 1–1.4 (–1.8) mm; stamens 3 (–6), the filament column 0.6–0.9 mm long, straight or sometimes slightly thickened at the base and somewhat narrow at the apex, thin, not constricted at the apex; anthers 0.5–0.8 mm long; apiculus ca. 0.07–0.1 mm, inconspicuously apiculate. ***Pistillate inflorescences*** 3.5 cm long, at leafless nodes, with trichomes on the axes similar to those of the staminate inflorescences; peduncle 1.8–2.5 × 1–2 cm; bracts not seen; terminal fascicles of 5–6 flowers. ***Pistillate flowers*** with the pedicel 3.5–4 mm long; perianth 3.5–4.6 mm long, globose to subglobose, pale brown when fresh (possibly by the indument), connate by 1–1.5 mm long, abaxial surface pubescent, with brown trichomes, adaxial surface sparsely pubescent; lobes 3, 2.5–3.5 × 1.5–2.2 mm; gynoecium 2–2.7 × 1.5–1.6 mm, densely pubescent, subglobose, stipitate; stigmatic lobes ca. 0.3 mm, erect. ***Infructescence*** 3–6.2 cm long, with 1 (–2) fruits, peduncle 2–3.3 × 0.19–0.3 cm. ***Fruits*** (2.8–) 3–3.6 × 2–2.5 cm, ovoid-ellipsoid, sessile, densely tomentose to tomentulose, the trichomes dendritic to irregularly stellate, pale brown to ferruginous, the surface commonly rugulose or smooth when dry, the line of dehiscence carinate, the base obtuse, the apex acute, orange, golden or yellowish-brown when fresh; pericarp 3.2–5 mm thick; pedicel 0.7–1 cm long; seed 2.2–2.5 × ca. 1.5 cm, the testa dark brown, almost smooth; aril usually described as red when fresh, yellowish- or reddish-brown when dry, coriaceous, oily in texture, thick, laciniate in narrow bands distally.

**Figure 21.**
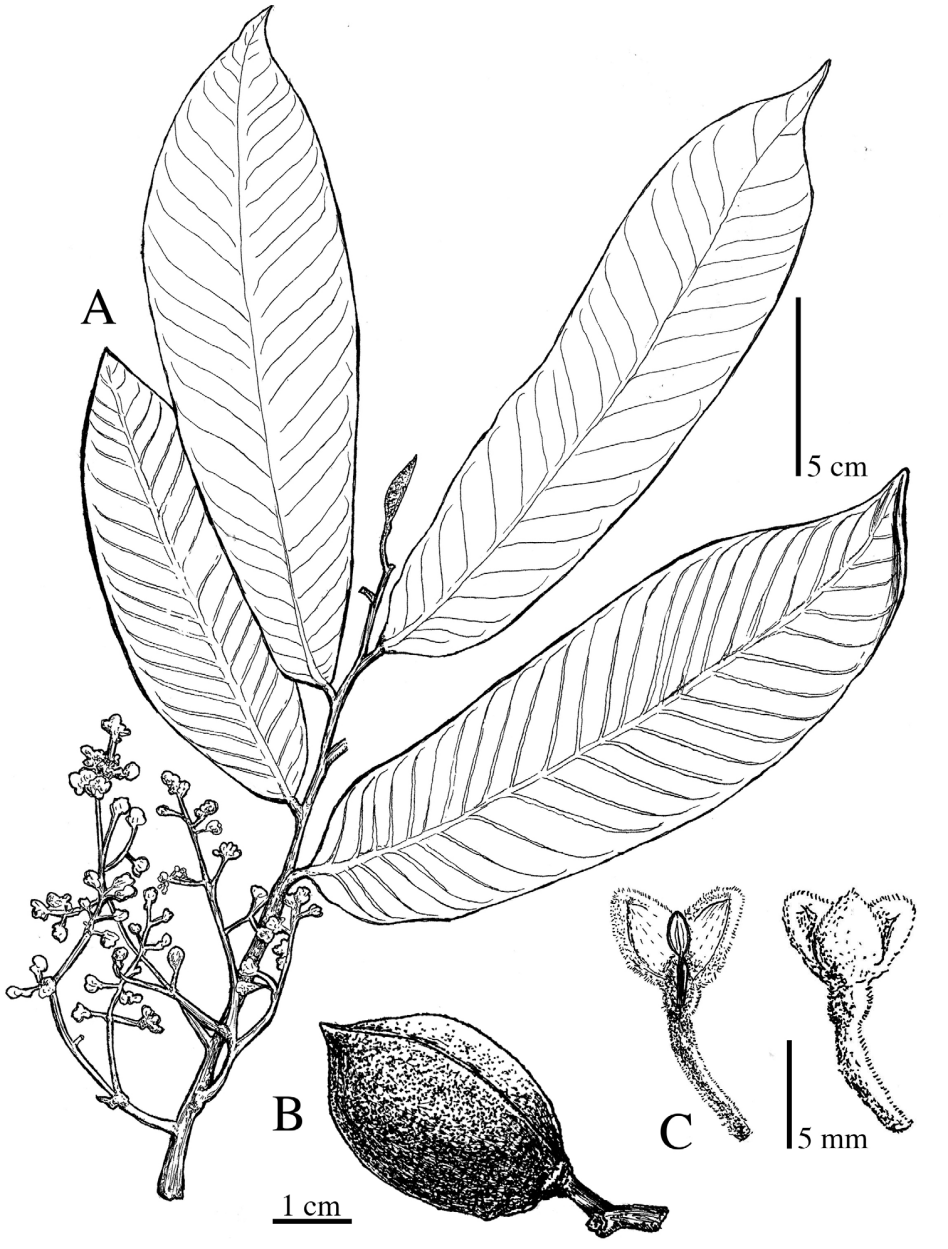
*Virola montana***A** branch with leaves and inflorescences **B** fruit **C** flowers. Drawn by Pedro Juarez based on *G. Herrera et al. 514* (**A–C**), and *R. Lent 3899* (**B**).

#### Distinctive characters.

**Figure 22.**
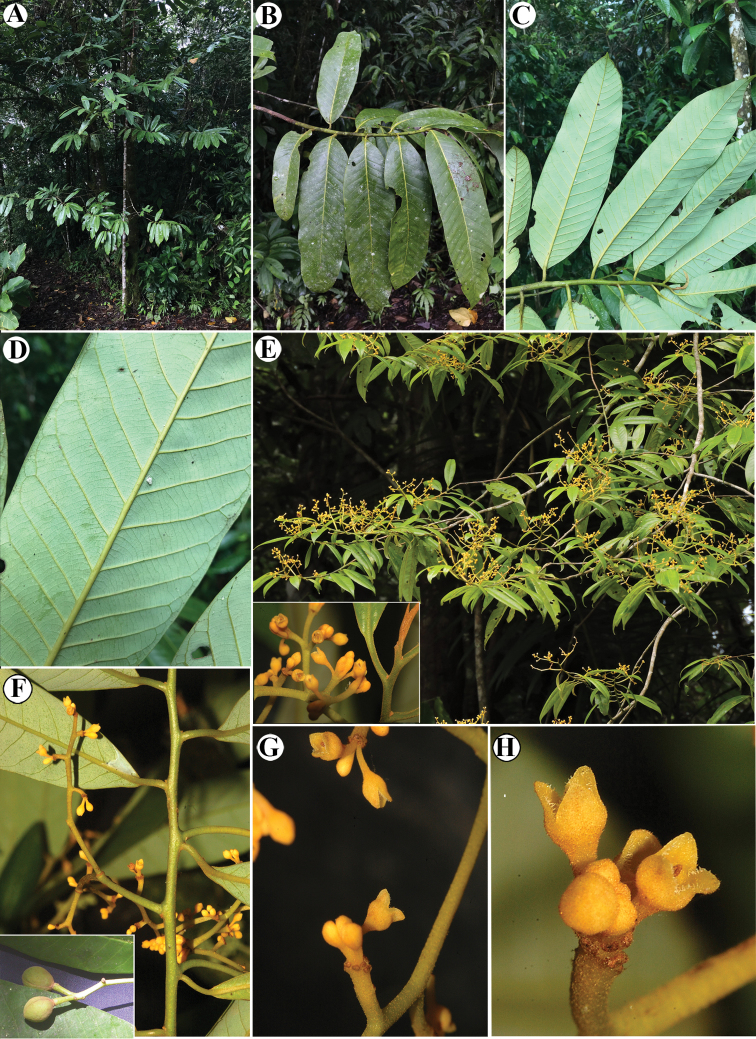
*Virola montana***A** juvenile tree showing branching pattern **B** leaf blades on adaxial side **C** leaf blades on abaxial side **D** venation. *Virola multiflora*. **E** Branch with staminate inflorescences, inset showing twig and flowers **F** twig, leaf blade on abaxial surface and inflorescences, inset showing immature fruit **G** lateral view of a perianth **H** close-up of staminate flowers. Photos by J. Esteban Jiménez (**A–D**); B. Hammel (**E–H**), Indiana Coronado (**F**, inset).

*Virola montana* is recognised by twigs, new leaf undersurfaces, petioles and inflorescences covered in indument of dendritic to irregularly stellate, caducous trichomes, with long branches; on the underside of the leaf, this indument is mainly found on the midvein and the lateral veins (Fig. 3J). Additional traits that distinguish this species include leaf blades with numerous lateral veins [(15–) 18–30 per side; Fig. 22C, D]), the length of the filament column (0.6–0.9 mm long), nearly the same size as the anthers (0.5–0.8 mm long), fruits with thick pericarp that are densely tomentose (Fig. 4H) and carinate in the line of dehiscence (3.2–5 mm), as well as its montane habitat.

#### Etymology.

The specific epithet refers to the montane habitat where the species has been collected.

#### Distribution.

*Virola montana* is known from Costa Rica and Panama, where it has been collected on the Caribbean slope in the provinces of Alajuela, Cartago, Guanacaste, Heredia and Limón in Costa Rica and Bocas del Toro in Panama; it has only been collected on the Pacific slope in Puntarenas (Costa Rica) (Fig. 18D). It has been recorded between 700–2000 m elevation.

#### Preliminary conservation status.

*Virola montana* is of Least Concern following IUCN guidelines. It has both a large EOO (14,606 km^2^) and AOO (104 km^2^) and is known from nineteen localities.

#### Common names.

Costa Rica: fruta dorada.

#### Phenology.

Flowering of *Virola montana* has been recorded in January, March to May, November and December; only two herbarium specimens with pistillate flowers were seen. Fruits have been collected in March and June to December.

#### Field characters.

Leaf blades are lustrous, dark green above and whitish or silver below. Flowers have yellow, cream, greenish-white or brown perianth. Mature fruits are yellow, pale brown, brown yellow or orange. The seed is brown with a red aril.

#### Discussion.

As far as we know, the first specimen of this species was collected 115 years ago by Henri F. Pittier (1857–1950) in the mountains of El Rosario de Orosi, Cartago, Costa Rica (*H. Pittier 16628*, NY-2 sheets!*). Paul C. Standley treated this specimen as *V. koschnyi* in “Flora of Costa Rica” (Standley 1937). Shortly after, Albert C. Smith and Roger P. Wodehouse (1938) included the Pittier specimen under *V. guatemalensis* and this remains the name that is most frequently misattributed to specimens of *V. montana* (e.g. Standley and Steyermark 1946; Jiménez 2007). However, *V. guatemalensis*, as interpreted here, is restricted to northern Mesoamerica (Mexico, Guatemala and Honduras). In addition to the differences given in the diagnosis, the *V. montana* can be distinguished morphologically from *V. guatemalensis* by the differences summarised in Table 6. Although it is difficult to quantify, specimens of *V. montana* commonly have twigs, petioles and inflorescences covered with more trichomes with long branches (see Fig. 3F, J).

**Table 6.** Morphological differences between *Virola montana* and *V. guatemalensis*.

Character	*V. montana*	*V. guatemalensis*
Leaf blades size	(11.2–) 15–30.5 × (3.9–) 4.5–7.4 cm	12.3–17.5 (–24.1) × (2.4–) 3.8–5.5 (–8.9) cm
Indument and trichomes on abaxial surface	Sparsely pubescent, but usually densely to sparsely pubescent primarily along the lateral veins and midvein; trichomes with 3–10 branches, the branches 0.2–0.6 mm long, caducous (Fig. 3J)	Puberulent, trichomes over the entire surface; trichomes with 3–6 branches, the branches 0.05–0.1 mm long, persistent (Fig. 3F)
Lateral veins	(15–) 18–30 per side, on abaxial side raised (Figs 8J, 22D)	13–21 per side, on abaxial side slightly raised or flat (Figs 8F, 10F)
Filament column	0.6–0.9 mm long	1–1.2 mm long
Pericarp thickness and presence of a carina	3.2–5 mm thick, carinate	0.4–1 (–2.5) mm thick, smooth or slightly carinate

In addition to *Virola guatemalensis*, herbarium specimens of this new species have been determined as three other species: *V. koschnyi*, *V. sebifera* and *V. surinamensis* (here treated as *V. nobilis*). Vegetatively, *V. montana* can be distinguished from these species by its mature leaves that are abaxially sparsely pubescent (vs. covering the entire surface of the leaf blades abaxially). The first two species are distinguished by pediculate trichomes on abaxial surface of leaf blades (vs. sessile in *V. montana*, these primarily present in young leaves). Finally, *V. nobilis* has trichomes with short branches [0.05–0.1 mm (Fig. 3L) vs. 0.2–0.6 mm long (Fig. 3J)], tends to have more lateral veins [25–34 vs. (15–) 18–30 per side] and has a preference for lower elevation habitats [0–500 (–1300) m vs. 700–2000 m elevation].

Based on the number of herbarium specimens collected, *Virola montana* is the most common montane species of *Virola* in southern Mesoamerica and it is usually the only species where it occurs. However, three fruiting specimens of *Virola* sp., represented by *R. Aguilar et al. 4327* (CR!, MO!), *G. McPherson 8723* (MO!) and *G. McPherson 10514* (MO!), have been collected in the general vicinity of *V. montana* in its preferred elevational range (1050–1500 m). These specimens clearly differ from *V. montana* in their small leaf blades [10.5–12.5 × 2.3–3.5 cm vs. (11.2–) 15–30.5 × (3.9–) 4.5–7.4 cm] with trichomes on the abaxial surface that have short branches (0.08–0.1 mm vs. 0.2–0.6 mm long) and cover the entire surface (vs. sparsely pubescent, but usually densely to sparsely pubescent along the lateral veins and midvein in *V. montana*). These specimens share characteristics with two lowland species of *Virola*: *V. nobilis* and *V. fosteri*, including sessile, stellate trichomes on the abaxial surface of leaves. Additionally, these three specimens share their small leaves with *V. fosteri*, though not *V. nobilis* (9–17.2 [–27.5] × 2.5–5 [–4.7–7.1] cm vs. 10.5–12.5 × 2.3–3.5 cm). Further, the fruits of *V. fosteri* are smaller (1.5–2.3 × 1.2–1.8 cm vs. 2.8–3.2 × 2.2–2.7 cm). Additional collection and study is necessary to determine the identity of these specimens.

#### Specimens examined.

**Costa Rica. Alajuela**: Reserva Biológica Monteverde, Río Peñas Blancas, parcela de Badilla, 800 m elev., 23 Oct 1988 (fr), *E. Bello 470* (CR-2 sheets!, INPA!*, LSU!, MO!); Reserva Biológica Monteverde, Parcela de Jesús Rojas, 850–900 m elev., 05 May 1989 (♂ fl), *E. Bello 855* (CR-2 sheets!, INPA!*, MEXU!*, MO!); Reserva Biológica Monteverde, Ref. Aleman, 900 m elev., 10 Nov 1993 (♂ fl), *E. Bello 5404* (CR!, LSU!, MO!); Reserva Forestal de San Ramón, 850–1150 m elev., 12–14 Mar 1987 (♂ fl), *W. Burger et al. 12152* (CR!, F!*); San Ramón, Reserva Biológica Alberto Brenes, Estación San Lorencito, 900 m elev., 03 May 2000 (♂ fl), *K. Caballero 2* (CR-2 sheets!, MO!); Reserva Forestal de San Ramón, 900–1200 m elev., 12–15 Mar 1987 (♂ fl), *J. Gómez-Laurito 11446* (CR!, USJ!); Reserva Forestal de San Ramón, 900–1200 m elev., 16–19 Apr 1987 (♂ fl), *J. Gómez-Laurito 11484* (CR!, F-2 sheets!*); Reserva Forestal de San Ramón, Estación Río San Lorencito, 850–900 m elev., 18 Oct 1989 (fr), *J. Gómez-Laurito et al. 11846* (CR!, MEXU!*, MO!, USJ!); La Balsa, 1100 m elev., 02 Jul 1990 (st), *J. Gómez-Laurito & J. A. López 11970* (USJ!); Reserva Biológica Monteverde, Río Peñas Blancas, 850 m elev., 31 Mar 1987 (♂ fl), *W. Haber & E. Bello 6978* (CR!, F!*, INPA!*, MEXU!*, MO!); Reserva Biológica Monteverde, finca Wilson Salazar, 800 m elev., 07 Nov 1987 (fr), *W. Haber & E. Cruz 7698* (CR!, INPA!*, MO!, USJ!); Río San Lorenzito, 800–1000 m elev., 30 Mar 1987 (♂ fl), *G. Herrera et al. 514* (CR!, INPA!*, LSU!, MO!); La Fortuna, Finca El Jilguero, 1160 m elev., 20 Nov 1992 (fr), *G. Herrera 5544* (CR!); 2 km N.E of La Balsa de San Ramón, 900 m elev., 26 Sep 1976 (fr), *R. Lent 3899* (CR-3 sheets!, INPA!*, MARY!*, MEXU!*, MO!, PMA!*, U!*); Reserva Forestal San Ramón, Estación Río San Lorenzo, sendero al Volcán Muerto, 1100 m elev., 26 Apr 1993 (♀ fl), *F. Quesada 20* (CR!, LSU!, MO!); Lago Coter [Cote], Hotel Eco-Lodge, 700 m elev., 09 Apr 1997 (♂ fl), *J. Rivera & B. Petruzzi 2933* (CR!). **Cartago**: Taus, faldas del Cerro Alto El Humo, 900–1200 m elev., 06 Apr 1994 (♂ fl), *J. F. Morales & E. Lépiz 2654* (CR!, MO!); El Rosario de Orosi, no date, Jan 1903 (fr), 1120 m elev., *H. Pittier 16628* (NY-2 sheets!*); Turrialba, Monumento Nacional Guayabo, Santa Teresita, sobre los ríos Guayabo, Lajas y Torito, 700–1800 m elev., 08 May 1992 (♂ fl), *G. Rivera 1659* (CR!, MO!, USJ!); límite Sur del Monumento Nacional Guayabo, sector Las Ventanas, 1100 m elev., 07 Jul 1992 (fr), *G. Rivera 1910* (CR!, F!*, MO!, USJ!); [Paraíso] 5 km N.W of Río Grande de Orosi at Tapanti, 1300 m elev., 27 May 1976 (♂ fl), *J. Utley & K. Utley 5054* (CR-2 sheets!, MEXU-2 sheets!*, MO-2 sheets!). **Guanacaste**: San Gerardo, 1000 m elev., 21 Nov 1998 (fr), *E. Bello 553* (CR!, INPA!*, MO!). **Heredia**: Sarapiqui, Parque Nacional Braulio Carrillo, puesto El Ceibo, 750 m elev., 05 Mar 1994 (st), *B. Boyle et al. 2938* (MO!); Cariblanco, 800 m elev., n.d. Mar 1950 (fl bud), *J. León 2368* (USJ!); Cariblanco, 800 m elev., 10 Aug 1950 (fr), *LRH* [*L. R. Holdridge*] *2618* (USJ!). **Limón**: Talamanca, Bratsi, Alto Lari, Kivut, 1300–1500 m elev., 15 Mar 1992 (fr), *R. Aguilar & H. Schmidt 1131* (CR!, MO!). **Puntarenas**: Coto Brus, Zona Protectora Las Tablas, sitios Las Juntas, 1500 m elev., 03 Jul 1999 (fr), *E. Alfaro 2357* (CR-2 sheets!, MO!); [Coto Brus], Las Tablas, río Cotoncito, [1300–1500 m elev.], 10 Dec 1983 (fr), *I. Chacón et al. 1819* (INPA!*, LSU-2 sheets!, MO!); Parque Internacional La Amistad, Estación Pittier, 1650 m elev., 28 Jan 1995 (♀ fl), *M. Chinchilla & IV curso de paratoxónomos 3* (CR-2 sheets!, LSU!, MO!); Zona Protectora Las Tablas, 2000 m elev., 01 Sep 1992 (fr), *A. Fernández 354* (CR!); Fila Tigre, SE of Las Alturas, 1350–1450 m elev., 29 Aug 1983 (fr), *G. Davidse 24178* (CR!, INPA!*, MEXU!*, MO!); Parque Internacional La Amistad, Santa María de Pittier, 1700 m elev., 13 Jun 1995 (fr), *J. González 823* (CR-2 sheets!); Coto Brus, Zona Protectora Las Tablas, Hacienda La Amistad, 1600–2000 m elev., 28 Dec 2003 (♂ fl), *R. Kriebel et al. 4174* (CR-2!, MO!). **Panama. Bocas del Toro**: Bocas del Toro-Chiriquí border above Fortuna Dam, 1200 m elev., 04 Dec 1985 (imm fr), *G. McPherson 7756* (INPA!*, MO!, PMA!*).

### 11. Virola multiflora

Taxon classificationPlantaeMagnolialesMyristicaceae

(Standl.) A. C. Sm.

04AE7760-CAC2-58C9-93F6-C8A0A4F5374C

Fig. 22E–H

Virola multiflora (Standl.) A. C. Sm. Brittonia 2: 499. 1938.
Dialyanthera multiflora Standl. Publ. Field Mus. Nat. Hist., Bot. Ser. 8: 12. 1930. Type. [Belize] British Honduras. In jungle, Stann Creek Railway, alt. 30 m, 16 Jul 1929 [♂ fl], *W. A. Schipp 279* (holotype: F!*; isotypes: A!*, BM!*, G-2 sheets!*, GH!*, K!*, MO!, S!*).
Virola brachycarpa Standl. Publ. Field Mus. Nat. Hist., Bot. Ser. 11: 131. 1932. Type. [Belize] British Honduras. Stann Creek Valley, 13 Jan 1932 [fr], J. A. Burns 20 (holotype: F!*; isotypes: BKL!*, G!*, US!*, WIS!*).

#### Distinctive characters.

*Virola multiflora* is recognised by its usually small and narrow leaf blades [5.5–15.5 × 1.5–3.6 (–4.8) cm] with an inconspicuously pubescent abaxial surface with stellate and sessile trichomes (Fig. 3K), lateral veins that are not very prominent (Fig. 8K) and long and thin petioles [0.5–0.9 (–1.1) cm long]; staminate flowers with the filament column longer (0.7–1 mm long) than the anthers (0.3–0.6 mm long); and for its small fruits [1.3–1.9 × 0.9–1.2 (–1.4) cm] (Fig. 4J) with the pericarp 0.7–1 mm thick.

#### Distribution.

*Virola multiflora* is known from Belize (Cayo, Stann Creek and Toledo), Honduras (Gracias a Dios), Nicaragua (Atlántico Norte, Atlántico Sur, Jinotega, Matagalpa and Río San Juan) and Costa Rica (Alajuela, Cartago, Heredia and Limón) (Fig. 18E). Throughout its range in Mesoamerica, it is only known from the Caribbean slope. It has been recorded from between 0–650 (–1400) m elevation.

While *V. multiflora* is not documented from Guatemala in herbaria, Standley and Steyermark (1946) postulated that the species is to be expected to occur in Izabal (Guatemala) and Jiménez (2007) attributes it to that country. Conversely, while it is mentioned as occurring in Peru by Jiménez (2007), specimens could not be located. The Peruvian specimen attributed to *V. multiflora* (*R. Vásquez & C. Grández 17507*, MO!; fr) in Vásquez M. et al. (2018) corresponds to a species related to *V. multinervia*. Finally, the report presented in the *Nuevo Catálogo de la Flora Vascular de Venezuela* (Rodrigues 2008) is errorenous. Based on this evidence, *V. multiflora* is considered as restricted to Mesoamerica.

#### Common names.

Belize: banak, bastard banak. Honduras: asang banak, bának, banak almuk, báhanak luhpia, sangre, sebo álmut, sebo negro. Nicaragua: conchillo, samo. Costa Rica: fruta dorada.

#### Phenology.

Flowering of *Virola multiflora* has been recorded in March to August, with a noted peak in July; just one herbarium specimen with pistillate flowers was seen and it is from Nicaragua. Fruits were collected in December through April.

#### Field characters.

Plants are trees between 6–30 m tall and 17–35 cm DBH. Bark exudes latex red. The leaf blades are sometimes whitish abaxially. The flowers usually have yellow, golden or orange perianth. The mature fruit is yellow or orange with a red aril.

#### Notes.

We were not able to locate any Panamanian specimens of *V. multiflora*: all fertile Panamanian specimens identified as *V. multiflora* that the first author has studied are actually *V. fosteri*. However, *V. multiflora* is to be expected in Panama because it occurs in physiognomically similar forests in Costa Rica near the border. The similarities and differences between *V. multiflora* and *V. fosteri* (which is formally described above) are discussed under the latter species.

#### Selected specimens seen.

**Belize. Cayo**: Hummingbird Highway south of Belmopan, 200–300 ft [60–90 m] elev., 26 Jun 1973 (♂ fl), *A. Gentry 8615* (MO!). **Stann Creek**: Big Eddy Ridge, [50–200 m elev.], 12 May 1940 (fl bud), *P. H. Gentle 3333* (MO!); Cockscomb Basin Wildlife Sanctuary, 80 m elev., 17 Jan 2007 (fr), *P. Hechenleitner 289* (MO!). **Toledo**: Between Rancho Chico and Cockscomb, no elev., 26 Mar 1943 (fl bud), *P. H. Gentle 4342* (MO!); Big Creek, 100 ft [30 m] elev., 02 Dec 1931 (fl bud), *W. A. Schipp 858* (MO-2 sheets!). **Honduras. Gracias a Dios**: 1 km al sureste de Krausirpe, pie de montaña de Wimpi, 90 m elev., 18 Feb 1994 (fr), *P. R. House 1888* (MO!); transecto Botánico Cerro Krautara, 120 m elev., 16 Mar 1995 (♂ fl), *P. R. House 2308* (MO!). **Nicaragua. Atlántico Norte**: El Salto, along Río Pis Pis, 100 m elev., 27 Feb 1979 (fr), *J. J. Pipoly 3615* (MO!); 13 km above Kururia on road to San Jerónimo, 200 m elev., 02 Mar 1979 (♂ fl), *J. J. Pipoly 3846* (MO!). **Atlántico Sur**: Nueva Guinea, Reserva Indio-Maíz, 200–300 m elev., 05 Jan 1999 (fr), *R. Rueda et al. 9857* (MO!); 2 km de Colonia Serrano, Comarca El Escobillo, 80–100 m elev., 28 Jul 1982 (♀ fl), *J. C. Sandino 3302* (MO!). **Jinotega**: Cua Bocay, Reserva de Bosawas, 278 m elev., 03 Oct 2005 (imm fr), *I. Coronado et al. 2306* (MO!). **Matagalpa**: Río Blanco, Reserva Natural Cerro Musún, 500–1400 m elev., 15 Jul 2000 (♂ fl), *R. Rueda & O. Caballero 14294* (MO!). **Río San Juan**: 1 km al NW del Río Santa Cruz, 60 m elev., 22 Feb 1984 (fr), *P. P. Moreno 23253* (MO!); sobre el río Sábalo, 40 m elev., 07 Jul 1984 (♂ fl), *P. P. Moreno & W. Robleto 25984* (MO!); Boca Negra, 120 m elev., 14 Feb 1990 (fr), *P. P. Moreno 27266* (MO!, P!*); Estación Biológica Bartola, 50–100 m elev., 26 Jul 1998 (♂ fl), *R. Rueda et al. 8196* (MO!); Estación Experimental La Lupe, 100 m elev., 22 Nov. 2000 (fr), *R. Rueda & W. Velásques 15004* (MO!). **Costa Rica. Alajuela**: Corredor Fronterizo Costa Rica-Nicaragua, 2 km antes de Boca San Carlos, 0–100 m elev., 15 Mar 2004 (♂ fl), *J. F. Morales 10322* (CR!, MO!). **Cartago**: 24 km northeast of Turrialba on highway to Limón, 450–525 m elev., 10 May 1983 (♂ fl), *R. Liesner et al. 15390* (MO!). **Heredia**: Puerto Viejo de Sarapiquí, camino a Cerros Sardinal, Caño Negro, 71 m elev., 25 Jul 2016 (♂ fl), *B. Hammel & I. Pérez 27148* (CR!); along Guácimo ridge trail, 315 m elev., 18 Jan 1983 (fr), *G. S. Hartshorn 2553* (CR!, MO-2 sheets!). **Limón**: Siquirres, Fila Mirador, camino a Las Brisas de Pacuarito, 400 m elev., 13 Feb 2000 (fr), *M. Blanco & A. Vega 1460* (USJ!); Bajo Telire, 400–600 m elev., n.d. July 1984 (♂ fl), *L. D. Gómez 24151* (CR!, MO!); Talamanca, Finca La Culebra, a 1.5 km de camino Bribrí, 0–200 m elev., 26 Oct 1992 (fl), *J. Gómez-Laurito & H. Gómez 12340* (USJ!); Matina, intersección de Río Barbilla y Quebrada Cañabral, 100–200 m elev., 11 Oct 1988 (imm fr), *G. Herrera 2167* (MO!); Parque Nacional Tortuguero, Lomas de Sierpe, 100 m elev., 15 Aug 1988 (♂ fl), *R. Robles et al. 2063* (MO!); Sixaola, San Miguel, camino entre Fila Tsipubeta y Cerro Mirador, 200–300 m elev., 12 Nov 1999 (fl), *O. Valverde & S. Hernández 1237* (CR!, USJ!).

### 12. Virola nobilis

Taxon classificationPlantaeMagnolialesMyristicaceae

A. C. Sm.

7C429EDB-2AC8-52BD-BB9E-DF297C84E0CF

Figs 20C–G , 24B

Virola nobilis A. C. Sm. Brittonia 2: 490. 1938. Type. Panama. Canal Zone, Barro Colorado Island, 07 Jan. 1932 [imm fr], [R. H.] Wetmore, [E. C.] Abbe & [O. E.] Shattuck 155 (holotype: GH!*; isotypes: A!*, F!*, MO!).

#### Distinctive characters.

*Virola nobilis* is recognised by its narrow, oblong leaf blades (9–17.2 [–27.5] × 2.5–5 [–4.7–7.1] cm) with numerous lateral veins (20–30 [25–32] per side) corresponding to 8–11 (5–7 [–9]) veins per 5 cm, as well the stellate, tertiary veins that are slightly sunken above (Fig. 3L) and sessile trichomes scattered on underside of the leaf (Fig. 3L); the staminate flowers with the filament column longer (0.8–1.1 [1.2–1.3] mm long) than the anthers (0.6–0.8 mm long); and usually ellipsoid or ovoid fruits (2.3–2.7 × 2.2–2.4 cm [2.1–3.1 × 1.6–2.5 cm]) (Fig. 4N) with thick pericarp (2.3–3.5 mm [2.5–4.2 mm]) and aril.

#### Distribution.

*Virola nobilis* is known from the Pacific slope of Costa Rica (Puntarenas, San José) and the Caribbean slope of Panama (Bocas del Toro, Colón, Panamá, San Blas). It is recorded from 0–400 (–1300) m elevation (Fig. 18F). Jiménez (2007) reported this species (as *V. surinamensis*) from just one locality (Chitaría) on the Caribbean slope in Costa Rica; however, we have not examined the specimen (*Poveda 144*, CR).

#### Common names.

Costa Rica: Fruta dorada. Panama: bogamani, coton cuinur gia, sabdurgia (Kuna name).

#### Phenology.

Flowers have been collected in January to April, July, August, November and December and fruits in almost all months except October.

#### Field characters.

Plants are large trees between (5–) 15–40 m tall and 20–70 cm DBH. The trunk is straight, usually with conspicuous buttresses and does not begin to branch until it reaches a great height. The bark is reddish and releases red exudate when damaged. The leaves are whitish with inconspicuous trichomes on the abaxial surface. Flowers have yellow perianth. The mature fruit is yellow to orange with a red aril.

#### Discussion.

**Figure 23.**
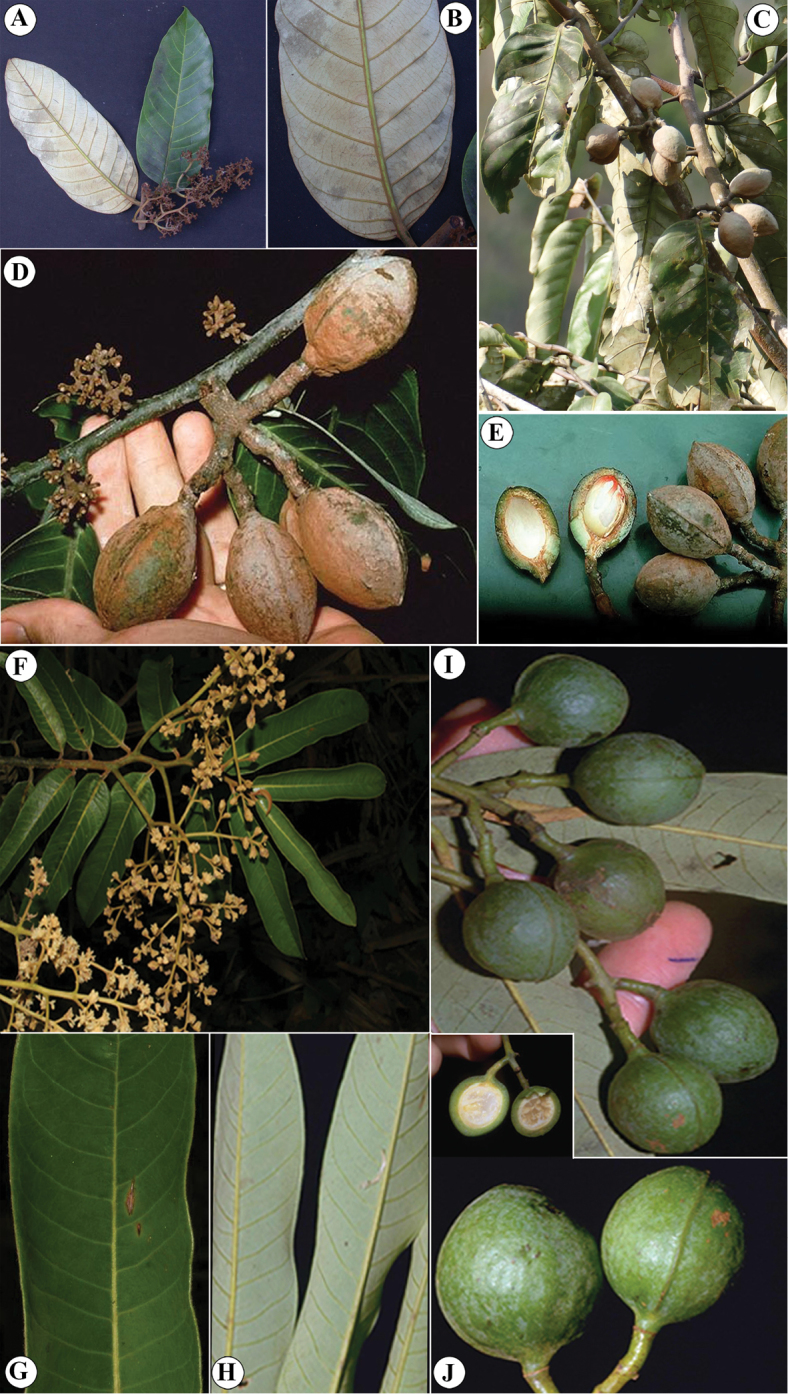
*Virola otobifolia***A** branch with leaf blades showing both surfaces and inflorescences **B** leaf blade, venation and base on abaxial surface **C** infructescence **D** fruits **E** detail of fruit, showing an aril of an immature fruit. *Virola surinamensis*. **F** Branch with inflorescences **G, H** leaf blade on adaxial (**G**) and abaxial surface (**H**). **I** Infructescence **J** detail of fruits, including a longitudinal section of an immature fruit. Photos by Rolando Pérez (**A, B** from https://stricollections.org/portal/index.php); Jerry Harrison (**C**), Alwyn H. Gentry (**D, E** from http://www.tropicos.org) and John P. Janovec (**F–J** from http://atrium.andesamazon.org/).

**Figure 24.**
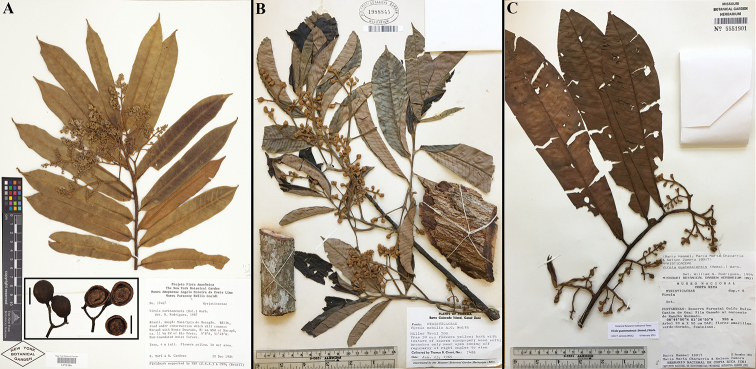
Comparisons of *Virola surinamensis* with collections of *V. nobilis***A***V. surinamensis* (*S. Mori & R. Cardoso 17467*, NY; inset showing fruits from *W. E. Broadway 5264a*, MO) **B***V. nobilis* (*T. B. Croat 7488*, MO) **C***V. nobilis* from Costa Rica (B. *Hammel 18017*, MO). **A**, image courtesy of New York Botanical Garden.

This species has usually been treated, identified and included as a synonym of *Virola surinamensis* (e.g. Gentry 1975; Croat 1978; Correa et al. 2004; Jiménez 2007), which has similar leaf morphology (i.e. number of lateral veins, stellate and sessile trichomes scattered on the abaxial surface of the leave blades). However, *Virola surinamensis* (Figs 23G–J, 24A) is characterised by having a shorter perianth that is also somewhat fleshy to submembranous and smaller fruits that are also ovoid to subglobose, glabrescent and with a thin pericarp (Figs 23I–J, 24A inset; also see illustration in Rodrigues 1972, 1980). Although both species may have similarly-sized leaves, they tend to be smaller in *V. surinamensis*, which also has inflorescence axes that are longer and with many more flowers (Figs 23F, 24A).

#### Notes.

**Figure 25.**
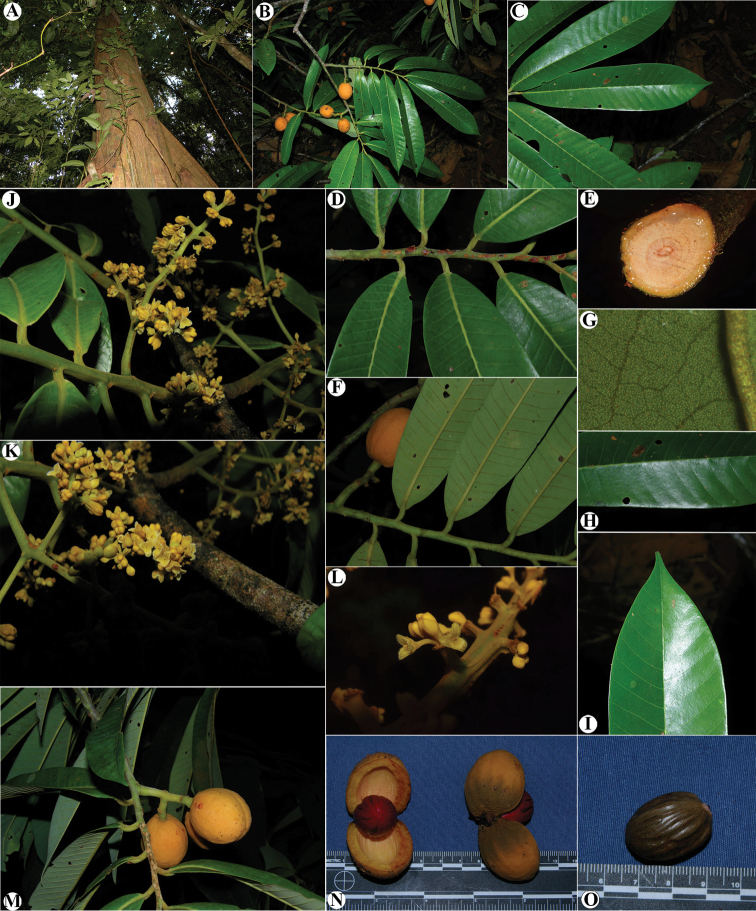
Virola cf. nobilis from the Osa Peninsula, Costa Rica **A** trunk **B** branch with fruits **C** leaf blades on adaxial surface **D** twig, petiole and leaf base on adaxial surface **E** exudate on a twig **F, G** leaf blades on abaxial surface and trichomes (**G**). **H** Leaf margin **I** leaf apex **J** staminate inflorescences **K** detail of staminate inflorescences **L** detail of staminate flower **M** branch with fruits **N** fruits **O** seed. Photos by Reinaldo Aguilar.

*Virola nobilis* exhibits complex variation that requires more fieldwork, ideally in combination with molecular phylogenetic analysis, to clarify species boundaries. For example, specimens from the Pacific slope in Costa Rica (see specimens cited below and Figs 24C, 25) are included, with reservation, under *V. nobilis*. These specimens differ from other collections of *V. nobilis*, including the type and additional specimens from Barro Colorado Island (the type locality), in having wider leaf blades, fewer lateral veins, tertiary veins that are less inconspicuous below (Fig. 3L, O), a longer filament column and ovoid fruits with obtuse to rounded apices (Fig. 4N, O). These specimens also resemble *V. reidii* Little, a species from Colombia and Ecuador (Jaramillo et al. 2004). The measurements presented in square brackets [] correspond with the Costa Rican material under question.

Other material that potentially belongs to *V. nobilis* includes a few collections from Panama, including both infertile and fruting specimens from El Llano Cartí and others from Cerro Jefe (e.g. *G. de Nevers & H. Herrera 4333*, imm fr, MO!; *R. L. Liesner 657*, fr, MO!; *E. L. Tyson et al. 3353*, fr, MO!). These differ from *V. nobilis* by having the abaxial surface of the leaf blade covered by a dense and a very inconspicuous layer of stellate, sessile and yellowish trichomes and fruits with a more or less smooth surface and that are not carinate in the line of dehiscence. Finally, *R. Aguilar 3408* (CR-3 sheets!, MO!) from the Osa Peninsula of Costa Rica and *G. McPherson 11474* (MO!; fr) from Panama differ from the species concept that we adopt in having trichomes on the abaxial leaf side with long branches (ca. 0.2 mm long) that are often shortly pediculate; and small fruits (ca. 2.1 × 1.6–1.7) that are ovoid to subglobose and apiculate at the apex.

#### Selected specimens.

**Costa Rica. Puntarenas**: Golfito, camino a Piro, finca de Adrian, 50 m elev., 16 Apr 2005 (fr), *R. Aguilar & X. Cornejo 9739* (MO!); Osa, Punta Pargos, 0 m elev., 17 Apr 2008 (fr), *R. Aguilar 11186* (MO!, USJ!); Osa, Rancho Quemado, siguiendo el nuevo camino a Drake, 400 m elev., 20 Jun 1990 (fr), *G. Herrera 4222* (MO!); Trocha de La Tarde rd. 10 km SW of La Palma, 150–200 m elev., 28 Apr 1988 (fr), *B. Hammel & R. Robles 16722* (MO!); Osa, Fila Ganado, 350 m elev., 15 Dec 1990 (♂ fl), *B. Hammel et al. 18017* (CR-2 sheets!, MO!); Osa, Fila Ganado hasta Guerra, 1–300 m elev., 28 Mar 1991 (fr), *B. Hammel et al. 18170* (MO!). **San José**: Tarrazú, 900–1300 m elev., 19 Aug 1997 (fr), *O. Valverde & A. Estrada 135* (CR!). **Panama. Bocas del Toro**: North of Fortuna Dam, on road to Chiriquí Grande, 500 m elev., 18 Jan 1986 (fr), *G. McPherson 8098* (MO!). **Colón**: Carretera Gatún-Piñas, 0–50 m elev., 26 Jul 1994 (fr), *C. Galdames et al. 1415* (CR!, MO!). **Panamá**: Barro Colorado Island, [10– 100 m elev.], 23 Jan 1969 (fl bud), *T. B. Croat 7488* (MO!); ibid, 22 Feb 1969 (fr), *T. B. Croat 8090* (MO!); El Llano Cartí, 1100–1200 m [335–365 m] elev., 28 Dec 1974 (fr), *S. Mori & J. Kallunki 4151* (CR!). **San Blas**: El Llano-Cartí, 100–350 m elev., 03 Nov 1985 (imm fr), *G. de Nevers et al. 6172* (MO!).

### 13. Virola otobifolia

Taxon classificationPlantaeMagnolialesMyristicaceae

D.Santam. sp. nov.

8269F333-FCF1-547F-9629-0F179013CC7F

urn:lsid:ipni.org:names:77202553-1

**Figure 26.**
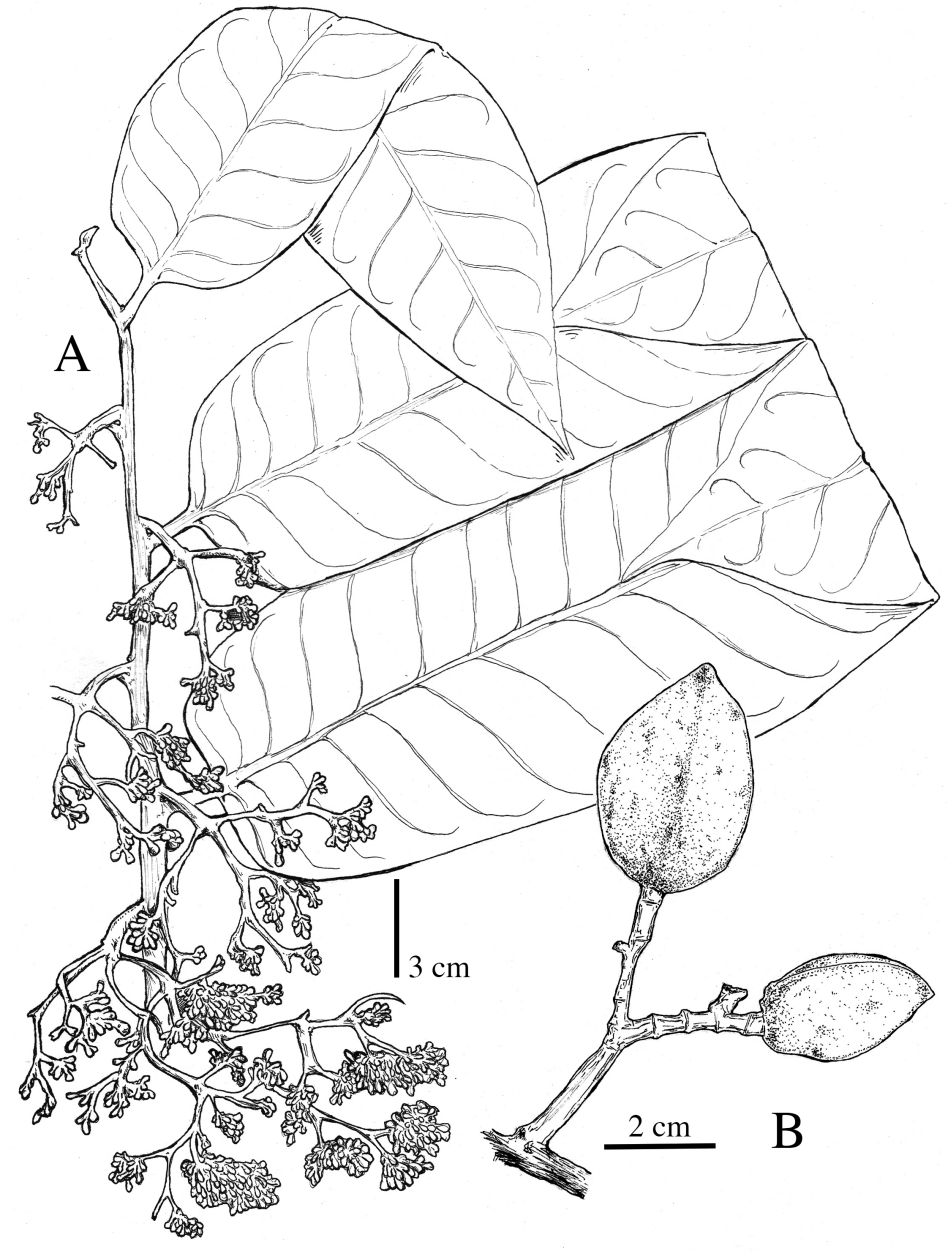
*Virola otobifolia***A** branch with leaves and inflorescences **B** fruits. Drawn by Pedro Juarez, based on *S. Mori & J. Kallunki 5542* (**A**) and *J. P. Folsom 1440* (**B**).

Figs 7D , 23A–E , 26

#### Diagnosis.

Species most similar to *Virola macrocarpa* in having leaf blades that are discolorous abaxially with stellate, sessile trichomes with a reddish central portion and similar number of lateral veins (10–16) that are not densely spaced. However, it differs in its leaves with dense, but inconspicuously pubescent leaf undersides (vs. inconspicuously puberulent) with 2–4 veins per 5 cm that are 1.7–3 cm apart (vs. 4–5 veins per 5 cm, 0.8–1.5 cm apart) and fruits that are (2.7–) 3.5–4.5 × (1.9–) 2.3–2.9 cm, densely tomentose with persistent trichomes, a rugose surface when dry and a line of dehiscence that is conspicuously carinate (vs. 2.7–3.3 × 2–2.3 cm, tomentellous, with caducous, dust-like trichomes, a smooth surface when dry, the line of dehiscence slightly carinate), as well as the production of thicker pericarp [(2.7–) 3–4.7 mm vs. 1.8–3 mm thick].

#### Type.

Panama. [Panamá: Chepo], El Llano Cartí road 6.9 km N of Panamerican Highway, 350–500 m elev., 23 Jan 1977 (fr), *J. P. Folsom 1440* (holotype: MO-2 sheets! [2601823, MO117643, 5551400, MO299335]; isotype: PMA!* [101093, PMA111119]).

#### Description.

***Tree*** 6–30 m × 24.9–37.3 cm DBH; bark not described. ***Exudate*** from the trunk sometimes described as red, watery-red, or black-reddish. ***Twigs*** 0.23–0.34 cm, terete to slightly flattened, glabrescent to puberulent, trichomes stellate to irregularly stellate, ferruginous to greyish. ***Leaves***: petiole (1–) 1.3–2 × 0.19–0.44 cm, flat to very slightly canaliculate, tomentose, the trichomes stellate; leaf blades (14–) 18.2–42.5 × (4.1–) 7.3–14.2 cm, oblong to elliptic; adaxial surface of mature leaf blades brown (sometimes shining) when dry, glabrous or sometimes with scattered stellate trichomes (especially along the veins), the surface smooth; abaxial surface when drying whitish-greyish or sometimes very light brown, dense but inconspicuously pubescent, trichomes stellate, sessile, the central part of the trichome usually reddish, contrasting in colour with the hyaline branches to reddish-clear, with 4–11 branches, the branches ± 0.01–0.05 mm, persistent; lateral veins 10–16 per side, 2–4 veins per 5 cm, 1.7–3 cm apart, the same colour as the adaxial surface, on adaxial surface flat to slightly sunken, on abaxial surface slightly elevated, arcuate, slightly anastomosing near the margin and without forming a very marked intramarginal vein; tertiary veins barely visible on both surfaces; midvein adaxially elevated, but submerged in a channel, abaxially raised, triangular to rounded, tomentose; base acute to obtuse, sometimes slightly cordate, not revolute, flat; margin flat to slightly revolute; apex acuminate. ***Staminate inflorescences*** 3.5–9.5 cm long, axillary either at a leaf or at a leafless node, axes flattened or irregularly flattened, tomentose, with trichomes irregularly stellate to irregularly dendritic, brown to ferruginous; peduncle 1.4–2.2 × 0.22–0.34 cm long; bracts not seen; terminal fascicles lax, with 5–13 flowers. ***Staminate flower*** with the pedicel 1.5–2 mm long; receptacle 0.7–1.5 mm wide; ; perianth 2.5–2.8 mm long, infundibuliform, yellow or brown when fresh, connate by 0.9–1.5 mm long, abaxial surface pubescent with brown trichomes, adaxial surface glabrous or with few trichomes close to the base; lobes 3 (4), 1.2–1.5 × 0.9–1.1 mm; stamens 3 (–6), the filament column 0.9–1 mm long, straight and very thickened throughout its length, fleshy, constricted at the apex; anthers 0.6–1 mm long; apiculus ca. 0.1 mm long, acute to apiculate, connate or slightly separate. ***Pistillate inflorescences*** 3.2–4 cm long (immature), axillary, axes flattened or irregularly flattened, tomentose, with trichomes stellate, brown to ferruginous; peduncle 0.6–1.2 × ca. 3.2 cm long; bracts not seen. ***Pistillate flowers*** not seen. ***Infructescence*** 3.2–5 cm long, with (1–) 2–4 fruits, peduncle 1–3.5 × 0.35–0.53 cm. ***Fruits*** (2.7–) 3.5–4.5 × (1.9–) 2.3–2.9 cm, ellipsoid, shortly stipitate, densely tomentose, the trichomes stellate, ferruginous, persistent, the surface rugose when dry, the line of dehiscence conspicuously carinate, the base obtuse to subtruncate, the apex acute to acuminate, brown (possibly by pubescence), although presumably green when fresh; pericarp (2.7–) 3–4.7 mm thick; pedicel 0.5–0.8 (–1) cm long; seed 2.5–2.8 × 1.5–1.7 cm, the testa dark brown when dry, markedly grooved; aril described as red when fresh, brown to blackish when dry, membranaceous, with a dry texture, thin, laciniate in narrow bands distally.

#### Distinctive characters.

*Virola otobifolia* is recognised by its large leaf blades with well-spaced lateral veins (Fig. 8M) and a whitish abaxial surface that is covered with stellate, sessile trichomes (Fig. 3M); staminate flowers that have a filament column that is straight and very thickened throughout its length, except where it is constricted at the apex and anthers that are almost the same length as the filament column; and its large fruits with thick pericarp that are densely tomentose and with a conspicuously carinate line of dehiscence (Fig. 4C).

#### Etymology.

The specific epithet refers to the similarity of the leaf blades with *Otoba*, another member of Myristicaceae. This epithet was, in part, inspired by the fact that some of the first collections of this new species were initially confused with this genus (as *Dialyanthera* Warb.; e.g. *A. Gentry & S. Mori 14199*, MO).

#### Distribution.

**Figure 27.**
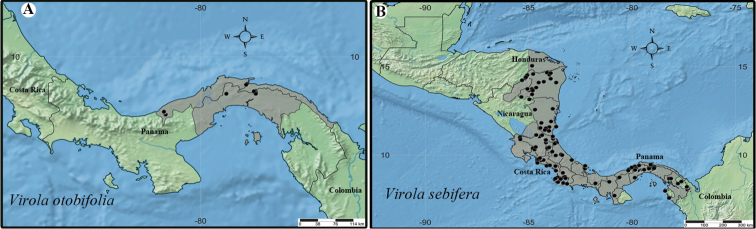
Geographic distribution of *Virola otobifolia* (**A**) and *V. sebifera* (**B**).

*Virola otobifolia* is only known in Panama (Colón, Panamá and San Blas), where it is found on the Caribbean slope (Fig. 27A). It has been recorded between 50–850 m elevation.

#### Preliminary conservation status.

*Virola obtobifolia* is Endangered following IUCN criteria B1a and B2a. Justifying this status, it has both a small EOO (3,269 km^2^) and AOO (36 km^2^) and is known from only five localities.

#### Common names.

Panama: velario, miguelario; cuinur burwi, putmas (Kuna).

#### Phenology.

*Virola otobifolia* has been recorded with flowers and fruits in February to April and one collection with fruits was made in October.

#### Field characters.

Exudate is slow to appear and is watery and red-black. Leaf blades are whitish abaxially. Flowers have yellow perianth. Fruits are green, but appearing brown (possibly due to pubescence).

#### Discussion.

Specimens of *Virola otobifolia* have been confused with and identified as *V. macrocarpa* (e.g. Correa et al. 2004; Condit et al. 2011) (Figs 4D, 7C), probably due to the similar size of the fruits and the leaf morphology (i.e. large discolorous leaf blades, with stellate, sessile trichomes with a reddish centre, and lateral veins that are well-separated). However, it is easily distinguished by the leaf width, pubescence on abaxial surface of the leaves, the number of lateral veins, fruit morphology and habitat; a comparative table of these two species is presented in Table 7.

**Table 7.** Morphological comparison between *Virola otobifolia* and *V. macrocarpa*.

Characters	*V. otobifolia*	*V. macrocarpa*
Leaf blades	(14–) 18.2–42.5 × (4.1–) 7.3–14.2 cm; dense, but inconspicuously pubescent on abaxial side	20–40 × 7–11 cm; inconspicuously puberulent on abaxial side^*^
Lateral veins	10–16 per side, 2–4 veins per 5 cm, 1.7–3 cm apart	14–16 per side^*^, 4–5 veins per 5 cm, 0.8–1.5 cm apart
Petiole	(1–) 1.3–2 × 0.19–0.44 cm	1.5–2.3^*^ × 0.18–0.23 cm
Fruit	(2.7–) 3.5–4.5 × (1.9–) 2.3–2.9 cm; densely tomentose, with persistent trichomes; the surface rugose when dry, the line of dehiscence conspicuously carinate.	2.7–3.3 × 2–2.3 cm^*^; tomentellous, with cauducous trichomes that are dust-like when they fall; the surface smooth when dry, the line of dehiscence slightly carinate.
Pericarp	(2.7–) 3–4.7 mm thick	1.8–3 mm thick
Habitat	Lowland or premontane rain forest, Central Panama (Panamá, San Blas) at 50–850 m elevation	Montane forests at high elevations Andes of Colombia (Boyacá) at 1100 m elevation^*^

^*^ From Smith and Wodehouse 1938 (except the pericarp).

Two similar species from Mesoamerica that resemble *Virola otobifolia* are: *V. allenii* (Figs 6, 7A), which ocurrs in the lowland wet forest on the Pacific slope of Costa Rica and *V. amistadensis* (Fig. 7B) from montane forests of Costa Rica and Panama on the Caribbean slope. Morphological comparison between these species is presented in Table 3.

#### Notes.

The holotype, deposited at Missouri Botanical Garden (MO), represents a single collection mounted on two sheets that are clearly labelled (“Sheet 1 of 2,” “Sheet 2 of 2) as being parts of the same specimen (ICN Art. 8.3) (Turland et al. 2018).

#### Specimens examined.

**Panama. Colón**: Teck Cominco Petaquilla, 200 m elev., 21 Feb 2008 (fl bud), *G. McPherson 20135* (MO!); [Donoso] Westernmost part of province, site of proposed copper mine (INMET), 150 m elev., 12 Apr 2009 (fr), *G. McPherson 20905* (MO!). **Panamá**: [Chepo] El Llano Cartí road, near El Llano, [460 m elev.], 27 Mar 1976 (fr), *T. B. Croat 33725* (MO!); Cerro Jefe, Parque Nacional Chagres, [985 m elev.], 17 Jan 2002 (fr), *N. Flores & R. Aizprúa B3156* (MO!); [Chepo] El Llano Cartí road 18 km from Pan-Americana Highway, 330–370 m elev., 14 Feb 1975 (fr), *A. Gentry & S. Mori 14199* (MO!, SCZ!*); El Llano-Carti road, 12.7 km N from Pan American Highway, 350 m elev., 15 Feb 1975 (fl bud), *A. Gentry & S. Mori 14213* (MO!); below Cerro Jefe, along road to Río Pacora, 850 m elev., 09 Jan 1986 (fr), *G. McPherson 7946* (INPA!*, MO!, PMA!*). **San Blas**: El Llano-Cartí road, 24.5–25 km from PanAmerican highway, [250–350 m elev.], 12 Apr 1975 (♂ fl), *S. Mori & J. Kallunki 5542* (MO!); El Llano-Carti road, 350 m elev., 01 Oct 1984 (fr), *G. de Nevers & H. Herrera 3981* (MO!, MEXU!*, PMA!*); Cangandí, 30 m elev., 10 Feb 1986 (fr), *G. de Nevers & H. Herrera 7056* (MO!, INPA!*, PMA!*); ibid, 10 Feb 1986 (fl bud), *G. de Nevers & H. Herrera 7068* (MO!, INPA!*); Cangandí, 30 m elev., 27 Mar 1986 (fr), *G. de Nevers et al. 7430* (MO-2 sheets!, PMA!*); ibid, 27 Mar 1986 (fl bud), *G. de Nevers et al. 7448* (MO!); ibid, 27 Mar 1986 (♂ fl), *G. de Nevers et al. 7530* (MEXU!*, MO!, PMA!*); ibid, 27 Mar 1986 (fl bud), *G. de Nevers et al. 7605, 7607* (MEXU!*, MO!, PMA!*).

### 14. Virola sebifera

Taxon classificationPlantaeMagnolialesMyristicaceae

Aubl.

6543C7FD-082F-5141-8D92-4FD226374777

Fig. 28

Virola sebifera Aubl. Hist. Pl. Guiane. 2: 904. 1775. Type. French Guiana. Cayenne, 1775, [J.] *Aublet s.n*., (holotype: BM!*; isotypes: NY photograph of BM holotype). Fide Jaramillo et al. 2004.
Myristica panamensis Hemsl. Biol. Cent.-Amer., Bot. 3: 67. 1882. Virola panamensis (Hemsl.) Warb., Nova Acta Acad. Caes. Leop.-Carol. German. Nat. Cur. 68: 185. 1897. Syntypes: Panama. shady forest near Cruces, n.d. (fls.), *Seemann 545* (BM!*, K!*); [Canal Area], Lion Hill station, [28 Mar 1862, leaves, excl. fr], *S. Hayes 618* (K!*).
Virola warburgii Pittier. Contr. U.S. Natl. Herb. 18: 143. 1916. Type. Panama. Panamá, collected in forests along the Chagres River above Alhajuela, 12 May 1911 [fls], *H. Pittier 3505* (holotype: US; isotype: BM).

#### Distinctive characters.

*Virola sebifera* is recognised by its dense pubescence of pediculate and ferruginous trichomes (Fig. 3N) that cover almost all parts of the plant; leaf blades with few lateral veins (9–17 per side), well-spaced between them [3–4 (–5) veins per 5 cm, 1.3–2.7 cm apart] (Fig. 8N); the long staminate inflorescences that are very branched; the staminate flowers with the filament column short (0.1–0.4 mm long) and the anthers more than twice the length of the column of the filaments (0.6–0.8 mm long) and apiculate at the apex; its small fruits [1.1–1.7 (–2.3) × 0.8–1.3 (–1.6) cm] that are globose to subglobose (Fig. 4K), covered by a dense layer of trichomes that fall easily and a thin pericarp [0.3–0.6 (–0.9) mm]; and the thin aril that is laciniate in narrow bands. There are also usually many fruits per infructescence.

#### Distribution.

*Virola sebifera* is the most collected and widespread species in Mesoamerica. It is known from Honduras (Gracias a Dios), Nicaragua (Atlántico Norte, Atlántico Sur, Jinotega, Matagalpa and Río San Juan), Costa Rica (Alajuela, Cartago, Guanacaste, Heredia, Limón, Puntarenas and San José) and Panamá (Bocas del Toro, Chiriquí, Coclé, Colón, Darién, Herrera, Panamá, San Blas and Veraguas) (Fig. 27B). It has been recorded between 0–1000 m elevation.

#### Common names.

Honduras: walus, banak. Nicaragua: banak, cebo, cebo macho, cebo sirsio, sangre drago. Costa Rica: coton, fruta dorada, miguelario. Panama: bogamani, copidijo, fruta dorada, gorgoran, malagueta de montaña, mancha, sangre, tabegua, velario colorado.

**Figure 28.**
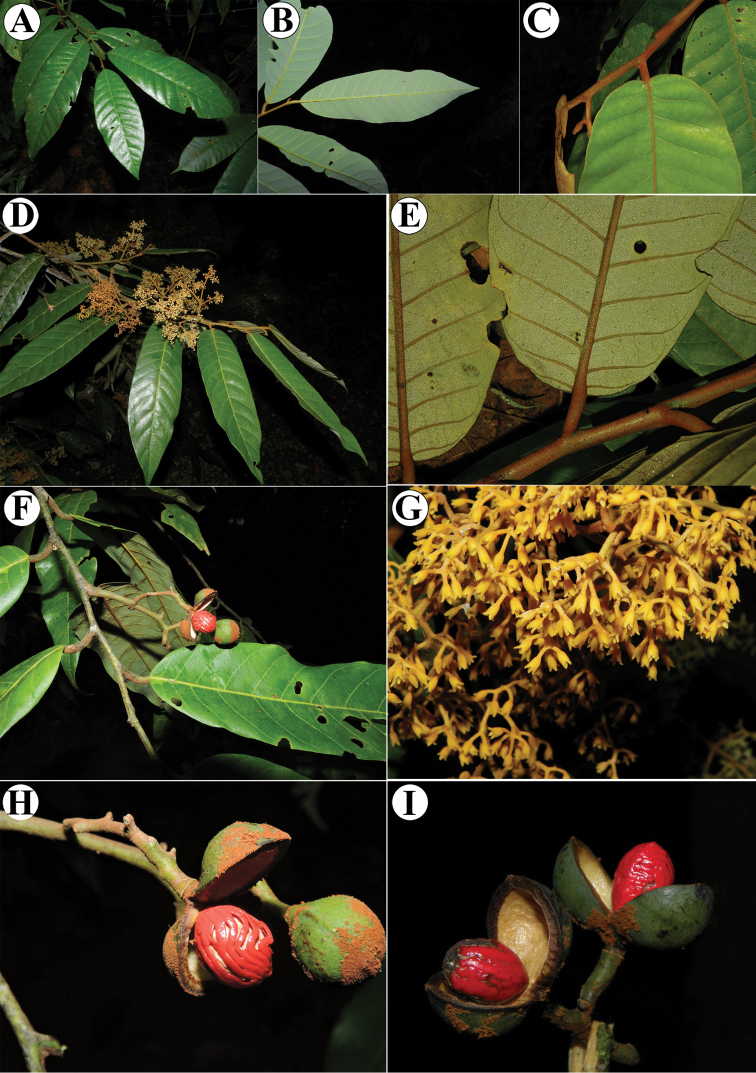
*Virola sebifera***A** leaf blade on adaxial surface **B** leaf blade on abaxial surface **C, E** leaf base on both sides **D** flowering branch **G** staminate flowers **H** infructescence **I** fruits and aril. Photos by Reinaldo Aguilar.

#### Phenology.

Herbarium specimens of flowering and fruiting individuals have been collected throughout the year. In the Osa Peninsula and Golfo Dulce, Costa Rica, flowering occurs in December to May.

#### Field characters.

Plants are shrubs or trees between 8–25 (–40) m tall and 7–40 cm DBH. Trunks are straight, sometimes with small buttresses and with long and horizontal branches. Bark is described as dark, minutely fissured vertically or flaking off in small pieces and aromatic. Exudate from different parts of the plant is red or reddish. The leaf blades are bright green above and whitish to greyish below in living material. Flowers have brown, cream, orange, yellow-brown or yellowish perianth and are sometimes fragrant, with a lemon or sweet aroma. The mature fruit is green and covered by a dense layer of trichomes, with a white seed with a red aril.

#### Notes.

The leaves of this species are very variable in shape and size. Sometimes, a single individual may have leaves of different sizes. Individuals with small leaf blades from Panama (e.g. *G. McPherson 15341*, MO; *G. McPherson 20671*, MO), that are sparsely pubescent and with sessile trichomes on the abaxial surface of the leaf, may be difficult to distinguish from *V. elongata*. However, specimens of *V. sebifera* tend to have thicker twigs, petioles and floriferous and fruiting peduncles.

#### Selected specimens seen.

**Honduras. Gracias a Dios**: Monte Alto de Anastasio, 90 m elev., 17 Mar 1994 (♂ fl), *P. R. House 2019* (MO!); al sur de Krausirpe, 25 Mar 1995 (♀ fl), *P. R. House 2351* (MO!); 30 minutos al sur de Krausirpe, 11 May 1994 (fr), *P. R. House 2814* (MO!). **Nicaragua. Atlántico Norte**: 13 km above Kururia on road to San Jerónimo, 200 m elev., 02 Mar 1979 (♂ fl), *J. J. Pipoly 3844* (MO!); Siuna, La Pimienta, [150 m elev.], 01 Jun 1984 (fr), *F. Ortiz 1971* (MO!). **Atlántico Sur**: El Zapote, 40 km NE de Nueva Guinea, 130–150 m elev., 27 Feb 1984 (♂ fl), *J. C. Sandino 4767* (MO!); Boca de Sábalo, 70 m elev., 24 Mar 1985 (♀ fl), *P. P. Moreno 25639* (MO!). **Jinotega**: Wiwili, zona de amortiguamieno Bosawas, 170 m elev., 18 Jan 2006 (♀ fl), *I. Coronado et al. 2949* (MO!); Wiwili, Reserva de Bosawas, 220 m elev., 09 Feb 2006 (♂ fl), *I. Coronado et al. 3388* (MO!); Wiwili, Reserva de Bosawas, 170 m elev., 09 Jun 2007 (fr), *I. Coronado et al. 3969* (MO!). **Río San Juan**: 2 km al NW del Río Santa Cruz, 70 m elev., 24 Feb 1984 (♀ fl), *P. P. Moreno 23328* (MO!); El Castillo, Finca de Madrid, 100 m elev., 18 Jan 2005 (♂ fl), *R. Loredo 2348* (MO!); entre el pueblo de San Juan del Norte Nuevo y la casa de Ramón Castillo, 0–100 m elev., 07 Jul 1994 (fr), *R. Rueda et al. 1851* (MO!). **Costa Rica. Alajuela**: Reserva Biológica de Monteverde, río Caño Negro, 800 m elev., 19 Oct 1989 (fr), *E. Bello 1402* (MO!); Upala, Estación San Ramón, 550 m elev., 16 Mar 1993 (♂ fl), *R. Espinoza 803* (MO!); San Carlos, Fortuna, 255–400 m elev., 27 May 2004 (fr), *A. Rodríguez 9046* (MO!). **Cartago**: Turrialba, El Zapote Nature Reserve, 700 m elev., 01 Mar 1987 (imm fr), *W. Burger & J. Gómez-Laurito 12030* (CR!, MEXU!*). **Guanacaste**: Finca La Pasmompa, 400 m elev., 10 Dec 1990 (fl bud), *C. Moraga 227* (MO!). **Heredia**: Tirimbina, Istarú farm, 220 m elev., 28 Jan 1972 (♂ fl), *R. W. Lent 2329* (MO); Finca La Selva, [90 m elev.], 09 Jul 1984 (fr), *D. Smith 1088* (MO!). **Limón**: Parque Nacional Barbilla, 300–400 m elev., 22 May 2000 (fl bud), *E. Mora 1155* (MO); Cerro Coronel, 20–170 m elev., 16–23 Jan 1986 (♂ fl), *W. D. Stevens 23823* (MO!). **Puntarenas**: Golfito, Mata Palo, 160–200 m elev., 09 Sep 1991 (fr), *R. Aguilar 363* (MO!); Golfito, 1 km antes de llegar a la Palma, 8 m elev., 16 Jan 1993 (♂ fl), *R. Aguilar 1586* (MO!); Punta Burica, 0–100 m elev., 22 Aug 1988 (fr), *M. M. Chavarría et al. 292* (MO-2 sheets!). **San José**: Puriscal, San Martín, 800 m elev., 20 Nov 1993 (fr), *J. F. Morales 2022* (MO!); vicinity of El General, 915 m elev., n.d. May 1937 (♂ fl), *A. F. Skutch 3118* (MO!); Turrubares, San Rafael, 600–700 m elev., 08 Dec 2004 (fr), *A. Soto & D. Santamaría 409* (MO!). **Panama. Bocas del Toro**: Isla Bastimentos, Bocatorito, 30 m elev., 15 Feb 1989 (♀ fl), *P. M. Peterson & C. R. Annble 6872* (MO!). **Chiriquí**: 17 km NE of San Felix, 1000 m elev., 18–19 Mar 1974 (fr), *M. Nee 10664* (MO!). **Coclé**: Caribbean side of divide at El Copé, 200–400 m elev., 04 Feb 1983 (fr), *C. Hamilton & G. Davidse 2756* (MO!). **Colón**: Area near Guasimo, no elev., 22 Apr 1970 (♂ fl), *T. B. Croat 9950* (MO!); Santa Rita ridge, 500 m elev., 13 Jan 1987 (fr), *G. McPherson 10265* (MO!). **Darién**: Vicinity of Cana, 1750 ft [530 m] elev., 24 Jun 1959 (♂ fl), *W. L. Stern 512* (MO!); serranía de Sapo, 300–800 m elev., 26 Nov 1990 (fr), *H. Herrera & J. Polanco 793* (MO!). **Herrera**: Road between Las Minas and El Toro, 700–800 m elev., 23 Jan 1987 (fr), *G. McPherson 10273* (MO!). **San Blas**: El Llano Cartí road, 350 m elev., 06 May 1985 (fr), *G. de Nevers 5609* (MO!). **Panamá**: Barro Colorado Island, [10–100 m elev.], 13 Dec 1967 (fr), *T. B. Croat 4275* (MO!); along Pipeline Road, 125 m elev., 10 Mar 1974 (♂ fl), *M. Nee 10445* (MO!); Llano Cartí road, 350 m elev., 26 Jan 1986 (fr), *G. McPherson & M. Merello 8148* (MO!). **Veraguas**: Cerro Hoya, subiendo por Cobachón, [750 m elev.], 21 Apr 1997 (fl), *J. Deago 499* (PMA!*).

## Conclusions

In this synopsis of Mesoamerican *Virola*, 14 species are recognised. Twelve of these are restricted to Mesoamerica, while the other two species extend into South America. Six of the treated species are new; these are all from Costa Rica and Panama, countries with the highest species diversity of *Virola*, with 10 and 11 species, respectively. We clarified the application of some names, the resurrection of others and the distribution of some species previously considered to occur in Mesoamerica (e.g. South American *V. macrocarpa*) or South America (e.g. the Mesoamerican *V. multiflora*). This has resulted in a much modified taxonomy for *Virola*, summarised here: Mesoamerican collections previously identified as *V. calophylla* and/or *V. macrocarpa* are here interpreted as three new species: *V. alleni*, *V. amistadensis* and *V. otobifolia*, while Mesoamerican specimens previously determined as *V. surinamensis* are treated as *V. nobilis*. As a result of this, *V. calophylla* and *V. macrocarpa* are now restricted to South America and *V. surinamensis* to South America and the Antilles. Additionally, two new species are identified within what was previously *V. guatemalensis*: montane collections from the southern range now belong to *V. montana* and we have resurrected *V. laevigata*, previously considered a synonym. Under our concept, *Virola guatemalensis* has a restricted range, found only in northern Mesoamerica. Finally, the majority of *V. koschnyi* specimens from the Pacific coast of Costa Rica and Panama correspond to *V. chrysocarpa*, while most of Panama’s collections, identified as *V. multiflora*, are now considered *V. fosteri*.

These contributions came to light via detailed comparisons across the complete range of these species thanks to hundreds of collections made by numerous botanists and herbaria who care, maintain and provide access to the specimens, including making them available online.

## Supplementary Material

XML Treatment for Virola allenii

XML Treatment for Virola amistadensis

XML Treatment for Virola chrysocarpa

XML Treatment for Virola elongata

XML Treatment for Virola fosteri

XML Treatment for Virola guatemalensis

XML Treatment for Virola koschnyi

XML Treatment for Virola laevigata

XML Treatment for Virola megacarpa

XML Treatment for Virola montana

XML Treatment for Virola multiflora

XML Treatment for Virola nobilis

XML Treatment for Virola otobifolia

XML Treatment for Virola sebifera

## Supplementary materials

Supplementary material 1 List of exsiccatae Data type: List of specimens examined. https://binary.pensoft.net/file/349111This dataset is made available under the Open Database License (http://opendatacommons.org/licenses/odbl/1.0/). The Open Database License (ODbL) is a license agreement intended to allow users to freely share, modify, and use this Dataset while maintaining this same freedom for others, provided that the original source and author(s) are credited.Santamaría-Aguilar Daniel, Aguilar Reinaldo, Lagomarsino Laura Penolope

## References

[B1] Acevedo-Rodríguez P Strong MT (2012) Catalogue of seed plants of the West Indies. Smithsonian Contributions to Botany 98: 1–1192. 10.5479/si.0081024X.98.1

[B2] Achá S Liesner RL (2014) Myristicaceae. In: Jørgensen PM Nee MH Beck SG (Eds) Catálogo de las plantas vasculares de Bolivia.Monographs in Systematic Botany from the Missouri Botanical Garden 127(1), 868–870.

[B3] Aguilar R Cornejo X Santamaría-Aguilar D Tulig M Bainbridge C Mori SA (2017 onward) Vascular Plants of the Osa Peninsula, Costa Rica. http://sweetgum.nybg.org/osa/

[B4] Allen PH (1956) The Rain Forests of Golfo Dulce. University of Florida Press Gainesville, 1–417.

[B5] APG IV (2016) An update of the Angiosperm Phylogeny Group classification for the orders and families of flowering plants: APG IV. Botanical Journal of the Linnean Society 181(1): 1–20. 10.1111/boj.12385

[B6] Aublet JBCF (1775) Histoire des plantes de la Guiane Françoise: Rangées suivant la méthode sexuelle, avec plusieurs mémoires sur différens objects intéressans, relatifs à la culture & au commerce de la Guiane Françoise, & une notice des plantes de l'Isle-de-France. Tome Second. Pierre-François DIDOT, London & Paris 2: 622–976. 10.5962/bhl.title.48831

[B7] Aymard GA Berry PE Rodrigues WA (2007) Myristicaceae. In: Funk VA Hollowell TH Berry PE Kelloff CL Alexander S (Eds) Checklist of the plants of the Guiana Shield (Venezuela: Amazonas, Bolivar, Delta Amacuro; Guyana, Suriname, French Guiana).Contributions from the United States National Herbarium 55: 428–430.

[B8] Balick MJ Nee MH Atha DE (2000) Checklist of the vascular plants of Belize. Memoirs of the New York Botanical Garden, 246 pp.

[B9] Bennett BC Alarcón R (1994) *Osteophloeum platyspermum* and *Virola duckei* (Myristicaceae): Newly reported as hallucinogens from Amazonian Ecuador. Economic Botany 48(2): 152–158. 10.1007/BF02908205

[B10] Bentham G (1853) Notes on the American species of *Myristica*. Hooker’s Journal of Botany and Kew Garden Miscellany 5: 1–9. https://www.biodiversitylibrary.org/item/6323#page/7/mode/1up

[B11] Cardona NF David HH Hoyos SEG (2010) Flora de la Miel, Central Hidroeléctrica Miel I, Oriente de Caldas, Guía ilustrada. ISAGEN – Universidad de Antioquia, Herbario Universidad de Antioquia (HUA), Medellín, 228 pp.

[B12] Cardoso D Särkinen T Alexander S Amorim AM Bittrich V Celis M Daly DC Fiaschi P Funk VA Giacomin LL Goldenberg R Heiden G Iganci J Kelloff CL Knapp S Cavalcante de Lima H Machado AFP dos Santos RM Mello-Silva R Michelangeli FA Mitchell J Moonlight P de Moraes PLR Mori SA Nunes TS Pennington TD Pirani JR Prance GT de Queiroz LP Rapini A Riina R Rincon CAV Roque N Shimizu G Sobral M Stehmann JR Stevens WD Taylor CM Trovó M van den Berg C van der Werff H Viana PL Zartman CE Forzza RC (2017) Amazon plant diversity revealed by a taxonomically verified species list. Proceedings of the National Academy of Sciences of the United States of America 114(40): 10695–10700. 10.1073/pnas.1706756114 28923966 PMC5635885

[B13] Cogollo Á (2011) Myristicaceae. In: Idárraga-Piedrahita A Ortiz RDC Callejas Posada R Merello M (Eds) Flora de Antioquia: Catálogo de las Plantas Vasculares, Listado de las plantas vasculares del departamento de Antioquia.Universidad de Antioquia, Medellín 2: 641–644.

[B14] Cogollo Á Velásquez-Rúa C García N (2007) Las miristicáceas. In: García N (Ed.) Libro Rojo de Plantas de Colombia. Las magnoliáceas, las miristicáceas y las podocarpáceas. Serie Libros Rojos de Especies Amenazadas de Colombia. Bogotá, Colombia.Instituto Alexander von Humboldt – CORANTIOQUIA – Jardín Botánico Joaquín Antonio Uribe de Medellín - Instituto de Ciencias Naturales de la Universidad Nacional de Colombia – Ministerio de Ambiente, Vivienda y Desarrollo Territorial 5: 155–192.

[B15] Condit R Pérez R Daguerre N (2011) Trees of Panama and Costa Rica. Princeton University Press, New Jersey, 494 pp 10.1515/9781400836178

[B16] Cornejo X Mori SA Aguilar R Stevens H Douwes F (2012) Phytogeography of the trees of the Osa Peninsula, Costa Rica. Brittonia 64(1): 76–101. 10.1007/s12228-011-9194-0

[B17] Correa MD Galdames C Stapf M (2004) Catálogo de las Plantas Vasculares de Panamá. Smithsonian Tropical Research Institute, Panamá, 599 pp.

[B18] Croat TB (1978) Flora of Barro Colorado Island. Stanford University Press, Stanford, California, 960 pp.

[B19] Dauby G Stévart T Droissart V Cosiaux A Deblauwe V Simo-Droissart M Sosef MSM Lowry PP II Schatz GE Gereau RE Couvreur TLP (2017) ConR: An R package to assist large-scale multispecies preliminary conservation assessments using distribution data. Ecology and Evolution 7(24): 11292–11303. 10.1002/ece3.3704 29299301 PMC5743656

[B20] de Candolle A (1856) Ordo CLXIII. Myristicaceae. In: de Candolle A (Ed.) Prodromus Systematis Naturalis Regni Vegetabilis 14: 189–208.

[B21] de Wilde WJJO (1991) The genera of Myristicaceae as distinguished by their inflorescences, and the description of a new genus, *Bicuiba*. Beiträge zur Biologie der Pflanzen 66: 95–125.

[B22] Ducke A (1936) Notes on the Myristicaceae of Amazonian Brazil with descriptions of new species. Journal of the Washington Academy of Sciences 26(5): 213–222. https://www.jstor.org/stable/24530511

[B23] Ducke A (1945) New forest trees and climbers of the Brazilian Amazon. Boletim Técnico do Instituto Agronômico de Norte 4: 8–12.

[B24] Duke JA (1962) Myristicaceae. Flora of Panama, Part IV. Fascicle 5. Annals of the Missouri Botanical Garden 49(3–4): 214–225. 10.2307/2394708

[B25] Duke JA (1975) Ethnobotanical observations on the Cuna Indians. Economic Botany 29(3): 278–293. 10.1007/BF02873178

[B26] Flores EM (2010) *Virola koschnyi* Warb. In: Vozzo JA (Ed.) Manual de Semillas de Árboles Tropicales. Departamento de Agricultura de los Estados Unidos, Servicio Forestal (USDA), Washington, 887 pp.

[B27] Flores-Vindas E Obando-Vargas G (2014) Árboles del Trópico Húmedo, Importancia socioeconómica. Second edition. Editorial Tecnológico de Costa Rica, 11–996.

[B28] Frankie FW Baker HG Opler PA (1974) Comparative Phenological Studies of Trees in Tropical Wet and Dry Forests in the Lowlands of Costa Rica. Journal of Ecology 62(3): 881–919. 10.2307/2258961

[B29] Garwood NC (2009) Seedlings of Barro Colorado Island and the Neotropics. Cornell University Press, Ithaca, 645 pp.

[B30] Gentry AH (1975) Additional Panamanian Myristicaceae. Annals of the Missouri Botanical Garden 62(2): 474–479. 10.2307/2395207

[B31] Gentry AH (1993) A field guide to the families and genera of woody plants of northwest South America (Colombia, Ecuador, Peru), with supplementary notes on herbaceous taxa. Conservation International, Washington, D.C., 895 pp.

[B32] Gentry AH (2001) Myristicaceae. In: Stevens WD Ulloa Ulloa C Pool A Montiel OM (Eds) Flora de Nicaragua.Monographs in Systematic Botany from the Missouri Botanical Garden 85(1), 1542–1545.

[B33] Gómez-Laurito J Ortiz R (2004) Lista con Anotaciones de las Angiospermas de la Reserva Biológica Alberto Brenes (Microcuencas de los ríos San Lorenzo y San Lorencito), Costa Rica. Lankesteriana 4(2): 113–142. 10.15517/lank.v4i2.21614

[B34] Gradstein SR (2016) *Virola* In: Bernal R, Gradstein SR, Celis M (Eds) Catálogo de plantas y líquenes de Colombia. Instituto de Ciencias Naturales, Universidad Nacional de Colombia, Bogotá. http://catalogoplantasdecolombia.unal.edu.co

[B35] Grayum MH Hammel BE Troyo S Zamora N (2004) Historia/History. In: Hammel BE Grayum MH Herrera C Zamora N (Eds) Manual de Plantas de Costa Rica. Vol. I. Introducción.Monographs in Systematic Botany from the Missouri Botanical Garden 97: 1–50.

[B36] Haber W (2014) Plantas Vasculares de Monteverde. Apéndice 1. In: Nadkarni NM Wheelwright NT (Eds) Monteverde: ecología y conservación de un bosque nuboso tropical (versión actualizada y ampliada en español).Oxford University Press, 744–805. http://digitalcommons.bowdoin.edu/scholars-bookshelf/3/

[B37] Hallé F Oldeman RAA Tomlinson PB (1978) Tropical Trees and Forests, An Architectural Analysis. Springer-Verlag Berlin Heidelberg, New York, 441 pp 10.1007/978-3-642-81190-6_1

[B38] Hemsley WB (1882) Botany. In: Godman FD Salvin O (Eds) Biologia Centrali-Americana; or Contributions to the Knowledge of the Fauna and Flora of Mexico and Central America. R. H. Poret and Dulau & Co., London 3: 1–711.

[B39] Howe HF (1981) Dispersal of a Neotropical Nutmeg (*Virola sebifera*) by birds. The Auk: Ornithological Advances 98(1): 88–98. https://doi-org.eres.qnl.qa/10.1093/auk/98.1.88

[B40] Howe HF (1982) Fruit production and animal activity in two tropical trees. In: Leigh EG Jr Stanley Rand A Windsor DM (Eds) The Ecology of a tropical forest: seasonal rhythms and long-term changes.Smithsonian Institution Press, Washington, 189–200.

[B41] Howe HF (1993) Aspects of variation in a neotropical seed dispersal system. In: Fleming TH Estrada A (Eds) Frugivory and seed dispersal: ecological and evolutionary aspects.Vegetation 107/108, 149–162. 10.1007/978-94-011-1749-4_10

[B42] Howe HF Vande Kerckhove GA (1980) Nutmeg dispersal by tropical birds. Science 210(4472): 925–927. 10.1126/science.210.4472.925 17800844

[B43] Janovec PJ (2000) A systematic study of *Compsoneura* (A. DC.). Warb., a Neotropical Member of the Nutmeg family (Myristicaceae). PhD Thesis, Texas A & M University.

[B44] Jaramillo TS Muriel P Rodrigues WA Balslev H (2000) Myristicaceae novelties from Ecuador. Nordic Journal of Botany 20(4): 443–447. 10.1111/j.1756-1051.2000.tb01586.x

[B45] Jaramillo TS Muriel P Balslev H (2004) Myristicaceae. In: Harling GW Andersson L (Eds) Flora of Ecuador.University of Göteborg, Göteborg 72: 1–101.

[B46] Jiménez Q (2007) Myristicaceae. In: Hammel BE Grayum MH Herrera C Zamora N (Eds) Manual de Plantas de Costa Rica. Vol. VI.Monographs in Systematic Botany from the Missouri Botanical Garden 111: 684–691.

[B47] Jiménez Madrigal Q Grayum MH (2002) Vegetación del Parque Nacional Carara, Costa Rica. Brenesia 57–58: 25–66.

[B48] Jørgensen PM León-Yánez S (1999) Catalogue of the Vascular Plants of Ecuador. Monographs in Systematic Botany from the Missouri Botanical Garden 75: 1–1181.

[B49] Knapp S (2013) A revision of the Dulcamaroid Clade of *Solanum* L. (Solanaceae). PhytoKeys 22(0): 1–428. 10.3897/phytokeys.22.4041 PMC3689140 23794937

[B50] Kühn U Kubitzki K (1993) Myristicaceae. In: Kubitzki K Rohwer JG Bittrich V (Eds) The Families and Genera of Vascular Plants. Flowering Plants. Dicotyledons. Magnoliid, Hamamelid, and Caryophyllid families.Springer Verlag, Berlin 2: 457–467. 10.1007/978-3-662-02899-5_53

[B51] Lambright DD (1981) The comparative anatomy and morphology of *Virola* (Myristicaceae). PhD Thesis, Miami University, Oxford, Ohio.

[B52] Ley López JM Chacón Madrigal E (2017) Las Plántulas de Árboles y Palmas de la Península de Osa. San José, Costa Rica, 3–183.

[B53] Little EL (1970) New Tree Species from Esmeraldas, Ecuador. Phytologia 19(4): 251–269. 10.5962/bhl.part.14772

[B54] Lobo J Aguilar R Chacón E Fuchs E (2008) Phenology of tree species of the Osa Peninsula and Golfo Dulce region, Costa Rica. In: Weissenhofer A Huber W Mayer V Pamper S Weber A Aubrecht G (Eds) Natural and Cultural History of the Golfo Dulce Region, Costa Rica.Stapfia 88: 547–555.

[B55] Mari Mut JA (2018) Notes on the etymology of Aublet’s generic names, 1–17. https://archive.org/details/aubletgen [Last updated: April 30, 2018]

[B56] Mitchell JD (2002) Myristicaceae (Nutmeg Family). In: Mori SA Cremers G Gracie CA de Granville JJ Heald SV Hoff M Mitchell JD (Eds) Guide to the vascular plants of Central French Guiana.Memoirs of The New York Botanical Garden 76(2), 525–532.

[B57] Monro AK Santamaría-Aguilar D González F Chacón O Solano D Rodríguez A Zamora N Fedele E Correa MDA (2017) A first checklist to the vascular plants of La Amistad International Park (PILA), Costa Rica-Panama. Phytotaxa 322(1): 1–283. 10.11646/phytotaxa.322.1.1

[B58] Moreira JI Riba-Fernández P Lobo J (2017) Toucans (*Ramphastos ambiguus*) facilitate resilience against seed dispersal limitation to a large‐seeded tree (*Virola surinamensis*) in a human‐modified landscape. Biotropica 49(4): 502–510. 10.1111/btp.12427

[B59] Moya Roque R Rodríguez González A Olivares Gutiérrez C (2014) Árboles maderables de la Península de Osa, madera y corteza. Editorial Tecnológico de Costa Rica, Cartago, 344 pp.

[B60] Nelson CH (2008) Catálogo de las Plantas Vasculares de Honduras. Secretaría de Recursos Naturales y Ambiente, Tegucigalpa, 1576 pp.

[B61] Pennington TD Sarukhánn J (2016) Árboles Tropicales de México, Manual para la Identificación de Campo de las Principales Especies. Universidad Nacional Autónoma de México y Fondo de Cultura Económica. Ciudad de México 3: 9–511.

[B62] Pérez R Condit R (2018) Tree Atlas of Panama. http://ctfs.si.edu/webatlas/maintreeatlas.php

[B63] Pesce C (2009) Oleaginosas da Amazônia. Second edition. Belém: Museu Paraense Emílio Goeldi; Brasília: Ministério do Desenvolvimento Agrário 23–334.

[B64] Pitman NCA Mogollón H Dávila N Ríos M García‐Villacorta R Guevara J Baker TR Monteagudo A Phillips OL Vásquez‐Martínez R Ahuite M Aulestia M Cardenas D Cerón CE Loizeau PE Neill DA Núñez P Palacios WA Spichiger R Valderrama E (2008) Tree community change across 700 km of Lowland Amazonian Forest from the Andean Foothills to Brazil. Biotropica 40(5): 525–535. 10.1111/j.1744-7429.2008.00424.x

[B65] Pittier H (1916) New or noteworthy plants from Colombia and Central America (V). Contributions from the United States National Herbarium 18(4): 143–171.

[B66] Pittier H (1922) New or noteworthy plants from Colombia and Central America (VIII). Contributions from the United States National Herbarium 20(12): 453–487.

[B67] Pittier H (1978) Plantas usuales de Costa Rica. Editorial Costa Rica, 1–329.

[B68] Plotkin MJ Schultes RE (1990) *Virola*: A Promising Genus for Enthnopharmacological Investigation. Journal of Psychoactive Drugs 22(3): 357–361. 10.1080/02791072.1990.10472561 2286871

[B69] Poinar Jr G Steeves R (2013) *Virola dominicana* sp. nov. (Myristicaceae) from Dominican amber. Botany 91(8): 530–534. 10.1139/cjb-2013-0019

[B70] Prance GT (1970) Notes on the use of plant Hallucinogens in Amazonian Brazil. Economic Botany 24(1): 62–68. 10.1007/BF02860638

[B71] Quesada Quesada FJ Jiménez Madrigal Q Zamora Villalobos N Aguilar Fernández F González Ramírez J (1997) Árboles de la Península de Osa. INBio, Santo Domingo de Heredia, 1–411.

[B72] Riba Hernández JP (2017) Conservación, estructura poblacional y ecología reproductiva de la fruta dorada (*Virola surinamensis*: Myristicaceae) un árbol dioico maderable en la Península de Osa, Costa Rica. PhD Thesis, Universidad de Costa Rica, Costa Rica.

[B73] Rodrigues WA (1972) A ucuuba de várzea e suas aplicações. Acta Amazonica 2(2): 29–47. 10.1590/1809-43921972022029

[B74] Rodrigues WA (1977) Novas espécies de *Virola* Aubl. (Myristicaceae) da Amazônia. Acta Amazonica 7(4): 459–471. 10.1590/1809-43921977074459

[B75] Rodrigues WA (1980) Revisão taxonômica das espécies de *Virola* Aublet (Myristicaceae) do Brasil. Acta Amazonica 10(1): 1–127. 10.1590/S0044-59672006000100001

[B76] Rodrigues WA (1989) A New Venezuelan *Virola* (Myristicaceae). Annals of the Missouri Botanical Garden 76(4): 1163–1164. 10.2307/2399703

[B77] Rodrigues WA (2008) Myristicaceae. In: Hokche O Berry PE Huber O (Eds) Nuevo Catálogo de la Flora Vascular de Venezuela.Fundación Instituto Botánico de Venezuela, Caracas, 512–515.

[B78] Rodrigues WA (2010) Flora da Bahia: Myristicaceae. Sitientibus. Série Ciências Biológicas 10(1): 138–146.

[B79] Rodrigues W (2015) Myristicaceae in Lista de Espécies da Flora do Brasil. Jardim Botânico do Rio de Janeiro. http://floradobrasil.jbrj.gov.br/jabot/floradobrasil/FB10197

[B80] Rodrigues WA Aymard CGA Berry PE (2001) Myristicaceae. In: Steyermark JA Berry PE Holst BK (Eds) Flora of the Venezuelan Guayana.Missouri Botanical Garden Press 6: 734–747.

[B81] Sabatier D (1997) Description et biologie d'une nouvelle espèce de *Virola* (Myristicaceae) de Guyane. Adansonia sér. 3 19(2): 273–278.

[B82] Sánchez Hernández S Mendoza Briseño MA García Hernández RV (2017) Diversificación de la sombra tradicional de cafetales en Veracruz mediante especies maderables. Revista Mexicana de Ciencias Forestales 8(40): 7–18. 10.29298/rmcf.v8i40.32

[B83] Schultes RE (1954) A new narcotic snuff from the Northwest Amazon. Botanical Museum Leaflets 16(9): 241–260.

[B84] Schultes RE (1969) De plantas toxicariis e mundo novo tropicale commentationes V: *Virola* as an orally administered hallucinogen. Botanical Museum Leaflets 22(6): 229–240.

[B85] Schultes RE (1979) Evolution of the identification of the myristicaceous hallucinogens of South America. Journal of Ethnopharmacology 1(3): 211–239. 10.1016/S0378-8741(79)80013-6 396417

[B86] Schultes RE Holmstedt B (1968) De plantis toxicariis e Mundo Novo tropicale commentationes II. The vegetal ingredients of the myristicaceous snuffs of the northwest Amazon. Rhodora 70: 113–160.

[B87] Shorthouse DP (2010) SimpleMappr, an online tool to produce publication-quality point maps. http://www.simplemappr.net [accessed several times 2018]

[B88] Smith AC (1953) Studies of South America plants, XIII. Journal of the Washington Academy of Sciences 43(7): 203–212.

[B89] Smith AC (1956) Studies of South American plants, XV. American Journal of Botany 43(8): 573–577. 10.1002/j.1537-2197.1956.tb10536.x

[B90] Smith AC Wodehouse RP (1938) The American species of Myristicaceae. Brittonia 2(5): 393–510. 10.2307/2804799

[B91] Soares Maia JG Rodrigues WA (1974) *Virola theiodora* como alucinógena e tóxica. Acta Amazonica 4(1): 21–23. 10.1590/1809-43921974041021

[B92] IUCN Standards and Petitions Subcommittee (2014) Guidelines for using the IUCN Red List categories and criteria. Version 11. Standards and Petitions Subcommittee. http://www.iucnredlist.org/documents/RedListGuidelines.pdf

[B93] Standley PC (1928) Flora of the Panama canal zone. Contributions from the United States National Herbarium 27: 1–416.

[B94] Standley PC (1929) Studies of American plants I. Publications of the Field Museum of Natural History, Botanical Series 4(8): 197–299.

[B95] Standley PC (1930) Flora of Yucatan. Publications of the Field Museum of Natural History, Botanical Series 3(3): 157–492.

[B96] Standley PC (1931) Flora of the Lancetilla Valley, Honduras. Publications of the Field Museum of Natural History, Botanical Series 10: 1–418. 10.5962/bhl.title.2352

[B97] Standley PC (1932) New plants from British Honduras. Publications of the Field Museum of Natural History, Botanical Series 11(4): 127–142. 10.5962/bhl.title.2271

[B98] Standley PC (1937) Flora of Costa Rica. Part II. Publications of the Field Museum of Natural History, Botanical Series 18(2): 399–780.

[B99] Standley PC Steyermark JA (1946) Myristicaceae. In: Standley PC Steyermark JA (Eds) Flora of Guatemala – Part IV.Fieldiana: Botany 24(4), 294–299.

[B100] Steeves RAD (2011) An Intrageneric and Intraspecific Study of Morphological and Genetic Variation in the Neotropical *Compsoneura* and *Virola* (Myristicaceae). PhD. Thesis, The University of Guelph, 295 pp.

[B101] ter Steege H Pitman NCA Sabatier D Baraloto C Salomão RP Guevara JE Phillips OL Castilho CV Magnusson WE Molino J-F Monteagudo A Núñez Vargas P Montero JC Feldpausch TR Coronado ENH Killeen TJ Mostacedo B Vasquez R Assis RL Terborgh J Wittmann F Andrade A Laurance WF Laurance SGW Marimon BS Marimon B-H Guimarães Vieira IC Amaral IL Brienen R Castellanos H Cárdenas López D Duivenvoorden JF Mogollón HF Matos FDA Davila N Garcia-Villacorta R Stevenson Diaz PR Costa F Emilio T Levis C Schietti J Souza P Alonso A Dallmeier F Montoya AJD Fernandez Piedade MT Araujo-Murakami A Arroyo L Gribel R Fine PVA Peres CA Toledo M Aymard CGA Baker TR Ceron C Engel J Henkel TW Maas P Petronelli P Stropp J Zartman CE Daly D Neill D Silveira M Paredes MR Chave J Lima Filho DA Jorgensen PM Fuentes A Schongart J Cornejo Valverde F Di Fiore A Jimenez EM Penuela Mora MC Phillips JF Rivas G van Andel TR von Hildebrand P Hoffman B Zent EL Malhi Y Prieto A Rudas A Ruschell AR Silva N Vos V Zent S Oliveira AA Schutz AC Gonzales T Trindade Nascimento M Ramirez-Angulo H Sierra R Tirado M Umana Medina MN van der Heijden G Vela CIA Vilanova Torre E Vriesendorp C Wang O Young KR Baider C Balslev H Ferreira C Mesones I Torres-Lezama A Urrego Giraldo LE Zagt R Alexiades MN Hernandez L Huamantupa-Chuquimaco I Milliken W Palacios Cuenca W Pauletto D Valderrama Sandoval E Valenzuela Gamarra L Dexter KG Feeley K Lopez-Gonzalez G Silman MR (2013) Hyperdominance in the Amazonian tree flora. Science 342(6156): 1243092 10.1126/science.1243092 24136971

[B102] ter Steege H Vaessen RW Cardenas D Sabatier D Antonelli A Mota de Oliveira S Pitman NCA Jørgensen PM Salomão RP (2016) The discovery of the Amazonian tree flora with an updated checklist of all known tree taxa. Scientific Reports 6(1): 29549 10.1038/srep29549 27406027 PMC4942782

[B103] ter Steege H Mota de Oliveira S Pitman NCA Sabatier D Antonelli A Guevara Andino JE Aymard GA Salomão RP (2019) Towards a dynamic list of Amazonian tree species. Scientific Reports 9(1): 3501 10.1038/s41598-019-40101-y 30837572 PMC6401171

[B104] Thiers B (2019) Index Herbariorum: A global directory of public herbaria and associated staff. New York Botanical Garden’s Virtual Herbarium. http://sweetgum.nybg.org/science/ih/

[B105] Turland NJ Wiersema JH Barrie FR Greuter W Hawksworth DL Herendeen PS Knapp S Kusber W-H Li D-Z Marhold K May TW McNeill J Monro AM Prado J Price MJ Smith GF (2018) International Code of Nomenclature for algae, fungi, and plants (Shenzhen Code) adopted by the Nineteenth International Botanical Congress Shenzhen, China, July 2017. Regnum Vegetabile 159. Koeltz Botanical Books, Glashütten. 10.12705/Code.2018

[B106] Ulloa Ulloa C Acevedo-Rodríguez P Beck S Belgrano MJ Bernal R Berry PE Brako L Celis M Davidse G Forzza RC Gradstein SR Hokche O León B León-Yánez S Magill RE Neill DA Nee M Raven PH Stimmel H Strong MT Villaseñor JL Zarucchi JL Zuloaga FO Jørgensen PM (2017) An integrated assessment of the vascular plant species of the Americas. Science 358(6370): 1614–1617. 10.1126/science.aao0398 29269477

[B107] Vásquez MR Rojas GRDP Monteagudo MAL Valenzuela GL Huamantupa Ch I (2018) Catálogo de los árboles del Perú. Q'euña. Revista de la Sociedad Botánica del Cusco 9: 1–607.

[B108] Vásquez Martínez R (1997) Flórula de las Reservas Biológicas de Iquitos, Perú: Allpahuayo-Mishana, Explornapo Camp, Explorama Lodge. Monographs in Systematic Botany from the Missouri Botanical Garden 63: 1–1046.

[B109] Vásquez Martínez R (2010) Myristicaceae. In: Vásquez Martínez R Rojas Gonzáles R van der Werff H (Eds) Flora del Río Cenepa, Amazonas, Perú. Angiospermae (Gentianaceae–Zingiberaceae).Monographs in Systematic Botany from the Missouri Botanical Garden 2: 1061–1072.

[B110] Villaseñor JL (2016) Checklist of the native vascular plants of Mexico/ Catálogo de las plantas vasculares nativas de México. Revista Mexicana de Biodiversidad 87(3): 559–902. 10.1016/j.rmb.2016.06.017

[B111] Walker JW Walker AG (1979) Comparative Pollen Morphology of the American Myristicaceous Genera *Compsoneura* and *Virola*. Annals of the Missouri Botanical Garden 66(4): 731–755. 10.2307/2398916

[B112] Warburg O (1897) Monographie der Myristicaceen. Nova Acta Academiae Caesareae Leopoldino-Carolinae Germanicae Naturae Curiosorum 68: 1–680.

[B113] Warburg O (1905) Myristicaceae costaricense (XXVI). Repertorium Specierum Novarum Regni Vegetabilis 1(5–6): 71–72. 10.1002/fedr.19050010503

[B114] Williams LO (1964) Tropical American plants, VI. Fieldiana. Botany 31(2): 17–48. 10.5962/bhl.title.2420

